# Genetic diversity, relationships among traits and selection of tropical maize inbred lines for low-P tolerance based on root and shoot traits at seedling stage

**DOI:** 10.3389/fpls.2024.1429901

**Published:** 2024-10-01

**Authors:** Andreia Schuster, Alice Silva Santana, Alison Uberti, Fabíola dos Santos Dias, Helber Moreira dos Reis, Vidomar Destro, Rodrigo Oliveira DeLima

**Affiliations:** ^1^ Department of Agronomy, Universidade Federal de Viçosa, Viçosa, Brazil; ^2^ Corn Breeding Department, Tropical Melhoramento e Genética, Sorriso, Brazil; ^3^ Research and Development Department, Syngenta, Palmas, Brazil; ^4^ Research and Development Department, GDM, Petrolina, Brazil

**Keywords:** *Zea mays*, abiotic stress, selection index, tropical environments, maize germplasm, breeding strategies

## Abstract

The tropical maize breeding for low-P tolerance and good performance under low-P stress environments can be achieved through selection based on root morphology traits at seedling stage. Here, we assessed the genotypic variation and genetic diversity of a panel of 151 tropical maize inbred lines for root and shoot seedling traits, investigated the relationship among traits and selected a set of promising inbred lines for low-P tolerance and performance. We evaluated the inbred lines at seedling stage in a greenhouse experiment under two conditions: applied P (AP) and non-applied P (NAP). A mixed model approach was used to estimate variance components and predict the genotypic values of each inbred line. The genetic diversity among inbred lines based on root and shoot traits was assessed, and correlations were estimated between tested traits under AP and NAP. Our panel of inbred lines showed huge genetic variability for all traits and presented large genetic diversity under both P conditions. Variance components due to the inbred line × P condition interaction were also highly significant (*P* < 0.01) for all traits. Root dry weight (RDW) was positively associated with stalk dimeter (SD), shoot dry weight (SDW) and root length, volume, and area under both P conditions. Also, the SD and SDW were associated with most root traits under AP. Based on low-P tolerance and performance indices, we selected a set of top 20 inbred lines to be used in our maize breeding program. We therefore concluded that there is a significant genetic diversity in the tropical maize inbred lines which have the genetic potential to be use in association mapping studies and also to develop improved low-P tolerant and P-efficient hybrids and maize breeding populations for low-P stress environments.

## 1 Introduction

Maize (*Zea mays* L.) is the largest cereal produced in the world and one of the most important cereal crops for human nutrition, livestock feed and biofuel globally (Erenstein et al., 2022; USDA, 2023). It also plays a very important role in improving food security in developing countries (Grote et al., 2021). Even though maize in the tropical and temperate environments are approximately equals, only 30% of global maize is produced under tropical conditions (Edmeades et al., 2017; Von Pinho et al., 2022; FAO, 2023). In tropical environments, maize production is greatly constrained by adverse environmental conditions, i.e., heat and drought occurrence, and the predominance of poor soils with mineral deficiencies, especially nitrogen (N) and phosphorus (P; Vergutz et al., 2017; Prasanna et al., 2021; Von Pinho et al., 2022). Phosphorus is a vital macronutrient to plant growth and development and the second most limiting nutrient in maize production after N (Vergutz et al., 2017). Its deficiency in soils has strongly contributed to the low maize yield in tropical areas (Vasconcelos et al., 2022; Parentoni et al., 2010). In these areas, the low-P stress may decrease maize yield by around 50-60% compared to optimal P conditions (Fritsche-Neto et al., 2010; Parentoni et al., 2010; Meirelles et al., 2016). Therefore, the development of improved low-P tolerant and P-efficient tropical maize varieties associated with better management practices, such as soil acidity correction and optimization of P fertilizer application in the soil, are urgently required for increasing maize yield across tropical environments and consequently ensuring the security of food production in developing tropical countries (Van de Wiel et al., 2016; Heuer et al., 2017; Vasconcelos et al., 2022; Parentoni et al., 2010; Wang et al., 2024).

Genetic variability for adaptation to low-P environments has been reported in temperate and tropical maize, indicating that good genetic progress can be made in improving maize for low-P tolerance and P-use efficiency (Bayuelo-Jiménez and Ochoa-Cadavid, 2014; Zhang et al., 2015; Spolaor et al., 2018; Li et al., 2021; Parentoni et al., 2010; Vasconcelos et al., 2022; Liang et al., 2023). However, both low-P tolerance and P-use efficiency are complex genetically controlled traits that are governed by several genes with non-additive and additive effects, and they are highly influenced by environmental factors (Parentoni et al., 2010; Mendes et al., 2014; Heuer et al., 2017; Xu et al., 2018; Wang et al., 2019; Weiß et al., 2022). In addition to these, accurately phenotyping a large number of maize genotypes for low-P tolerance and P-efficiency related traits under field conditions is very labor, expensive, time-consuming, and challenging due to the variability in soil properties and the difficulty in accurately assessing root systems in a field setting. On the other hand, greenhouse is an intermediate between the lab and the field, where the growth conditions can be moderately controlled and also available of P can be accurately and consistently managed, without the confounding effects of external environmental variables (Paez-Garcia et al., 2015; Liu et al., 2017b). Thus, genetic improvement of root traits has been proposed as a cheap, fast, and efficient breeding strategy to increase low-P tolerance and/or P-use efficiency in maize for low-P stress environments (Bayuelo-Jiménez et al., 2011; Zhang et al., 2014; Li et al., 2016; Van de Wiel et al., 2016; Lynch, 2022; Mathew and Shimelis, 2022; Liang et al., 2023). Zhu and Lynch (2004) assessed the value of seedling lateral rooting to P-acquisition efficiency in maize and reported that lateral rooting is a useful trait in the screening and breeding of P-efficient maize inbred lines under low-P stress. Zhang et al. (2015) evaluated a large set of maize accessions for low-P tolerance under field conditions. They found that accessions with larger seedling root system were more tolerant to low-P stress than those with smaller root size and suggested that root traits may be successfully used in the evaluation and selection of maize germplasm for low-P tolerance. In a quantitative trait loci (QTL) study, Gu et al. (2016) investigated the genetic association between seedling root traits and P-use efficiency in maize and observed that root traits were genetically associated with P-efficiency acquisition under low-P stress. In another study, Jia et al. (2018) recommended that lateral root density must be considered for the genetic improvement of P-use efficiency in maize under low-P soils. Recently, Salungyu et al. (2020) studied the translation of maize root traits from lab to the field and found that seedling root traits assessed in lab conditions can accurately predict mature roots and plant performance in the field. Therefore, the screening and breeding of maize germplasm based on seedling root traits must be integrated in the routine of the breeding programs to accelerate the development of high low-P tolerant and P-efficient maize varieties, mainly for low-P stress environments in tropical areas.

Currently, around 90% of the global maize production comes from hybrids derived from crossing among inbred lines. As a result, maize breeding programs worldwide have focused their efforts on the development of hybrids (Andorf et al., 2019; Erenstein et al., 2022). The success of any maize breeding program targeting hybrids is tightly associated with the existence of genetic divergence between the parental inbred lines since the magnitude of heterosis expressed in a cross is determined by relative differences in allele frequency between two parents (Falconer and MacKay, 1996). Moreover, the genetic dissection of complex quantitative traits through association mapping requires a germplasm collection that encompass suitable genetic diversity of the traits of interest for the target growing conditions (Flint-Garcia et al., 2005; Cortes et al., 2021). According to Hansey et al. (2011), a maize inbred line panel intended for association mapping must utilize the maximum phenotypic diversity possible. However, there are only three studies on genetic diversity assessment for seedling root and shoot traits in maize, and of these, two were carried out under nonstress conditions (Manavalan et al., 2011; Kumar et al., 2012). In the Torres et al. (2019) study, the authors assessed the genetic diversity for root morphology in a panel of tropical maize under contrasting N conditions and found a larger genetic divergence under low-N stress than under optimal N condition. In addition to genetic diversity, the genetic association within seedling root traits and between root and shoot traits has not been studied yet in tropical maize grown under low-P stress environments. This knowledge is crucial in improving multiple traits simultaneously, as the efficiency of such improvement depends on the understanding of the relationships among the traits of interest (Hazel, 1943; Hallauer et al., 2010). Therefore, further investigation into the genetic diversity and relationship among seedling root traits is necessary in maize, particularly under low-P stress conditions. This research may aid in the breeding of new low-P tolerant and P-use efficient maize varieties, as well as the creation of an inbred line panel for genetic dissection of low-P tolerance in tropical maize through association mapping studies.

The maize breeding program from Universidade Federal de Viçosa (UFV), located in Viçosa (lat. 20°45’14”S; long. 42°52’55”W; alt. 648 m a.s.l.), Minas Gerais state, is the second largest and most important public maize breeding program in Brazil. The purpose of the UFV breeding program is to develop and improve maize germplasm to be cultivated across tropical environments in Brazil, especially under drought and low-N and P stresses. Over the past two decades, we developed a panel of 187 tropical maize inbred lines that represent the current UFV breeding pool. The inbred lines panel has been characterized using molecular markers and agronomic traits, and presented large genotypic and phenotypic diversity, fast linkage disequilibrium decay and the presence of three heterotic groups (Faria et al., 2022). Recently, Uberti et al. (2023) evaluated a set of 190 single-cross hybrids derived from 20 elite inbred lines from our panel across multiple-stress environments and found promising hybrids to be grown across diverse Brazilian conditions. Although most inbred lines from the panel were characterized for N-use efficiency and root morphology under greenhouse and field tropical environments (Rodrigues et al., 2017; Torres et al., 2018, 2019), they have not been phenotyped yet for low-P tolerance, especially under low-P stress conditions. Also, to the best of our knowledge, there are no studies on phenotypic characterization of tropical maize germplasm for low-P tolerance based on seedling root and shoot traits. Therefore, our main goal was to characterize the panel of tropical maize inbred lines from UFV breeding program for root and shoot traits at the seedling stage under optimal and low-P stress conditions. Based on root and shoot traits, our specific objectives were to: i) quantify the genotypic variation and genetic diversity in the inbred lines panel under applied (AP) and non-applied P (NAP) conditions; ii) investigate the relationship among traits under both P conditions; (iii) select tropical maize inbred lines for low-P tolerance and low-P performance based on root and shoot traits; and (iv) discuss breeding strategies for using low-P tolerant inbred lines into our breeding program targeting the development of low-P tolerant and P-efficient hybrids of tropical maize.

## 2 Material and methods

### 2.1 Plant materials

A set of 151 inbred lines representative of tropical maize germplasm from the UFV breeding program was used in our study. The inbred lines represent a sample of the germplasm used in our maize breeding program to develop hybrids, breeding populations, new inbred lines, and populations for QTL mapping and inheritance studies targeting stress environments, such as low-P and N, and drought-stress conditions. The inbred lines were derived from different tropical maize germplasm sources: commercial hybrids, open-pollinated varieties, improved and synthetic populations, and they have recently been characterized based on molecular markers and allocated into three heterotic groups (Faria et al., 2022).

### 2.2 Experimental design

The 151 tropical maize inbred lines were evaluated under two conditions: applied P (AP) and non-applied P (NAP). The experiments were carried out in a greenhouse at UFV in Viçosa, MG state, Brazil (20°45’S; long 42°52’W; alt 640 m asl), during Nov 01, 2020, to Mar 30, 2021. The maize inbred lines seeds were surface sterilized with 10% (v/v) H_2_O_2_ for 30 min, soaked for five hours in saturated CaSO_4_ and washed with sterile water. Then, three seeds of each inbred line were sown into cylindrical plastic plots (15 cm diameter and 50 cm high) and filled with sand. At the V2 leaf collar stage (Abendroth et al., 2011), they were thinned to one plant per plot.

Ten days after sowing, the plants were watered every two days with 0.3 L of nutrient solution which was adapted from Zhang et al. (2014). The AP nutrient solution contained (in mmol L^-1^) 4.0 Ca(NO_3_)_2_, 6.0 KNO_3_, 2.0 MgSO_4_, 1.5 NH_4_H_2_PO_4_ and (in µmol L^-1^) 46 H_3_BO_3_, 7.8 MnSO_4_, 0.32 CuSO_4_, 0.76 ZnSO_4_, 0.016 (NH_4_)_6_Mo_7_O_24_, and 100 Fe-EDTA. The NAP nutrient solution contained 0.75 mmol L^-1^ NH_4_NO_3_ and no N H_4_H_2_PO_4_. On days when the nutrient solution was not supplied, the plants were irrigated with deionized water. Each experiment was conducted with a randomized block design with three replicates per treatment.

Plants were harvested when they reached the V5 leaf collar stage (Abendroth et al., 2011), approximately 35 days after sowing. The shoot was separated from the root system, wrapped in paper bags, and dried in a forced-air oven at 70°C for 72 h. Root systems were washed with deionized water and stored in a solution of 25% alcohol for later image analysis.

### 2.3 Trait measurements

We evaluated 13 shoot and root-related traits across the 151 tropical maize inbred lines under each P condition. For shoot traits, we measured: stalk diameter (SD, mm), measured with an electronic caliper, perpendicular to the soil, approximately five centimeters above the surface; plant height (PH, cm), as the distance from the surface to the stem tip; shoot dry weight (SDW, mg), determined after oven drying at 65°C for 7 days; and daily growth (DG, cm), which was calculated by the ratio between PH and number of days from emergence till harvest. For root traits, we measured: root dry weight (RDW, mg), after oven drying at 65°C for 72 hours; lateral root length (LRL, cm), total root length (TRL, cm), root surface area (RSA, cm^2^), root volume (RV, cm^3^), root average diameter (RAD, mm) and root tissue density (RTD, mg cm^-3^). The RTD was obtained by the ratio between RDW and RV according to Birouste et al. (2014). Finally, we calculated the root:shoot ratio (RSR, mg mg^-1^) from root dry weight and shoot dry weight ratio (RSR), and total dry weight (TDW, mg). The root system was evaluated by image analysis using the WinRHIZO Pro 2009a software (Basic, Reg, Pro & Arabidopsis for Root Measurement) coupled to an EPSON Perfection V700/V750 scanner equipped with additional light, as described by Bouma et al. (2000). Roots with diameters less than 0.50 mm were treated as lateral roots, and roots with diameters greater than 0.50 mm were treated as axial roots, as described by Trachsel et al. (2009).

### 2.4 Statistical analyses

A mixed model implemented in R package “lme4” (Bates et al., 2015) was used to estimate the variance components and to predict the genotypic values of each inbred line under each P conditions and across P conditions (combined analysis). The inbred line was considered as a random effect, while block and P condition were considered as fixed effects. In the combined analysis, the inbred lines by P conditions interaction was considered as a random effect. Variance components were estimated by using a restricted estimation of maximum likelihood, and the genotypic values of inbred lines were predicted using the best linear unbiased predictors (BLUPs; Piepho et al., 2008). A likelihood ratio test deviance analysis was used to test random effects via the chi-square statistic (Resende, 2007). Ranges and mean values were based on BLUPs. The percentage of reduction in response to P stress was estimated as follows: {[(AP−NAP)/AP]×100} . Broad-sense heritability $\left(\hat{\text{h}}\frac{2}{\text{X}}\right)$ on an entry mean basis for each trait under each P condition, and at combined analysis were estimated as follows (Hallauer et al., 2010): $\hat{\text{h}}\frac{2}{\text{X}}=\frac{{\hat{\sigma }}_{\text{G}}^{2}}{{\hat{\sigma }}_{\text{G}}^{2}+\frac{{\hat{\sigma }}^{2}}{\text{r}}}$ and $\hat{\text{h}}\frac{2}{\text{X}}=\frac{{\hat{\sigma }}_{\text{G}}^{2}}{{\hat{\sigma }}_{G}^{2}+\frac{{\hat{\sigma }}_{\text{GxP}}^{2}}{\text{p}}+\frac{{\hat{\sigma }}^{2}}{rp}}$ where ${\hat{\sigma }}_{\text{G}}^{2}$ , ${\hat{\sigma }}_{\text{GxP}}^{2}$ , and ${\hat{\sigma }}^{2}$ , genotypic variance estimates, variance estimates due to inbred line x P condition interaction and error variance estimates, respectively; ‘r’ is the number of replications, and ‘p’ is the number of P conditions. Under NAP and AP, Pearson’s correlation coefficient between traits were estimated using the BLUPs of each trait. Spearman’s correlation coefficients were estimated between pairs of the same traits under AP and NAP conditions. Correlation coefficients were estimated using the *cor.test* function in the R package “stats” (R Core Team, 2021).

To avoid the adverse effects of linear relationships between lines or columns of the matrix, and those of residual correlation on genetic diversity analysis, multicollinearity was diagnosed based on an X’X correlation matrix (Montgomery and Peck, 2001). Multicollinearity diagnostic was carried out by the Variance Inflation Factor (VIF) using the R package “faraway” (Faraway, 2016), and the variance inflation factor value above 10 was considered as an indicator of multicollinearity (Dohoo et al., 2012). In this case, the same traits were discarded under AP and NAP conditions. After that, genetic diversity assessment among the 151 tropical maize inbred lines under AP and NAP was performed using the R package “ape” (Paradis et al., 2004). Firstly, the BLUP of each trait was normalized to avoid effects owing to scaling differences, and thereafter a mean Euclidian distance matrix was generated from root and shoot traits at each P conditions. Then, we used the distance matrix to cluster the tropical maize inbred lines under NAP and AP based on the Unweighted Pair Group Method with Arithmetic Averages (UPGMA; Sokal and Michener, 1958). Mojena (1977) was used to allocate the maize inbred lines into clusters. According to this method, the dendrogram must be cut in function of the mean value of the genetic distance of fusion levels and the standard deviation of the distance values.

We also evaluated the low-P tolerance and the performance under P stress of maize inbred lines using the following indexes (Bayuelo-Jiménez et al., 2011; Zhang et al., 2015; Li et al., 2021): low-P tolerance index (LPTI) and low-P performance index (LPPI). Both LPTI and LPPI indices were estimated using the traits that did not show multicollinearity (Dohoo et al., 2012). Firstly, the BLUP of each trait under NAP was divided by the corresponding BLUP under AP for determining the relative trait value. Then, the relative trait values of all traits were standardized with the means set at zero and the standard deviations set at 1. After that, they were subjected to principal component analysis (PCA), and the ${\text{PC}}_{i}$ whose eigenvalues were equal or greater than one were retained and used to calculate the LPTI as follows $\text{LPTI}=\;\sum_{i=1}^{n}{\text{PC}}_{i}\times {\text{CR}}_{i}$ , where n represents the number of principal components with eigenvalue greater than one and CR (contribution rate) represents the rate for variation of all relative trait values. The LPPI was estimated using the standardized trait value under NAP. The BLUP of each trait under NAP were standardized with the means set at zero and the standard deviations set at 1 and subjected to principal components analysis (PCA). The ${\text{PC}}_{i}$ whose eigenvalues were greater than one was retained and used to calculate the LPPI as follows: $\text{LPPI}=\;\sum_{i=1}^{n}{\text{PC}}_{i}\times {\text{CR}}_{i}$ . Based on these two indexes, we classified the tropical maize inbred lines into four groups: tolerant and good-performance group (TG), the sensitive and good-performance group (SG), the tolerant and poor-performance group (TP), and the sensitive and poor-performance group (SP). Based on the performance of these two indices, LPTI and LPPI, we selected the top 20 (~13%) and bottom five (~3%) tropical maize inbred lines.

## 3 Results

### 3.1 Ranges, means and coefficient of variation under NAP and AP

We observed wide ranges of genotypic values of the inbred lines for all traits under NAP and AP (**Table 1**). However, the P stress affected the genotypic variation among the inbred lines, and the ranges were much larger under AP than under NAP for almost all traits, except for RSR, LRL, RAD and RTD. RSR and RTD showed larger ranges under NAP than under AP; RSR ranged from 0.34 to 0.90, and from 0.24 to 0.48; and RTD ranged from 79.68 to 120.96 mg cm^-3^, and from 72.32 to 94.63 mg cm^-3^, under NAP and AP, respectively. For RAD, inbred lines had similar ranges under both P conditions. Interestingly, the range for LRL was slightly higher under NAP (from 586.47 to 1,829.22 cm) than under AP (from 961.85 to 2,136.32 cm), whereas for TRL, the range was larger under AP. For TRL, genotypic values of inbred lines ranged from 1,026.65 to 2,473.67 cm, and from 1,343.05 to 3,279.28 cm under NAP and AP, respectively.

**Table 1:** Best linear unbiased prediction estimates of ranges and means, coefficient of variation (CV%) and percentage of reduction of the mean of root and shoot-related traits measured in 151 tropical maize inbred lines evaluated under non-applied (NAP) and applied phosphorus (AP) condition.

Trait^1/^	NAP				AP				% reduction of the mean
	Min.	Mean	Max.	CV (%)	Min.	Mean	Max.	CV (%)	
SD (mm)	3.46	4.49	5.61	12.81	5.59	8.70	11.19	8.23	48.32
PH (cm)	5.33	10.99	15.28	13.20	10.55	18.09	26.35	8.49	39.22
SDW (mg)	379.31	665.49	1,001.87	17.79	691.00	1,701.13	3,117.68	17.96	60.88
RDW (mg)	192.06	359.00	552.78	16.67	206.87	525.17	1,010.16	18.65	31.64
RSR (mg mg^-1^)	0.34	0.55	0.90	12.29	0.24	0.32	0.48	15.96	-73.91
TDW (mg)	579.49	1,024.48	1,489.03	16.52	887.76	2,226.29	4,088.35	16.53	53.98
DG (cm)	0.14	0.31	0.43	14.97	0.42	0.70	0.96	8.22	56.34
LRL (cm)	586.47	1,048.99	1,829.22	21.79	961.85	1,533.24	2,136.32	21.04	31.58
TRL (cm)	1,026.65	1,569.73	2,473.67	18.17	1,343.05	2,297.37	3,279.28	17.55	31.67
RSA (cm^2^)	170.78	266.30	379.34	16.03	189.74	426.40	718.97	14.14	37.55
RV (cm^3^)	1.93	3.65	5.99	17.29	2.26	6.38	12.13	16.01	42.81
RAD (mm)	0.45	0.55	0.67	7.72	0.49	0.59	0.70	8.79	7.61
RTD (mg cm^-3^)	79.68	99.15	120.96	8.26	72.32	82.83	94.63	12.18	-19.70

^1/^SD, stalk diameter; PH, plant height; SDW, shoot dry weight; RDW, root dry weight; RSR, root dry weight to shoot dry weight ratio; TDW, total dry weight; DG, daily growth; LRL, lateral root length; TRL, total root length; RSA, root surface area; RV, root volume; RAD, root average diameter, and RTD, root tissue density.

All measured traits were affected by P conditions, and most of them had their means negatively affected by the P deficiency, except RSR and RTD. We observed that the genotypic means of the shoot traits SD (48.32%), PH (39.22%), SDW (60.88%) and DG (56.34%), and the root traits RSA (37.55%) and RV (42.81%), and the trait TDW (53.98%) were more substantially decreased in response to P stress (percentage of reduction higher than 35%). The RSR was the trait most positively affected by the P deficiency (-73.91% reduction in response to P stress). Regarding the genotypic means of root length, both LRL and TLR had their means decreased at 31% in response to P stress.

The coefficient of variation (CV) showed similar values between P conditions and ranged from 7.72% (RAD) to 21.79% (LRL), and from 8.22% (DG) to 21.04% (LRL) under NAP and AP conditions, respectively. Overall, traits showed low CVs, except for LRL, which exhibited CVs values greater than 20% under both P conditions.

### 3.2 Variance components and broad-sense heritability estimates

Variance components due to inbred lines were highly significant (*P* < 0.01) by the likelihood ratio test for all root and shoot traits under NAP, AP, and across P conditions (**Table 2**). Also, variance components due to inbred lines × P conditions interaction were highly significant (*P* < 0.01) for all traits, and therefore the inbred lines had different relative performance across P conditions for all tested traits. In general, the variance component estimates due to inbred lines were much higher under AP than under NAP, except for the traits RSR, RAD and RTD. The estimates of broad-sense heritability $\left(\hat{\text{h}}\frac{2}{\text{X}}\right)$ were intermediate to high under both P conditions, and most traits exhibited $\hat{\text{h}}\frac{2}{\text{X}}$ values greater than 0.80. The $\hat{\text{h}}\frac{2}{\text{X}}$ values ranged from 0.74 (SD) to 0.88 (RSR) and from 0.55 (RTD) to 0.91 (PH and TDW) under NAP and AP, respectively, and they were higher under AP than under NAP for most traits, except for RSR, LRL, TRL, RAD and RTD. These traits showed greater $\hat{\text{h}}\frac{2}{\text{X}}$ values under NAP than under AP. In the combined analysis, the inbred lines × P conditions interaction negatively affected $\hat{\text{h}}\frac{2}{\text{X}}$ , and the $\hat{\text{h}}\frac{2}{\text{X}}$ values were low to intermediate (<0.70) across P conditions. They ranged from 0.29 (SD) to 0.67 (RAD).

**Table 2:** Variance components estimates due to inbred lines ( ${\hat{\sigma }}_{\text{G}}^{2}$ ) and inbred line x P condition interaction ( ${\hat{\sigma }}_{\text{GxP}}^{2}$ ), and estimates of residual variance ( ${\hat{\sigma }}^{2}$ ) and broad-sense heritability ( $\hat{\text{h}}\frac{2}{\text{X}}$ ) for root and shoot-related traits measured in 151 tropical maize inbred lines evaluated under non-applied and applied P.

Trait^1/^	Non-applied P			Applied P			Combined analysis			
	${\hat{\sigma }}_{\text{G}}^{2}$	${\hat{\sigma }}^{2}$	$\hat{\text{h}}$ $\frac{2}{\text{X}}$	${\hat{\sigma }}_{\text{G}}^{2}$	${\hat{\sigma }}^{2}$	$\hat{\text{h}}\frac{2}{\text{X}}$	${\hat{\sigma }}_{\text{G}}^{2}$	${\hat{\sigma }}_{\text{GxP}}^{2}$	${\hat{\sigma }}^{2}$	$\hat{\text{h}}\frac{2}{\text{X}}$
SD (mm)	0.31*^2/^	0.33	0.74	1.35*	0.51	0.89	0.16*	0.67*	0.42	0.29
PH (cm)	2.78*	2.11	0.80	8.26*	2.36	0.91	2.76*	2.76*	2.23	0.61
SDW (mg)	19,301.00*	14,023.00	0.81	283,212.00*	93,362.00	0.90	31,094.00*	120,162.00*	53,692.00	0.31
RDW (mg)	6,216.00*	3,582.00	0.84	20,261.00*	9,588.00	0.86	4,208.00*	9,031.00*	6,585.00	0.43
RSR (mg mg^-1^)	1.10×10^-2^*	4.66×10^-3^	0.88	2.32×10^-3^*	2.59×10^-3^	0.73	3.46×10^-3^*	3.19×10^-3^*	3.62×10^-3^	0.61
TDW (mg)	38,787.00*	28,632.00	0.80	438,747.00*	135,469.00	0.91	48,953.00*	189,815.00*	82,051.00	0.31
DG (cm)	2.77×10^-3^*	2.10×10^-3^	0.80	8.16×10^-3^*	3.31×10^-3^	0.88	2.30×10^-3^*	3.16×10^-3^*	2.70×10^-3^	0.53
LRL (cm)	60,534.00*	52,250.00	0.78	70,003.00*	104,06	0.67	38,754.00*	26,515.00*	78,154.00	0.60
TRL (cm)	92,465.00*	81,310.00	0.77	165,270.00*	162,57	0.75	64,782.00*	64,086.00*	121,939.00	0.55
RSA (cm^2^)	2,387.00*	1,822.00	0.80	8,260.00*	3,637.00	0.87	2,186.00*	3,14*	2,730.00	0.52
RV (cm^3^)	0.60*	0.40	0.82	2.79*	1.04	0.89	0.67*	1.02*	0.72	0.51
RAD (mm)	2.50×10^-3^*	1.79×10^-3^	0.81	1.83×10^-3^*	2.72×10^-3^	0.67	1.47×10^-3^*	6.95×10^-4^*	2.25×10^-3^	0.67
RTD (mg cm^-3^)	98.63*	67.04	0.82	40.90*	101.80	0.55	46.56*	23.21*	84.41	0.64

^1/^SD, stalk diameter; PH, plant height; SDW, shoot dry weight; RDW, root dry weight; RSR, root dry weight to shoot dry weight ratio; TDW, total dry weight; DG, daily growth; LRL, lateral root length; TRL, total root length; RSA, root surface area; RV, root volume; RAD, root average diameter, and RTD, root tissue density. ^2/^* significant at 0.01 by the likelihood ratio test.

### 3.3 Genotypic correlations

**Figure 1:**
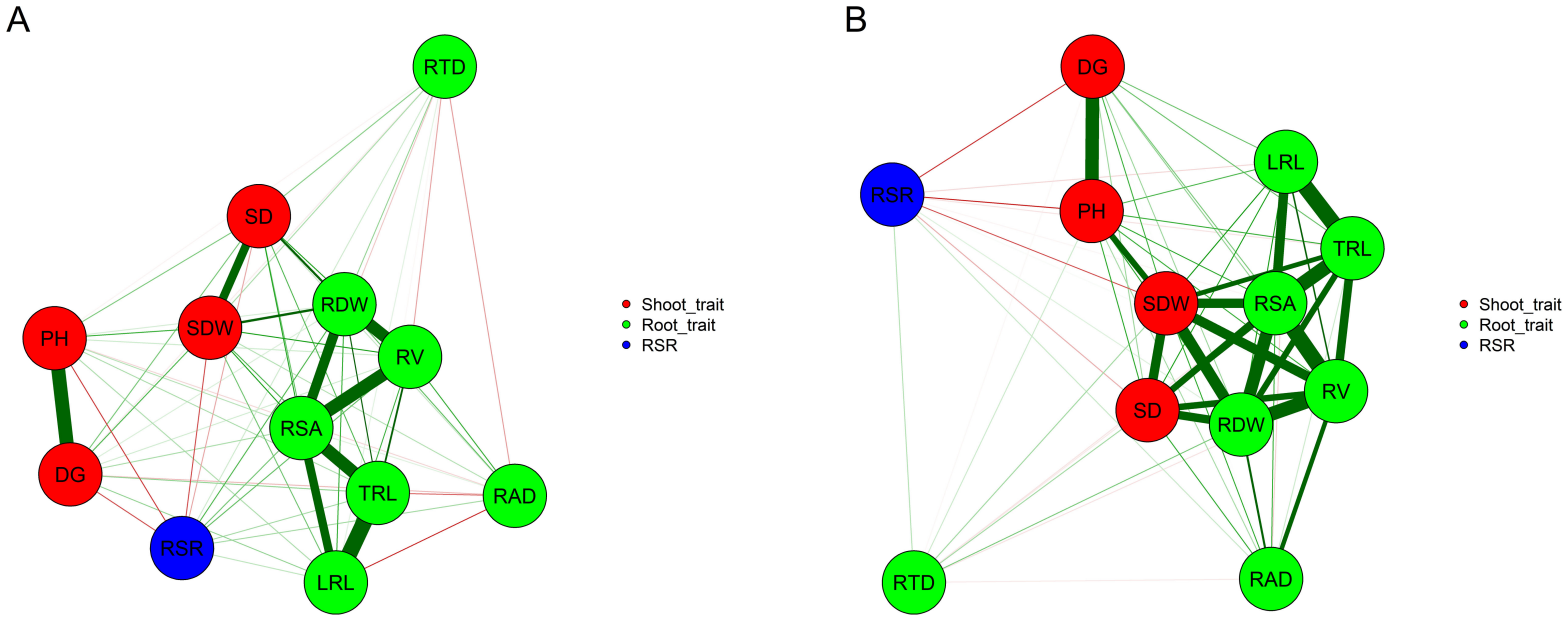
Correlation network between pairs of vectors of genetic values of the traits across 151 tropical maize inbred lines evaluated under non-applied P **(A)** and applied P **(B)**. Red and green lines represent negative and positive correlations, respectively. Line width is proportional to the strength of the correlation. SD: stalk diameter (mm); PH: plant height (cm); SDW: shoot dry weight (mg); RDW: root dry weight (mg); RSR: root dry weight to shoot dry weight ratio (mg mg^-1^); DG, daily growth (cm); LRL, lateral root length (cm); TRL, total root length (cm); RSA, root surface area (cm^2^); RV, root volume (cm^3^); RAD, root average diameter (mm) and RTD, root tissue density (mg cm^-3^).

Most Pearson’s correlation coefficients between traits were positive and significant at *P* < 0.01 under both P conditions, but P stress affected the associations between traits, and genotypic correlations were generally smaller under NAP (bellow diagonal) than under AP (above diagonal) conditions (**Table 3**, **Figure 1**). Also, the effect of low-P stress was more pronounced on the correlations estimates between shoot and root traits. For example, the genotypic correlations estimates between SD and the root traits RDW ( $\hat{\text{r}}$ =0.63 and 0.78), LRL ( $\hat{\text{r}}$ =0.35 and 0.54), TRL ( $\hat{\text{r}}$ =0.41 and 0.64), RSA ( $\hat{\text{r}}$ =0.52 and =0.73), RV ( $\hat{\text{r}}$ =0.53 and 0.74) and RAD ( $\hat{\text{r}}$ =0.13^ns^ and 0.48); and between SDW and the root traits RDW ( $\hat{\text{r}}$ =0.64 and 0.85), LRL ( $\hat{\text{r}}$ =0.33 and 0.58), TRL ( $\hat{\text{r}}$ =0.40 and 0.69), RSA ( $\hat{\text{r}}$ =0.53 and =0.80), RV ( $\hat{\text{r}}$ =0.55 and $\hat{\text{r}}$ =0.81), and RAD ( $\hat{\text{r}}$ =0.17ns and 0.50) were much higher under AP than under NAP. Moreover, PH showed no correlation with any root traits under NAP, whereas under AP, it was correlated significantly with RDW (0.49), LRL (0.42), TRL (0.47), RSA (0.49) and RV (0.46).

**Table 3:** Estimates of Pearson correlation coefficients between pair of vectors of genetic values of the traits across 151 tropical maize inbred lines evaluated under non-applied P (below diagonal) and applied P (above diagonal); and estimates of Spearman correlation coefficients between pairs of the same traits under non-applied and applied P (diagonal).

Trait^1/^	SD	PH	SDW	RDW	RSR	TDW	DG	LRL	TRL	RSA	RV	RAD	RTD
SD (mm)	0.22*^2/^	0.50*	0.80*	0.78*	-0.19	0.82*	0.23*	0.54*	0.64*	0.73*	0.74*	0.48*	0.21*
PH (cm)	0.30*	0.43*	0.72*	0.49*	-0.54*	0.69*	0.91*	0.42*	0.47*	0.49*	0.46*	0.21	0.12
SDW (mg)	0.78*	0.47*	0.36*	0.85*	-0.42*	0.99*	0.46*	0.58*	0.69*	0.80*	0.81*	0.50*	0.21*
RDW (mg)	0.63*	0.10	0.64*	0.25*	0.08	0.91*	0.24*	0.58*	0.72*	0.88*	0.92*	0.63*	0.31*
RSR (mg mg^-1^)	-0.21*	-0.46*	-0.42*	0.41*	0.51*	-0.32*	-0.48*	-0.09	-0.07	-0.02	0.02	0.12	0.17
TDW (mg)	0.80*	0.37*	0.95*	0.84*	-0.14	0.32*	0.42*	0.59*	0.72*	0.84*	0.86*	0.54*	0.24*
DG (cm)	0.31*	0.93*	0.41*	0.09	-0.41*	0.32*	0.35*	0.34*	0.34*	0.31*	0.25*	0.01	-0.02
LRL (cm)	0.35*	0.19	0.33*	0.48*	0.15	0.42*	0.25*	0.45*	0.97*	0.81*	0.61*	-0.11	0.00
TRL (cm)	0.41*	0.19	0.40*	0.60*	0.21*	0.52*	0.25*	0.98*	0.40*	0.93*	0.78*	0.12	-0.05
RSA (cm^2^)	0.52*	0.17	0.53*	0.83*	0.32*	0.70*	0.20	0.79*	0.89*	0.37*	0.96*	0.46*	-0.06
RV (cm^3^)	0.53*	0.11	0.55*	0.88*	0.36*	0.73*	0.11	0.45*	0.62*	0.90*	0.39*	0.68*	-0.07
RAD (mm)	0.13	-0.10	0.17	0.36*	0.21*	0.26*	-0.16	-0.51*	-0.35*	0.07	0.46*	0.49*	-0.05
RTD (mg cm^-3^)	0.27*	-0.02	0.19	0.25*	0.11	0.23*	-0.04	0.07	-0.01	-0.13	-0.21*	-0.23*	0.46*

^1/^SD, stalk diameter; PH, plant height; SDW, shoot dry weight; RDW, root dry weight; RSR, root dry weight to shoot dry weight ratio; TDW, total dry weight; DG, daily growth; LRL, lateral root length; TRL, total root length; RSA, root surface area; RV, root volume; RAD, root average diameter, and RTD, root tissue density. ^2/^* Significant at P = 0.01. Pearson correlation coefficients greater than 0.60 are highlighted in bold. Cells shaded along the diagonal represent the Spearman correlation.

Under both P conditions, RDW presented moderate-to-strong correlations with the shoot traits SD ( $\hat{\text{r}}$ =0.63 and 0.78) and SDW ( $\hat{\text{r}}$ =0.64 and 0.85), and the root traits TRL ( $\hat{\text{r}}$ =0.60 and 0.72), RSA ( $\hat{\text{r}}$ =0.83 and 0.84), and RV ( $\hat{\text{r}}$ =0.88 and 0.86), under NAP and AP, respectively. Moreover, the shoot traits SD and SDW were also strongly positively correlated with RDW ( $\hat{\text{r}}$ =0.78 and $\hat{\text{r}}$ =0.85), TRL ( $\hat{\text{r}}$ =0.64 and $\hat{\text{r}}$ =0.69), RSA ( $\hat{\text{r}}$ =0.73 and $\hat{\text{r}}$ =0.88) and RV ( $\hat{\text{r}}$ =0.74 and $\hat{\text{r}}$ =0.82 with SD and SDW, respectively). In contrast, RSR showed weak-to-moderate correlation with root traits under NAP, and no association with them under AP. For shoot traits, RSR correlated significantly and negatively with PH ( $\hat{\text{r}}$ =-0.46 and -0.54 under NAP and AP, respectively) and SDW ( $\hat{\text{r}}$ =-0.42 under both P conditions), and with SD ( $\hat{\text{r}}$ =-0.21) under NAP. Interestingly, RAD presented moderate and negative correlation with root length ( $\hat{\text{r}}$ =-0.51 and -0.35 with LRL and TRL, respectively) under NAP, but no correlation with these traits under AP. Estimates of Spearman correlations coefficients between NAP and AP conditions were significant and positive at *P*<0.01 for all traits, but the presence of inbred lines × P conditions interaction changed the ranking of inbred lines, and the estimates of Spearman correlations were low-to-moderate (**Table 3**, diagonal). They ranged from 0.22 (SD) to 0.51 (RSR) and most of them were smaller than 0.45.

### 3.4 Genetic diversity

**Figure 2:**
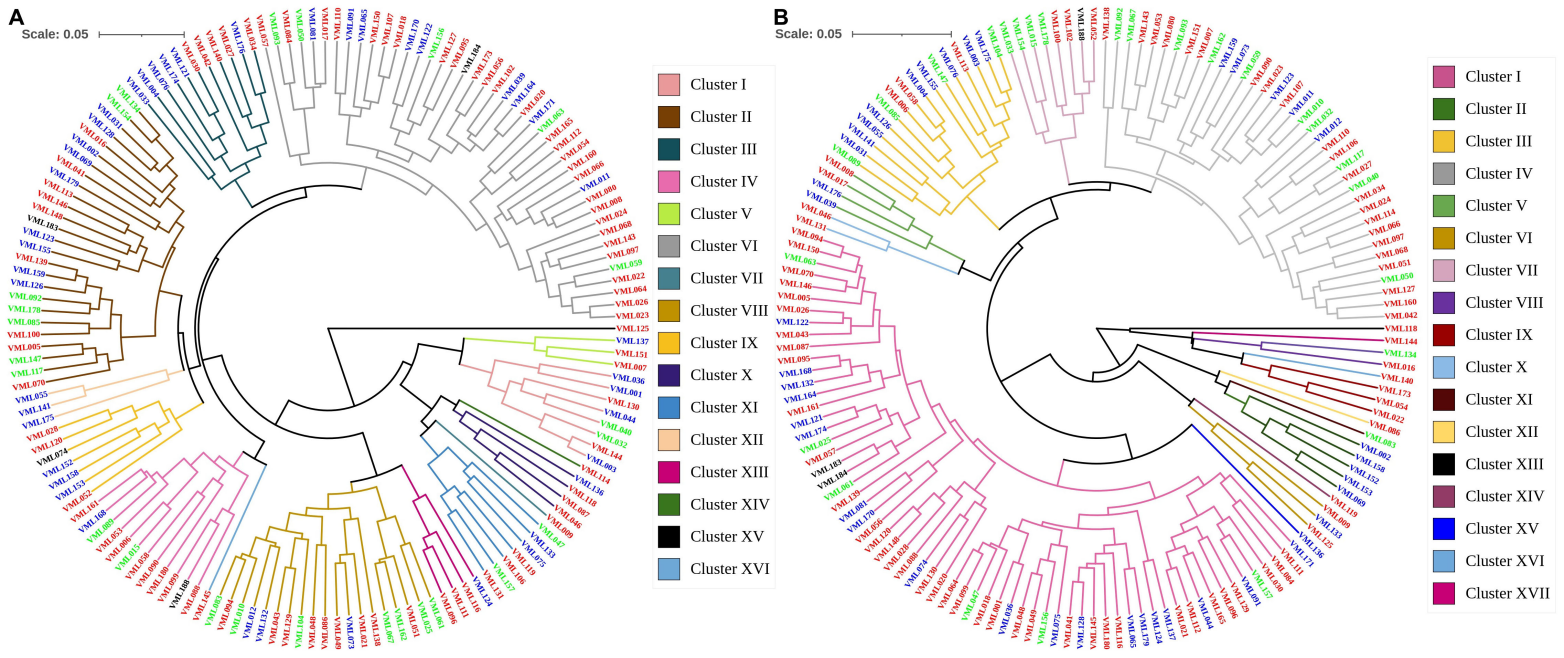
Dendrograms from unweighted pair-group method of arithmetic clustering for 151 tropical maize inbred lines using standardized mean Euclidean distance based on eight traits: stalk diameter, plant height, root:shoot dry weight ratio, daily growth, lateral root length, root volume, root average diameter and root tissue density measured under non-applied P **(A)** and applied P **(B)**. Names of inbred lines that are written in red refer to lines that belong to the heterotic group I, in blue from heterotic group II and in green from heterotic group III.

We detected severe multicollinearity in the X’X correlation matrix under NAP and AP (data not shown) and discarded five traits with redundant information and a large amount of shared variance. The traits were discarded based on the variance inflation factor (VIF) value (Dohoo et al., 2012), and the same traits were discarded under both P conditions. Thus, under both P conditions, the genetic diversity assessment among the 151 tropical maize inbred lines was performed based on eight traits: SD, PH, RSR, DG, LRL, RV, RAD and RTD. Based on Mojena (1977), the UPGMA dendogram grouped the 151 tropical maize inbred lines into 16 clusters under NAP (cut-off point= 1.3) and 17 clusters under AP (cut-off point= 1.3; **Figure 2**). Under NAP, the genetic diversity clusters VI, II and VIII were the largest ones and consisted of 43, 26 and 19 inbred lines, respectively. On the other hand, clusters VII (VML009), XIV (VML114), XV (VML125) and XVI (VML145) consisted of only one inbred line each. Clusters II and VI can be divided into two and three subgroups, respectively, with 11 and 15 (cluster II), and 6, 18 and 19 (cluster VI) inbred lines into each one. The number of inbred lines allocated into the other nine genetic diversity clusters ranged from three (V and VIII) to 12 (IV). Concerning to genetic distance among inbred lines, VML144 and VML003 (genetic distance of 0.023; **Supplementary Table S1**) were the most similar lines and were allocated into cluster I. VML125 and VML036 (genetic distance of 0.542) were the most genetically distant lines and were allocated into clusters XV and I, respectively.

Under AP, the genetic diversity clusters I, IV and III were the largest clusters, and they consisted of 64, 37 and 16 tropical maize inbred lines, respectively (**Figure 2**). The clusters VIII (VML134 and VML16), XVI (VML046 and VML131), with two inbred lines, and X (VML140), XI (VML083), XII (VML086), XIII (VML118), XIV (VML119), XV (VML136) and XVII (VML144), with one line, were the smallest genetic clusters. The largest clusters I and IV can be divided into two and three subgroups, respectively, with 33 and 31 inbred lines (I), and with two, 17 and 18 lines (IV) into each subgroup. The size of other clusters was small to medium and the number of lines in each cluster ranged from three (IX) to eight (VII). Also, the VML094 and VML150 (genetic distance of 0.021; **Supplementary Table S1**), allocated into cluster I, were the most similar inbred lines, and VML002 and VML144 (distance of 0.648) were the most genetically distant lines and were allocated into clusters II and XVII, respectively. Moreover, there was no concordance between heterotic grouping of the inbred lines and our results of clustering analysis based on shoot and root traits, irrespective of P conditions.

### 3.5 Inbred lines performance for LPTI and LPPI

**Figure 3:**
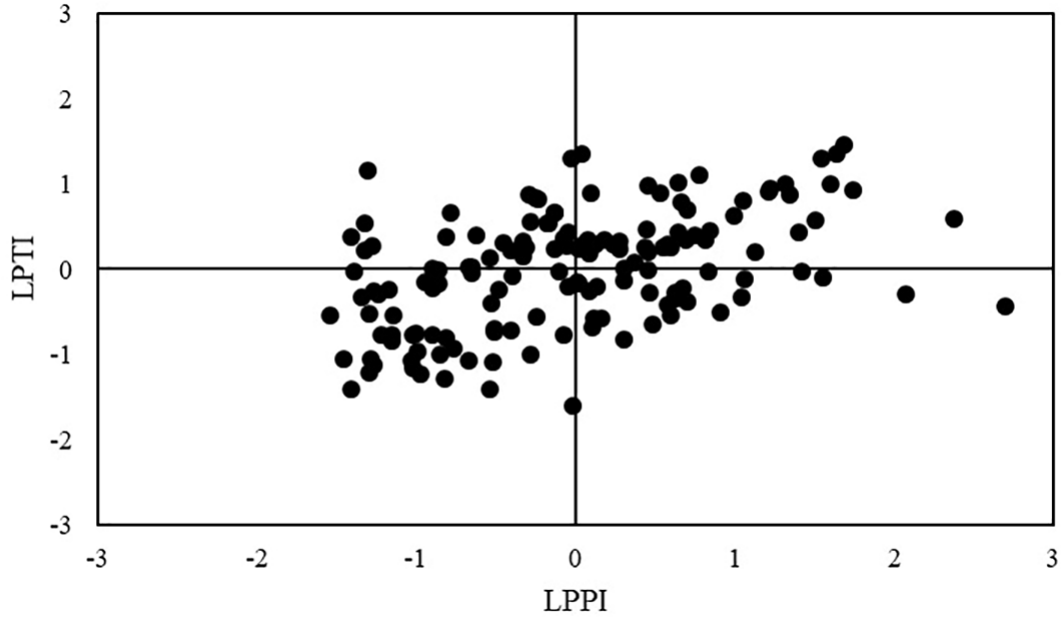
Classification of 151 tropical maize inbred lines for their responses to low-P stress based on LPTI and LPPI indexes. TG, tolerant and good-performance group; SG, sensitive and good-performance group; TP, tolerant and poor-performance group; SP, sensitive and poor-performance group.

The LPPI was moderately positively associated with LPTI ( $\hat{r}$ = 0.47, *P* < 0.01), and thus maize inbred lines that had good performance under NAP also showed high low-P tolerance. In relation to association among indexes and tested traits, LPPI was strongly positively (*P* < 0.01) correlated with SD ( $\hat{\text{r}}$ = 0.83), LRL ( $\hat{\text{r}}$ = 0.71), SDW ( $\hat{\text{r}}$ = 0.75), RDW ( $\hat{\text{r}}$ = 0.77), TDW ( $\hat{\text{r}}$ = 0.83), TRL ( $\hat{\text{r}}$ = 0.75) and RSA ( $\hat{\text{r}}$ = 0.76) under NAP, but it showed weak ( $\hat{\text{r}}$ <0.25) or no correlation with traits under AP condition (**Table 4**). Concerning to LPTI, it was moderately positively correlated with SD ( $\hat{\text{r}}$ = 0.43), DG ( $\hat{\text{r}}$ = 0.47), RV ( $\hat{\text{r}}$ = 0.28), RDW ( $\hat{\text{r}}$ = 0.34) and TDW ( $\hat{\text{r}}$ = 0.31) and showed weak or no significant correlation with other traits under NAP. Conversely, LPTI was moderately negatively correlated with almost all traits under AP condition, except with RSR, which presented positive correlation, and RTD, which present no significant correlation. The LPTI values of maize inbred lines were plotted against their LPPI values, and based on the relationship between them, the 151 inbred lines were classified into four groups (**Supplementary Table S2**; Bayuelo-Jiménez et al., 2011; Zhang et al., 2015; Li et al., 2021): forty-seven (31%) inbred lines were classified into tolerant to low-P stress and good-performance under P stress group (TG), twenty-seven (18%) inbred lines were classified into tolerant and poor-performance group (TP), thirty (20%) inbred lines were classified into sensitive and good-performance group (SG), and forty-seven (31%) inbred lines were classified into sensitive and poor-performance group (SP, **Figure 3**). Under NAP, TG group had the highest genotypic means for SD (4.86 mm), RDW (394.23), RSR (0.57), DG (0.33 cm), RV (4.08 cm^3^), RAD (0.55 mm) and RTD (102.63 mg cm^-3^), whereas SG group had the highest genotypic means for PH (11.68 cm), SDW (752.49 mg) and LRL (1,205.17 cm), TRL (1,759.44 cm; **Table 5**). Inbred lines classified as sensitive and with poor performance (SP group) showed the smallest genotypic means for all shoot and most root traits, except for RAD and RTD. Under AP, SG group had the highest genotypic means, whereas TP group had the smallest genotypic means for almost all traits.

**Table 4:** Estimates of Pearson correlation coefficients among both LPTI and LPPI indexes and BLUPs of 13 root and shoot traits measured in 151 tropical maize inbred lines evaluated under non-applied P and applied P.

Traits	Non-applied P		Applied P	
	LPTI	LPPI	LPTI	LPPI
Stalk diameter (mm)	0.43^*^	0.83^*^	-0.66^*1/^	0.12
Plant height (cm)	0.25^*^	0.49^*^	-0.55^*^	0.00
Shoot dry weight (mg)	0.25^*^	0.75^*^	-0.65^*^	0.11^*^
Root dry weight (mg)	0.34^*^	0.77^*^	-0.62^*^	0.17^*^
Root:shoot dry weight ratio	0.09	0.00	0.14	0.05
Total dry weight (mg)	0.31^*^	0.83^*^	**-0.66**	0.13^*^
Daily growth (cm)	0.47^*^	0.52^*^	-0.34^*^	0.06
Lateral root length (cm)	0.19	0.71^*^	-0.47^*^	0.22
Total root length (cm)	0.22^*^	0.75^*^	-0.54^*^	0.21^*^
Root surface area (cm^2^)	0.28^*^	0.76^*^	-0.61^*^	0.19^*^
Root volume (cm^3^)	0.28^*^	0.62^*^	-0.60^*^	0.16
Root average diameter (mm)	0.11	-0.11	-0.40^*^	-0.03
Root tissue density (mg cm^-3^)	0.14	0.34^*^	-0.13	0.08

^1/*^ Significant at P = 0.01. Pearson correlation coefficients greater than 0.60 are highlighted in bold.

**Table 5:** Best linear unbiased prediction estimates of means and ranges for shoot and root related traits measured in 151 tropical maize inbred lines allocated into three heterotic groups and evaluated under non-applied and applied P conditions.

Groups	SD^1/^	PH	SDW	RDW	RSR	DG	LRL	TRL	RV	RAD	RTD
Non-applied P											
TG	**4.86** ^2/^ 4.30-5.61	11.51 8.58-14.30	740.06 540.31-1,001.87	**394.23** 301.12-552.78	**0.57** 0.40-0.83	**0.33** 0.23-0.43	1,167.72 770.98-1,829.22	1,657.81 1,266.64-1,964.99	**4.08** 2.70-5.99	0.55 0.45-0.67	**102.63** 79.68-120.96
SG	4.74 4.17-5.47	**11.68** 9.43-15.28	**752.49** 604.72-945.51	384.72 298.32-552.78	0.52 0.34-0.69	0.32 0.25-0.41	**1,205.17** 912.94-1,615.29	**1,759.44** 1,351.83-2,219.48	3.87 3.01-5.29	0.53 0.47-0.59	100.43 85.09-115.77
TP	4.31 3.83-4.88	10.98 8.66-13.55	608.99 513.48-787.19	333.64 234.01-432.54	0.56 0.40-0.90	0.32 0.24-0.42	915.38 680.07-1,212.00	1,418.58 1,058.82-1,861.92	3.51 2.52-4.66	**0.57** 0.48-0.65	96.30 85.68-118.35
SP	4.07 3.46-4.98	10.10 5.33-12.70	570.14 379.31-838.18	284.11 192.06-405.98	0.55 0.38-0.76	0.27 0.14-0.35	914.16 586.47-1,299.67	1,293.35 1,026.65-1,561.25	3.15 1.93-4.50	0.54 0.45-0.66	96.43 80.67-118.67
Applied P											
TG	8.43 6.32-10.82	17.07 12.07-26.35	1,548.76 748.06-3,078.63	478.48 206.87-664.66	0.32 0.24-0.48	0.68 0.52-0.96	1,517.66 1,055.94-2,061.59	2,202.00 1,398.90-3,043.21	6.04 2.26-11.05	0.58 0.49-0.70	82.59 72.74-93.36
SG	**9.66** 8.02-10.92	**19.94** 16.15-26.35	**2,190.85** 1,522.92-3,117.68	**658.23** 454.48-1,010.16	031 0.24-0.45	**0.74** 0.61-0.96	**1,703.41** 1,281.81-2,136.32	**2,606.47** 1,894.84-3,279.28	**7.86** 5.02-12.13	**0.61** 0.53-0.69	**84.16** 74.61-93.41
TP	7.62 5.59-8.97	16.86 12.95-22.15	1,280.98 691.00-1,931.37	413.11 218.39-572.53	**0.33** 0.25-0.40	0.68 0.56-0.77	1,339.00 961.85-1,634.96	1,969.94 1,343.05-2,422.05	5.06 2.73-6.30	0.58 0.51-0.65	81.84 72.32-94.63
SP	9.02 6.76-11.19	18.87 10.55-24.10	1,818.87 1,000.34-2,646.16	516.10 324.92-768.31	031 024-0.46	0.71 0.42-0.91	1,563.12 1,263.61-2,080.77	2,239.21 1,807.01-2,534.86	6.61 3.84-9.86	0.60 0.53-0.67	82.88 73.05-89.85

^1/^SD, stalk diameter (mm); PH, plant height (cm); SDW, shoot dry weight (mg); RDW, root dry weight (mg); RSR, root to shoot dry weight ratio; DG, daily growth (cm); LRL, lateral root length (cm); TRL, total root length; RV, root volume (cm^3^); RAD, root average diameter (mm) and RTD, root tissue density (mg cm^-3^). ^2/^Mean, minimum and maximum of the genotypic values, respectively. Bold values refer to the group that had the highest genotypic mean.

The top 10 (~7%) inbred lines for low-P performance (from highest LPPI to lowest) were: VML036, VML121, VML076, VML030, VML118, VML144, VML004, VML046, VML001 and VML119 (**Figure 3**; **Supplementary Table S2**). The lines VML118 and VML144, which were selected among the top 10 for LPPI, were sensitive to low-P stress (negative LPTI values). Concerning to low-P tolerance, the top 10 inbred lines tolerant to low-P stress (from highest LPTI to lowest) were: VML002, VML158, VML153, VML152, VML137, VML036, VML121, VML119, VML088 and VML030, and, among them, the lines VML158, VML152 and VML088 showed poor performance under low-P stress (negative LPPI values). Based on both LPTI and LPPI values, we selected the top 20 (~13%) tropical maize inbred lines for low-P stress performance and tolerance (**Table 6**). We observed that among them, VML036, VML121, VML119 and VML030 were among the top 10 inbred lines for both LPPI and LPTI indexes. Interestingly, maize inbred lines that had the highest values for both LPTI and LPPI indexes belong to heterotic groups I and II, and there was not any selected inbred line from heterotic group III. In relation to genetic diversity, the top 20 inbred lines were grouped into nine and seven divergent clusters under NAP and AP, respectively. The genotypic means of the top 20 inbred lines were greater than overall mean for all root and shoot traits under NAP, but they were smaller than overall mean under AP. We also observed coincidence among the top 20 inbred lines selected based on indexes and their performance for most root and shoot traits under NAP, except for PH. Thus, we highlighted the top inbred lines VML153, VML137, VML119, VML130, and VML001, which were also among the best 20 lines for LRL, the top VML121, VML030, VML133, VML179, VML087, VML009 and VML113 were also selected for RDW, and the top VML121, VML030, VML001, VML087 and VML004 were also selected for SDW (**Supplementary Table S2**). Due to the moderate correlation between SD and LPTI ( $\hat{\text{r}}$ = 0.43) and strong correlation between SD and LPPI ( $\hat{\text{r}}$ = 0.83), eleven out of 20 top inbred lines based on the indexes were also among the 20 best inbred lines for SD under NAP. Moreover, the top inbred lines VML121 (SD, SDW, RDW and RV), VML133 (SD, RDW, RSR and RV), VML009 (SD, RDW, RSR and RTD), VML004 (SD, SDW, RDW, RV) and VML042 (SD, SDW, RV and RAD) were also coincidentally selected for four traits under NAP. Conversely, the top 20 inbred lines selected based on the indexes were not among the best 20 lines for most root and shoot traits under AP condition, mainly for traits that were negatively correlated with both LPTI and LPPI.

**Table 6:** Heterotic groups (HG), genetic diversity cluster (GD) and predicted genotypic means for ten shoot and root traits of the top 20 and bottom five tropical maize inbred lines selected based on LPTI and LPPI.

Lines	HG	GD	SD^1/^	PH	SDW	RDW	RSR	DG	LRL	RV	RAD	RTD	GD	SD	PH	SDW	RDW	RSR	DG	LRL	RV	RAD	RTD
Top 20 lines		Non-Applied P											Applied P										
VML002	2	2	4.56	11.96	540.31	303.91	0.59	0.35	1,215.18	2.70	0.45	114.87	2	6.32	13.56	748.06	206.87	0.24	0.56	1,055.94	2.26	0.49	82.33
VML153	2	9	4.99	13.16	744.26	303.91	0.41	0.43	1,029.38	2.93	0.49	104.87	2	6.78	15.18	946.28	284.61	0.30	0.68	1,262.95	3.40	0.52	82.32
VML137	2	5	5.51	9.93	768.41	371.02	0.48	0.29	1,482.44	3.82	0.48	97.39	1	7.86	13.56	985.32	362.35	0.36	0.57	1,319.05	4.11	0.55	87.41
VML036	2	1	4.77	12.01	763.04	438.13	0.57	0.38	1,829.22	4.31	0.47	101.55	1	7.63	13.96	1,225.59	434.33	0.35	0.62	1,657.50	5.59	0.55	79.66
VML121	2	3	5.61	12.36	956.25	485.67	0.50	0.35	1,228.44	4.67	0.56	103.86	1	7.83	17.95	1,324.70	417.05	0.31	0.75	1,386.64	5.26	0.59	80.40
VML119	1	11	4.62	11.03	626.18	499.65	0.82	0.32	1,406.66	4.50	0.53	109.66	14	7.31	15.72	937.27	454.48	0.48	0.65	1,544.46	4.88	0.55	89.29
VML030	1	3	5.45	11.72	899.90	508.04	0.56	0.35	1,195.23	4.60	0.57	109.51	1	8.23	15.36	1,483.87	535.10	0.36	0.67	1,533.63	5.96	0.59	87.34
VML133	2	11	4.72	9.06	642.29	524.82	0.83	0.27	1,132.24	4.71	0.60	109.97	6	7.55	12.07	1,048.39	460.24	0.43	0.52	1,367.10	6.02	0.61	78.83
VML130	1	1	4.35	11.45	545.68	357.04	0.67	0.39	1,388.45	4.00	0.51	89.21	1	6.95	15.97	1,129.48	373.87	0.33	0.71	1,296.40	4.69	0.58	80.06
VML174	2	3	5.24	11.88	779.14	410.17	0.53	0.37	1,152.01	4.02	0.55	101.72	1	7.94	17.03	1,258.62	396.90	0.31	0.72	1,323.89	4.95	0.58	81.49
VML001	2	1	4.47	11.66	717.42	415.76	0.58	0.36	1,647.33	3.91	0.47	106.14	1	7.81	14.05	1,225.59	408.42	0.33	0.60	1,773.07	5.32	0.54	78.88
VML165	1	6	5.56	9.96	875.74	502.45	0.57	0.28	1,038.16	4.31	0.59	115.34	1	8.76	14.23	1,570.97	532.22	0.33	0.57	1,587.79	5.92	0.58	87.10
VML041	1	2	5.55	10.89	811.34	393.39	0.48	0.32	1,110.24	3.51	0.53	112.23	1	8.26	15.36	1,324.70	431.45	0.32	0.63	1,301.75	4.62	0.59	89.50
VML179	2	2	5.10	10.81	653.02	410.17	0.64	0.33	1,127.82	3.60	0.53	114.05	1	7.74	16.52	1,249.61	431.45	0.34	0.71	1,516.41	4.73	0.55	88.28
VML009	1	7	5.15	8.58	712.05	505.24	0.72	0.23	1,028.38	4.18	0.59	120.96	6	9.14	13.38	1,303.67	483.27	0.38	0.52	1,231.95	4.91	0.60	93.36
VML087	1	10	4.78	11.03	760.36	468.89	0.62	0.30	992.66	4.48	0.61	106.17	1	8.27	17.76	1,676.08	584.05	0.35	0.72	1,668.47	6.08	0.57	91.90
VML004	2	3	5.18	13.74	870.38	508.04	0.59	0.36	992.40	4.70	0.60	107.97	3	9.35	20.56	2,099.55	555.25	0.28	0.78	1,590.81	6.76	0.59	82.44
VML042	1	3	5.15	12.52	843.54	432.54	0.51	0.34	1,094.55	5.20	0.61	85.07	4	8.58	19.38	1,631.04	512.07	0.32	0.79	1,530.05	7.39	0.62	75.01
VML113	1	2	5.03	14.30	704.00	385.00	0.55	0.38	1,086.70	3.97	0.56	97.07	3	8.95	22.91	1,643.05	419.93	0.27	0.84	1,425.79	5.31	0.58	80.25
VML046	1	10	5.12	11.50	773.77	536.00	0.69	0.31	1,136.90	4.94	0.60	107.18	10	9.32	15.82	1,895.33	716.49	0.37	0.62	1,794.82	8.50	0.61	84.19
*Mean of the top 20*			5.05	11.48	749.35	437.99	0.60	0.33	1,215.72	4.15	0.55	105.74		8.03	16.02	1,335.36	450.02	0.34	0.66	1,458.42	5.33	0.57	84.00
Bottom five																							
VML125	1	15	4.25	5.33	457.13	306.71	0.71	0.14	688.08	2.88	0.57	108.66	6	8.40	10.55	1,141.49	535.10	0.46	0.42	1,288.02	6.12	0.63	86.14
VML129	1	8	3.57	9.45	470.55	261.97	0.61	0.25	859.18	2.66	0.52	99.72	1	8.54	15.36	1,579.98	517.82	0.33	0.60	1,554.41	6.78	0.60	79.05
VML067	3	8	4.10	8.52	524.21	273.15	0.53	0.23	831.58	2.99	0.56	91.72	4	9.01	18.19	1,910.34	563.89	0.30	0.70	1,458.08	6.84	0.62	82.74
VML086	1	8	3.46	10.09	379.31	192.06	0.55	0.27	875.86	1.93	0.45	104.77	12	8.14	20.11	1,282.65	324.92	0.26	0.77	1,283.43	3.84	0.55	83.11
VML051	1	8	3.60	8.79	510.80	275.95	0.59	0.24	806.21	3.18	0.57	88.50	4	8.08	20.41	1,640.05	566.77	0.34	0.81	1,586.83	7.22	0.61	80.40
*Mean of the bottom 5*			3.80	8.44	468.40	261.97	0.60	0.23	812.18	2.73	0.54	98.67		8.43	16.92	1,510.90	501.70	0.34	0.66	1,434.15	6.16	0.60	82.29

^1/^SD, stalk diameter (mm); PH, plant height (cm); SDW, shoot dry weight (mg); RDW, root dry weight (mg); RSR, root to shoot dry weight ratio; DG, daily growth (cm); LRL, lateral root length (cm); RV, root volume (cm^3^); RAD, root average diameter (mm) and RTD, root tissue density (mg cm^-3^).

## 4 Discussion

Improved low-P tolerant and P-efficient maize varieties can be developed by enhancing P-acquisition from soil through root architecture and morphology modifications in maize breeding germplasm (Wang et al., 2010; Lynch, 2011; Zhang et al., 2013; Li et al., 2016; Liu et al., 2017a; Wen et al., 2017; Jia et al., 2018; Sun et al., 2018; Perkins and Lynch, 2021; Maqbool et al., 2022; Mathew and Shimelis, 2022). The strong genetic association among root traits and P-acquisition has also been confirmed by QTL and association mapping studies in maize under low-P stress conditions (Cai et al., 2012; Mendes et al., 2014; Azevedo et al., 2015; Gu et al., 2016; Xu et al., 2018; Wang et al., 2019). Therefore, the characterization of maize germplasm for seedling root traits under low P stress conditions can accelerate the development of high P-use efficient and P-tolerant varieties, mainly for tropical environments. In our study, we evaluated a panel of tropical maize inbred lines from UFV breeding program for root and shoot traits under optimal (AP) and low P stress (NAP) conditions. Even though P stress markedly affected the performance of inbred lines reducing their genotypic values for almost all traits, except for RSR and RTD, we found a huge genotypic variation among the inbred lines for all tested traits under both P conditions. In agreement with our results, several studies have reported the presence of genetic variability for shoot and root traits at seedling stage among maize inbred lines in response to low-P stress, and that maize inbred lines have responded negatively to low-P stress, except for RSR and RTD (Zhang et al., 2015; Wen et al., 2017; Almeida et al., 2018; Wang et al., 2019). The larger genotypic values of RSR under NAP than under AP might be due to a larger reduction of SDW compared to RDW and/or greater biomass allocation to root under low-P stress (Postma et al., 2014; Wang et al., 2015; Kramer-Walter and Laughlin, 2017; Lin et al., 2020). Moreover, the increased mean of RTD under NAP supports the theory that plants increase the allocation of biomass to their roots as a strategy to acclimate and adapt to nutrient limitation (Freschet et al., 2015; Van de Wiel et al., 2016; Liang et al., 2023). According to Bayuelo-Jiménez et al. (2011), maize genotypes with enhanced P-use efficiency had better maintenance of biomass allocation to roots under low-P stress than inefficient genotypes.

In any plant breeding program, the genetic gain is directly associated with both the presence of genetic variability in the breeding germplasm and the magnitude of $\hat{\text{h}}\frac{2}{\text{x}}$ values of a target trait (Falconer and MacKay, 1996; Hallauer et al., 2010; De La Fuente et al., 2013). In addition to huge genotypic variation observed in our inbred lines panel, the $\hat{\text{h}}\frac{2}{\text{x}}$ values were very high (>0.78) for most traits, even under low-P stress, and they were also larger than $\hat{\text{h}}\frac{2}{\text{x}}$ values reported in others studies with maize roots under low-P stress (Zhang et al., 2014; Almeida et al., 2018; Wang et al., 2019). Consequently, good genetic progress can be made by selecting inbred lines for root morphology under both optimal and low-P stress environments. Also, the traits RSR, LRL, TRL, RAD and RTD presented higher $\hat{\text{h}}\frac{2}{\text{x}}$ values under NAP than under AP, thus this set of root traits may positively impact the genetic improvement of tropical maize for P-efficiency and low-P tolerance across poor soils with P deficiency.

The existence of inbred lines × P conditions interaction associated with both low-to-intermediate $\hat{\text{h}}\frac{2}{\text{x}}$ values (<0.70) obtained across P conditions and low-to-moderate values of Spearman correlation estimates (<0.50) between NAP and AP indicated that the best inbred lines under NAP were not the best ones under AP condition (cross-over interaction; Yan et al., 2007). Thus, the selection of superior tropical maize inbred lines for root-related traits must be done under each P condition, and indirect selection of inbred lines for tolerance to low-P stress under AP will potentially lead to a low or no genetic gain under P stress environments (Zhang et al., 2015; Gu et al., 2016; Liang et al., 2023). Also, as our $\hat{\text{h}}\frac{2}{\text{x}}$ values under low-P stress were very high for almost all traits (most of them > 0.80), the improvement of tropical maize for root traits and P-use efficiency will provide great genetic gains across low-P environments. In agreement with our results, the presence of inbred lines × P conditions interaction has been reported in previous studies targeting the selection of low-P tolerant maize inbred lines (Zhang et al., 2014, 2015; Gu et al., 2016; Li et al., 2021; Liang et al., 2023). These authors also recommended that the selection of maize inbred lines adapted to low-P conditions must be done directly under low P condition. In our study, we observed that some inbred lines showed a drastic reduction in their performance when grown under NAP conditions, whereas other lines were less affected by low-P stress and consequently they had a little reduction in their phenotypic performance compared to the optimal P condition. According to Li et al. (2021), both sensitive and low-P tolerant inbred lines are interesting for inheritance studies such as diallel analysis and QTL mapping, whereas more low-P tolerant lines might be good candidates to improve maize for adaptation to low-P conditions across tropical environments in Brazil.

The P stress affected the magnitude of the genotypic correlations’ estimates, and almost all of them showed smaller values under NAP than under AP, particularly the estimates between root and shoot traits. Similarly, Zhang et al. (2014) also observed weaker associations between root and shoot traits under low-P stress compared with non-stress environments in a large set of maize inbred lines. However, we found positive and moderate-to-strong correlations (>0.60) among RDW and the shoot traits SD and SDW, and among RDW and the root traits, TRL, RSA and RV, under both P conditions. As RDW showed high $\hat{\text{h}}\frac{2}{\text{x}}$ values (0.84 and 0.86, at NAP and AP, respectively) and it is faster and cheaper to measure than root traits, it might be used for earlier selection of tropical maize inbred lines based on root size and shoot traits. Liu et al. (2017) proposed RDW as a very reliable indicator for genetic evaluation of maize root system architecture at early growth stages. In another study, Abdel-Ghani et al. (2013) suggested that RDW may accurately predict shoot and root traits in maize under both N stress and non-stress conditions. According to Yu et al. (2015), new maize cultivars with greater RDW are more resistant to stress and have higher yield. In addition to RDW, we can also employ SD and/or SDW in the evaluation and selection of tropical maize inbred lines for root size targeting optimal environments since both were strongly positively associated with most root traits under AP in our study. Also, the SD and SDW had high $\hat{\text{h}}\frac{2}{\text{x}}$ values (>0.75) under AP and they are easier, faster, and cheaper to phenotype than root traits. Root traits have been significantly impacted maize’s ability to acquire P from soil. Chen et al. (2022) found that maize varieties with deeper and more extensive root exhibited better P acquisition efficiency and enhanced grain yield under low-P environments. A strong root system before the tasseling period is an important factor in obtaining high maize yields. It has been reported that the RDW is positively correlated with leaf area, and the SDW is positively correlated with grain yield (Nkebiwe et al., 2016, Liu et al., 2017b, Cheng et al., 2020). The formation of more and deeper roots improves the plant’s ability to take up soil nutrients, enabling it to maintain a high biomass during the later growth stages, which enhances the grain yield and nutrient use efficiency. Also, it is noteworthy that the correlation results presented in our work accurately reflect the relationships between traits under differing P conditions due to the controlled greenhouse trial carried out in our research. In contrast, field conditions may exhibit significant variability in P availability due to soil heterogeneity and other environmental factors, which might potentially affect the accuracy and consistency of correlation results.

In relation to multi-trait selection strategy, the positive and moderate association between LPTI and LPPI indexes observed in our study was reported in maize evaluation for low-P tolerance by Zhang et al. (2015). This implies that most low-P tolerant inbred lines also had good performance under low-P environment. Consequently, forty-seven inbred lines from our inbred lines panel were classified into TG group. As our main proposal is to improve tropical maize for both low-P stress performance and tolerance, we selected a set of top 20 out of 47 TG lines, which will be used in the development of breeding populations, new inbred lines and, consequently, high-yielding and low-P tolerant hybrids of maize for tropical environments. Even though LPTI showed weak or no association with tested traits, the LPPI was strongly positively correlated with most of them, and we observed that most tropical maize inbred lines with good performance for SD, SDW, RDW and LRL under NAP were among the top 20 superior inbred lines. Thus, the simultaneous improvement for both low-P stress performance and tolerance appears to be feasible and could result in tropical maize inbred lines with greater root size, thicker stalk, and larger plant biomass across low-P environments. In contrast to low-P stress, the selection of maize inbred lines based on LPTI values will result in inbred lines with poor root and shoot performance under optimal P environments since LPTI associated negatively with almost all traits under AP, which agrees with the presence of cross-over inbred lines × P conditions interaction for all tested traits observed in our study.

The genetic diversity assessment in the breeding germplasm is mandatory and very useful to help maize breeders to guide the crosses in the development of heterotic hybrids and breeding populations, and, consequently, increase the rate of genetic gain in their breeding programs (Hallauer et al., 2010; Andorf et al., 2019; Swarup et al., 2021). Our results showed that tropical maize inbred lines from UFV breeding program are largely diverse for seedling shoot and root traits, irrespective of P conditions. Interestingly, inbred lines from different heterotic groups were grouped together in the same cluster indicating that there is a little genetic divergence between many pairs of heterotic lines for seedling root traits. In contrast, most tropical maize lines from the same heterotic group were clustered in different genetic diversity clusters suggesting that there is a huge genetic diversity within each heterotic group that can be explored in our breeding program for root traits. Likewise, Kumar et al. (2012) also found absence of concordance between the clustering of maize inbred lines based on seedling root traits and their genetic background. Nevertheless, our genetic diversity results can be combined with heterotic grouping information to assist us in the development of mapping and breeding populations - synthetic and/or bi-parental populations – from highly diverse and good performance inbred lines within each heterotic group. Moreover, we can develop high yielding and low-P stress tolerant maize hybrids setting up crosses between divergent and good root performance lines from different heterotic groups targeting low-P stress environments.

The top 20 inbred lines simultaneously selected for both low-P stress tolerance and performance were highly diverse, mainly under NAP, and allocated into two heterotic groups (ten from each group; Faria et al., 2022). Thus, we recommended crossing the ten lines from heterotic group I with the ten lines from group II using North Carolina Design mating design II (Comstock and Robinson, 1948) to generate 100 single-crosses hybrids. These hybrids will be evaluated for root traits, P-efficiency, low-P stress tolerance and agronomic traits under contrasting P conditions across several tropical environments in Brazil. Then, information on general and specific combining abilities among the 20 inbred lines might be very useful to improve our understating about the inheritance of both low-P tolerance and P-efficiency in tropical maize, and define breeding strategies for low-P stress environments. After that, we can select a small set (3-5) of outstanding hybrids to be grown across tropical environments and more diverse management conditions in Brazil, mainly under low-P conditions. In addition to hybrids, we propose to develop two synthetic populations from the top 20 inbred lines to be used as base populations to begin a reciprocal recurrent selection program for low-P tolerance: one derived from the recombination among the ten inbred lines from heterotic group I and another from the ten lines from heterotic group II (Kutka and Smith, 2007; Hallauer and Carena, 2012; Kolawole et al., 2019). Also, phenotypically divergent inbred lines within each heterotic group among the top 20 lines might be crossed in pairs to produce bi-parental populations that along with two synthetic populations must be used as source populations for developing new inbred lines of tropical maize targeting low-P stress environments (Hallauer and Carena, 2009; Guimarães et al., 2018).

In addition to the breeding proposals, we intend to combine association mapping and linkage analysis approaches to improve our understanding of the molecular mechanisms underlying low-P tolerance and P-use efficiency in tropical maize (Zhang et al., 2021; Farooqi et al., 2022; Shi et al., 2022). For linkage analysis, we suggest developing bi-parental mapping populations from crossing between superior inbred lines from TG group with the worst inbred lines from SP group that belong to different heterotic groups and were highly divergent for seedling root traits, mainly under NAP. Here, at least three QTL mapping populations may be developed from crossing between inbred lines VML121 and VML051, VML133 and VML086, and VML165 and VML175. Then, these populations might be genotyped and evaluated for seedling and adult root traits, low-P tolerance, and agronomic traits across tropical environments, mainly under low-P conditions. Moreover, the presence of huge genotypic variation and genetic diversity in our inbred lines panel associated with high $\hat{\text{h}}\frac{2}{\text{x}}$ values for most tested traits and fast linkage disequilibrium decay (Faria et al., 2022), suggests that our lines panel is suitable for association mapping studies targeting to uncover the genetic architecture of low-P tolerance and P-use efficiency in tropical maize (Wang et al., 2019; Deja-Muylle et al., 2020; Cortes et al., 2021; Ren et al., 2022; Zuffo et al., 2022). Thus, our next step is to genotype the maize inbred lines using genotyping-by-sequencing (GBS) approach and then to perform association mapping studies to identify candidate genes that affect root traits, low-P tolerance, and P-use efficiency in tropical maize. Finally, genomic regions and candidate genes revealed by linkage analysis and association mapping approaches may be successfully employed in the genetic improvement of tropical maize for low-P stress environments using marker-assisted selection and/or genomic prediction (Maharajan et al., 2018; Wang et al., 2019; Mathew and Shimelis, 2022; Liu et al., 2023).

In conclusion, the panel of tropical maize inbred lines from UFV breeding program has large genetic variability for root and shoot seedling traits under both AP and NAP conditions. Thus, we selected a set of promising inbred lines that have genetic potential to be used in the development of breeding populations and hybrids of tropical maize for low-P tolerance and P-efficiency to be grown across diverse Brazilian environments, mainly under low-P stress. Our inbred lines also showed large genetic diversity based on seedling root and shoot traits that can be useful to guide crosses to develop hybrids and populations, and to identify candidate genes associated with root and seedling traits by association mapping. The RDW might be successfully employed to select inbred lines for root size and shoot traits, and, consequently, improve tropical maize for low-P stress environments, whereas SD and SDW are reliable traits to be used in the genetic improvement of tropical maize for root traits under non-stresses environments. Finally, the selection of superior inbred lines for low-P tolerance, based on LPTI values, will result in tropical maize with good low-P performance for most root and shoot traits.

## Data Availability

The original contributions presented in the study are included in the article/**Supplementary Material**. Further inquiries can be directed to the corresponding author.
