# Supplementary material for: Genetic diversity, relationships among traits and selection of tropical maize inbred lines for low-P tolerance based on root and shoot traits at seedling stage
Source: Front Plant Sci. 2024 Oct 1;15:1429901. doi: 10.3389/fpls.2024.1429901 (PMC11473326; doi:10.3389/fpls.2024.1429901)
Supplement: Supplementary file 2 [file Table2.pdf]

Supplementary Material

Genetic diversity, relationships among traits and selection of tropical maize inbred lines for low-P tolerance based on root and shoot traits at seedling stage

Supplementary table 2. Low-phosphorus tolerance index (LPTI), low-phosphorus performance index (LPPI) and predicted genotypic means for root and shoot-related traits measured in 151 tropical maize inbred lines evaluated under non-applied and applied P.

			Applied phosphorus							
**Inbred Lines**	**LPTI**	**LPPI**	**SD**	**PH**	**SDW**	**RDW**	**RSR**	**TDW**	**DG**	**LRL**
VML001	1.318	0.999	7.81	14.05	1225.59	408.42	0.33	1625.20	0.60	1773.07
VML002	3.139	0.491	6.32	13.56	748.06	206.87	0.24	933.10	0.56	1055.94
VML003	0.454	0.98	8.40	21.23	1868.30	474.64	0.27	2341.48	0.83	1530.09
VML004	0.78	1.105	9.35	20.56	2099.55	555.25	0.28	2658.82	0.78	1590.81
VML005	0.367	0.071	8.23	18.65	1688.10	483.27	0.29	2169.21	0.72	1489.07
VML006	-0.52	-1.09	9.51	20.47	2646.16	601.32	0.26	3257.23	0.72	1587.43
VML007	-0.26	0.835	9.69	16.15	1904.34	523.58	0.28	2429.12	0.62	1712.68
VML008	-0.45	0.311	9.22	18.16	2018.46	641.63	0.32	2667.88	0.66	1430.99
VML009	0.995	0.628	9.14	13.38	1303.67	483.27	0.38	1782.36	0.52	1231.95
VML010	-1.22	-0.78	10.58	18.74	2036.48	632.99	0.32	2676.95	0.69	1582.58
VML011	-0.32	0.246	10.28	18.31	1739.15	575.41	0.33	2317.30	0.70	1609.26
VML012	-1.54	-0.55	9.14	21.54	2000.44	609.96	0.31	2616.51	0.80	1639.32
VML015	-1.16	-0.84	10.85	24.10	2462.95	638.75	0.28	3112.16	0.82	1468.38
VML016	-1.32	0.534	10.42	21.57	2772.29	753.92	0.29	3544.34	0.73	2136.32
VML017	-0.9	-0.78	8.97	18.43	2315.79	768.31	0.34	3100.07	0.67	1480.94
VML018	0.305	-0.14	7.87	14.99	1144.50	396.90	0.34	1531.51	0.66	1552.48
VML020	0.705	-0.38	7.35	17.49	1315.69	417.05	0.32	1724.93	0.71	1038.39
VML021	-1.02	-0.77	8.70	15.88	1558.96	555.25	0.35	2114.81	0.62	1737.83
VML022	-1.28	0.266	9.56	20.35	2919.46	679.06	0.27	3613.86	0.72	1708.57
VML023	-0.33	0.331	10.49	18.22	1582.98	535.10	0.34	2117.83	0.69	1562.84
VML024	-0.81	0.381	8.70	19.41	2105.56	722.25	0.34	2840.15	0.70	1535.86
VML025	-0.54	-1.4	7.52	19.35	1372.75	428.57	0.32	1794.45	0.78	1263.61
VML026	0.016	0.245	8.29	18.34	1408.79	457.36	0.33	1860.94	0.73	1650.38
VML027	-0.18	0.545	10.62	20.32	1997.44	598.44	0.31	2601.39	0.78	1905.91
VML028	0.908	-0.5	7.25	16.91	1294.66	356.59	0.28	1640.31	0.65	1381.36
VML030	1.539	1.299	8.23	15.36	1483.87	535.10	0.36	2018.09	0.67	1533.63
VML031	0.122	0.29	7.96	19.19	1429.81	379.62	0.27	1800.49	0.80	1667.77
VML032	-0.23	0.821	8.98	21.81	1964.40	604.20	0.31	2574.19	0.85	1876.01
VML033	0.093	0.888	9.72	22.45	2441.93	661.78	0.29	3115.18	0.89	1655.02
VML034	-0.41	0.218	9.23	18.95	1748.16	687.70	0.38	2444.24	0.72	1613.37
VML036	1.682	1.457	7.63	13.96	1225.59	434.33	0.35	1652.40	0.62	1657.50
VML039	-0.89	-0.22	11.19	18.04	2117.57	687.70	0.33	2815.98	0.60	1307.28

VML040	-0.28	0.552	8.02	19.53	1564.96	572.53	0.36	2138.99	0.80	1861.99
VML041	1.209	0.901	8.26	15.36	1324.70	431.45	0.32	1749.11	0.63	1301.75
VML042	0.699	0.695	8.58	19.38	1631.04	512.07	0.32	2142.01	0.79	1530.05
VML043	-0.85	-1	8.31	17.34	1387.77	437.21	0.31	1818.62	0.74	1673.05
VML044	0.747	0.388	8.34	14.90	1291.66	466.00	0.36	1752.13	0.60	1607.29
VML046	0.646	1.024	9.32	15.82	1895.33	716.49	0.37	2622.55	0.62	1794.82
VML047	0.466	-0.28	7.35	16.06	1192.55	396.90	0.33	1579.86	0.63	1437.38
VML048	-0.76	-0.93	8.03	15.02	1069.42	399.78	0.37	1458.97	0.60	1525.29
VML049	-0.05	-0.21	8.11	15.18	1144.50	419.93	0.36	1555.69	0.62	1417.49
VML050	-0.29	-1	8.33	18.83	1444.83	489.03	0.33	1930.45	0.76	1685.29
VML051	-1.4	-1.41	8.08	20.41	1640.05	566.77	0.34	2208.50	0.81	1586.83
VML052	-0.67	0.03	9.14	21.81	2856.39	722.25	0.28	3595.72	0.76	1453.60
VML053	0.453	0.206	8.71	17.09	1637.04	561.01	0.34	2199.43	0.65	1375.34
VML054	-1.34	-0.32	10.25	19.59	2640.15	647.39	0.26	3299.54	0.73	1926.62
VML055	-0.33	0.144	8.73	20.99	2087.54	454.48	0.24	2540.95	0.80	1750.12
VML056	0.086	-0.27	7.71	18.71	1480.87	572.53	0.38	2054.36	0.73	1423.66
VML057	0.453	-0.02	8.55	17.67	1225.59	437.21	0.35	1655.42	0.72	1267.29
VML058	-0.51	-0.74	9.40	20.23	2156.62	569.65	0.28	2731.35	0.73	1544.26
VML059	0.079	0.336	9.03	16.67	1820.24	517.82	0.29	2338.46	0.62	1598.95
VML061	-0.02	-1.6	7.26	19.19	1141.49	376.74	0.33	1507.33	0.77	1448.02
VML063	0.066	0.272	8.85	17.16	1856.28	532.22	0.30	2389.83	0.67	1475.62
VML064	0.598	0.253	8.18	14.66	1282.65	405.54	0.31	1679.60	0.62	1447.00
VML065	0.575	-0.42	7.71	15.42	1282.65	417.05	0.33	1691.69	0.65	1440.22
VML066	-0.65	0.026	8.97	19.07	1820.24	612.84	0.33	2438.19	0.75	1740.60
VML067	-1.26	-1.13	9.01	18.19	1910.34	563.89	0.30	2477.48	0.70	1458.08
VML068	-0.17	0.543	9.11	19.07	2102.56	684.82	0.33	2797.84	0.73	1761.12
VML069	1.064	-0.11	6.67	16.76	979.32	322.04	0.32	1286.70	0.72	1281.73
VML070	0.279	0.244	8.88	18.13	1607.01	509.19	0.32	2114.81	0.69	1472.68
VML073	-1.27	-0.25	10.14	16.94	1907.34	578.29	0.31	2489.57	0.64	1635.81
VML074	1.423	-0.03	7.18	17.67	1057.40	322.04	0.30	1365.28	0.73	1191.48
VML075	0.671	-0.22	7.32	16.42	1144.50	471.76	0.39	1610.09	0.69	1523.18
VML076	0.036	1.351	9.01	22.33	1877.31	532.22	0.30	2410.99	0.88	1765.41
VML080	0.546	0.259	8.40	15.94	1628.03	503.43	0.31	2129.92	0.64	1745.36
VML081	0.108	-0.69	7.84	16.76	1144.50	466.00	0.40	1604.04	0.69	1238.39
VML083	0.305	-0.83	6.61	16.39	895.22	362.35	0.39	1244.39	0.69	1122.68
VML084	0.16	-0.58	7.47	14.75	1288.66	454.48	0.35	1737.02	0.59	1259.82
VML085	-0.65	-0.05	9.94	21.29	1988.43	480.40	0.26	2468.41	0.78	1618.91
VML086	-1.29	-1.22	8.14	20.11	1282.65	324.92	0.26	1594.98	0.77	1283.43
VML087	0.843	0.446	8.27	17.76	1676.08	584.05	0.35	2262.90	0.72	1668.47
VML088	1.55	-0.1	7.68	15.27	1417.80	402.66	0.29	1812.58	0.60	1098.36
VML089	0.627	-0.34	8.18	19.89	1931.37	440.09	0.25	2368.68	0.77	1545.16
VML090	-1	-0.75	11.01	18.86	2426.91	641.63	0.28	3078.91	0.66	1494.75
VML091	0.302	-0	7.13	16.52	1276.64	474.64	0.36	1746.09	0.68	1634.96
VML092	-0.06	0.272	9.01	18.46	1811.23	471.76	0.28	2281.03	0.73	1862.53

VML093	-0.48	-0.25	9.38	18.01	1823.25	503.43	0.29	2326.37	0.72	1673.06
VML094	-1.03	-1.08	8.45	17.43	1561.96	517.82	0.33	2078.54	0.67	1479.06
VML095	-0.51	-0.7	8.76	19.68	1540.94	480.40	0.31	2018.09	0.74	1412.93
VML096	-0.53	-0.41	8.33	15.21	1534.93	546.62	0.35	2081.56	0.60	1719.22
VML097	-0.11	-0.04	8.63	18.13	1820.24	609.96	0.33	2435.17	0.71	1717.69
VML099	1.124	0.208	8.06	15.33	1342.72	434.33	0.32	1770.27	0.63	1499.93
VML100	-0.54	0.134	9.96	20.26	2898.43	736.64	0.27	3653.15	0.74	1475.94
VML102	-0.86	-0.01	9.77	22.09	2441.93	632.99	0.27	3084.96	0.78	1473.75
VML104	-1.15	-0.78	9.20	22.21	2018.46	529.34	0.27	2550.02	0.81	1573.76
VML106	-0.79	0.665	10.49	20.38	2318.79	756.80	0.33	3091.00	0.71	1754.29
VML107	-0.9	0.014	9.97	18.13	2054.50	644.51	0.32	2707.17	0.66	1740.69
VML110	-1.29	-0.52	9.71	20.32	2027.47	699.21	0.34	2737.40	0.70	1779.48
VML111	-0.81	-0.81	8.05	14.20	1327.70	509.19	0.38	1833.74	0.57	1443.44
VML112	0.443	0.459	9.08	15.57	1682.09	578.29	0.35	2262.90	0.61	1648.22
VML113	0.659	0.781	8.95	22.91	1643.05	419.93	0.27	2057.38	0.84	1425.79
VML114	0.53	0.901	9.42	19.07	1700.11	552.37	0.32	2253.83	0.75	1628.74
VML116	-0.67	-1.08	9.00	15.72	1540.94	463.12	0.31	1999.96	0.60	1441.12
VML117	-1.23	-0.3	10.59	20.96	1913.35	601.32	0.32	2519.79	0.77	1855.78
VML118	-0.03	1.294	10.51	16.27	2198.66	######	0.45	3236.07	0.61	1635.79
VML119	1.596	0.995	7.31	15.72	937.27	454.48	0.48	1383.42	0.65	1544.46
VML120	0.595	-0.55	8.00	16.61	1297.67	370.99	0.29	1658.44	0.64	1216.11
VML121	1.634	1.355	7.83	17.95	1324.70	417.05	0.31	1734.00	0.75	1386.64
VML122	-0.24	-0.56	8.24	18.65	1558.96	460.24	0.30	2015.07	0.72	1664.95
VML123	-0.13	0.668	10.11	19.59	2309.78	684.82	0.31	3006.38	0.69	1687.42
VML124	-0.07	-0.78	6.76	14.11	1000.34	376.74	0.36	1365.28	0.56	1339.30
VML125	-0.97	-1.23	8.40	10.55	1141.49	535.10	0.46	1673.56	0.42	1288.02
VML126	-0.07	0.357	8.48	20.44	2171.63	561.01	0.28	2737.40	0.73	1633.49
VML127	-0.99	-0.97	8.12	19.19	1715.13	578.29	0.34	2296.14	0.74	1437.72
VML128	0.235	0.289	8.15	16.24	1375.75	454.48	0.33	1824.67	0.66	1270.62
VML129	-1.02	-1.17	8.54	15.36	1579.98	517.82	0.33	2096.67	0.60	1554.41
VML130	1.402	0.427	6.95	15.97	1129.48	373.87	0.33	1492.22	0.71	1296.40
VML131	0.273	0.326	9.05	16.03	1979.42	644.51	0.35	2631.62	0.60	2019.74
VML132	-1.29	-1.06	8.66	20.63	1763.18	537.98	0.31	2302.19	0.74	1417.51
VML133	1.505	0.57	7.55	12.07	1048.39	460.24	0.43	1501.29	0.52	1367.10
VML134	-1.4	0.382	10.83	26.26	2916.45	788.47	0.28	3725.68	0.88	2076.85
VML136	0.439	0.263	8.66	16.91	1736.15	561.01	0.32	2299.17	0.64	1083.43
VML137	1.737	0.936	7.86	13.56	985.32	362.35	0.36	1335.06	0.57	1319.05
VML138	-1.14	-0.54	9.16	18.62	1955.39	549.50	0.30	2507.70	0.71	2080.77
VML139	-0.13	0.237	9.96	17.40	1631.04	486.15	0.30	2114.81	0.69	1281.81
VML140	-0.63	0.396	10.33	20.72	3117.68	941.06	0.32	4088.35	0.73	1919.00
VML141	-0.86	-0.18	9.16	21.96	2177.64	483.27	0.24	2661.84	0.84	1900.67
VML143	-0.05	0.435	9.35	16.76	1522.92	529.34	0.35	2051.34	0.67	1484.68
VML144	-1.31	1.153	10.82	26.35	3078.63	926.67	0.31	4033.95	0.96	2061.59
VML145	0.831	-0.02	8.34	15.30	1309.68	391.14	0.30	1691.69	0.60	1272.99

VML146	0.578	0.296	8.89	18.56	1456.84	471.76	0.32	1924.40	0.72	1522.26
VML147	-0.39	-0.09	8.30	23.55	1937.37	483.27	0.26	2420.06	0.91	1672.95
VML148	0.696	0.343	8.02	17.55	1525.92	399.78	0.27	1918.36	0.67	1225.55
VML150	0.07	0.265	8.52	17.98	1679.09	517.82	0.32	2196.41	0.65	1532.79
VML151	-0.13	0.669	9.40	17.46	1973.41	552.37	0.29	2528.86	0.65	1635.83
VML152	2.076	-0.3	5.96	16.46	805.12	221.27	0.26	1005.63	0.67	1150.58
VML153	2.37	0.595	6.78	15.18	946.28	284.61	0.30	1214.17	0.68	1262.95
VML154	-1.32	0.225	10.92	23.79	2736.25	725.13	0.28	3477.85	0.83	1656.68
VML155	-0.3	0.866	8.49	22.33	1802.22	563.89	0.32	2368.68	0.88	1536.51
VML156	0.483	-0.65	8.26	14.75	1006.35	408.42	0.39	1404.57	0.65	1567.31
VML157	0.646	0.425	7.75	15.39	1342.72	489.03	0.36	1827.69	0.67	1589.20
VML158	2.696	-0.44	5.59	14.84	691.00	218.39	0.30	887.76	0.67	961.85
VML159	0.18	0.348	10.27	15.27	1724.14	523.58	0.31	2247.79	0.57	1464.12
VML160	0.082	0.186	8.46	18.07	1790.21	543.74	0.31	2335.43	0.72	1556.65
VML161	0.135	-0.2	8.33	22.15	1928.36	566.77	0.30	2498.64	0.75	1399.39
VML162	-0.83	-1.28	9.42	17.79	1745.16	431.45	0.26	2172.23	0.66	1616.51
VML164	0.011	-0.15	8.26	20.41	1829.25	497.67	0.28	2326.37	0.75	1417.81
VML165	1.223	0.95	8.76	14.23	1570.97	532.22	0.33	2102.72	0.57	1587.79
VML168	0.02	-0.17	8.97	19.99	1697.11	483.27	0.30	2178.28	0.76	1367.23
VML170	-0.41	-0.72	8.30	18.16	1141.49	448.72	0.38	1582.89	0.72	1275.99
VML171	0.629	-0.27	8.95	12.95	1105.46	445.85	0.39	1543.60	0.56	1479.87
VML173	-1.17	-0.24	10.46	20.75	2339.82	814.38	0.35	3172.60	0.72	1776.94
VML174	1.345	0.882	7.94	17.03	1258.62	396.90	0.31	1646.35	0.72	1323.89
VML175	-1.45	-1.05	8.88	21.36	2009.45	491.91	0.26	2501.66	0.82	1560.62
VML176	0.047	0.289	9.74	16.15	2108.56	664.66	0.32	2782.73	0.60	1536.62
VML178	-1.39	-0.03	10.45	20.96	2381.86	710.73	0.31	3106.11	0.78	1387.08
VML179	1.051	0.801	7.74	16.52	1249.61	431.45	0.34	1673.56	0.71	1516.41
VML180	0.117	-0.59	7.86	16.06	1513.91	454.48	0.31	1963.69	0.65	1375.47
VML183	0.817	0.349	7.23	17.73	1273.64	414.17	0.32	1679.60	0.75	1559.90
VML184	1.045	-0.33	7.23	17.98	1330.70	385.38	0.30	1706.80	0.74	1516.20
VML188	-0.94	-0.16	8.85	21.14	2646.16	733.76	0.29	3396.25	0.78	1561.00

			Applied phosphorus					Non-applied phosphorus		
**Inbred Lines**	**LPTI**	**LPPI**	**TRL**	**RSA**	**RV**	**RAD**	**RTD**	**SD**	**PH**	**SDW**
VML001	1.318	0.999	2494.10	410.24	5.32	0.54	78.88	4.47	11.66	717.42
VML002	3.139	0.491	1398.90	189.74	2.26	0.49	82.33	4.56	11.96	540.31
VML003	0.454	0.98	2249.40	413.83	6.13	0.59	79.70	4.62	13.29	741.57
VML004	0.78	1.105	2421.45	454.09	6.76	0.59	82.44	5.18	13.74	870.38
VML005	0.367	0.071	2188.81	392.80	5.66	0.58	85.08	4.56	12.97	763.04
VML006	-0.52	-1.09	2414.23	449.87	6.64	0.59	88.01	3.96	10.57	585.93
VML007	-0.26	0.835	2591.47	495.10	7.45	0.60	74.61	5.14	9.43	779.14
VML008	-0.45	0.311	2296.43	513.26	9.27	0.69	75.08	4.73	11.05	746.94
VML009	0.995	0.628	1790.52	325.29	4.91	0.60	93.36	5.15	8.58	712.05
VML010	-1.22	-0.78	2389.48	459.94	7.06	0.61	87.12	4.29	10.17	575.20
VML011	-0.32	0.246	2447.60	466.78	7.08	0.60	82.62	4.71	11.32	631.55

VML012	-1.54	-0.55	2534.86	483.67	7.30	0.60	83.29	4.03	10.39	551.05
VML015	-1.16	-0.84	2312.28	474.35	7.81	0.64	82.56	3.94	11.42	583.25
VML016	-1.32	0.534	3209.14	585.62	8.20	0.57	88.71	4.60	11.05	695.95
VML017	-0.9	-0.78	2425.24	525.58	9.10	0.66	84.37	4.14	9.08	615.45
VML018	0.305	-0.14	2214.57	373.20	5.07	0.56	79.48	4.17	10.25	569.83
VML020	0.705	-0.38	1596.73	305.22	5.01	0.65	82.90	4.33	11.21	618.13
VML021	-1.02	-0.77	2490.88	431.93	5.94	0.56	89.85	4.17	8.76	529.58
VML022	-1.28	0.266	2761.60	597.59	10.26	0.66	77.43	4.49	10.68	714.74
VML023	-0.33	0.331	2310.12	419.24	6.09	0.59	86.04	4.62	11.08	679.85
VML024	-0.81	0.381	2405.84	496.46	8.22	0.65	86.23	4.72	10.44	875.74
VML025	-0.54	-1.4	1899.91	351.23	5.34	0.61	80.91	3.73	9.64	492.01
VML026	0.016	0.245	2410.34	410.57	5.54	0.55	82.61	4.45	10.87	671.80
VML027	-0.18	0.545	2840.68	526.17	7.62	0.58	80.50	4.97	12.54	679.85
VML028	0.908	-0.5	2102.34	370.63	5.37	0.59	72.32	4.24	12.76	669.12
VML030	1.539	1.299	2258.22	409.13	5.96	0.59	87.34	5.45	11.72	899.90
VML031	0.122	0.29	2376.41	390.25	5.07	0.54	78.50	4.42	11.69	669.12
VML032	-0.23	0.821	2826.30	535.56	7.93	0.59	78.89	4.57	13.31	730.84
VML033	0.093	0.888	2633.25	533.76	8.51	0.63	80.26	5.20	13.90	1001.87
VML034	-0.41	0.218	2626.15	531.14	8.48	0.63	82.13	4.46	11.77	776.46
VML036	1.682	1.457	2405.34	411.94	5.59	0.55	79.66	4.77	12.01	763.04
VML039	-0.89	-0.22	2236.77	485.63	8.56	0.67	81.87	4.47	11.00	642.29
VML040	-0.28	0.552	2842.65	529.82	7.75	0.59	77.29	4.75	11.16	639.60
VML041	1.209	0.901	1846.01	321.04	4.62	0.59	89.50	5.55	10.89	811.34
VML042	0.699	0.695	2354.33	466.39	7.39	0.62	75.01	5.15	12.52	843.54
VML043	-0.85	-1	2443.94	423.54	5.82	0.56	78.66	3.62	9.80	467.86
VML044	0.747	0.388	2383.65	417.35	5.82	0.56	81.18	4.51	11.11	604.72
VML046	0.646	1.024	2788.47	550.47	8.50	0.61	84.19	5.12	11.50	773.77
VML047	0.466	-0.28	2118.44	374.27	5.37	0.58	76.48	3.83	8.66	516.16
VML048	-0.76	-0.93	2152.34	354.65	4.69	0.54	83.92	3.87	8.95	408.83
VML049	-0.05	-0.21	2026.52	350.20	4.90	0.57	84.44	4.70	8.52	612.77
VML050	-0.29	-1	2516.12	463.20	6.73	0.58	76.32	3.71	9.32	689.25
VML051	-1.4	-1.41	2440.70	471.08	7.22	0.61	80.40	3.60	8.79	510.80
VML052	-0.67	0.03	2259.00	486.03	8.43	0.66	84.59	4.19	15.28	867.69
VML053	0.453	0.206	2187.14	443.93	7.29	0.64	79.46	4.81	12.41	738.89
VML054	-1.34	-0.32	3037.31	617.95	9.86	0.62	73.05	4.05	9.83	669.12
VML055	-0.33	0.144	2458.08	395.76	5.02	0.53	87.73	4.28	12.38	827.44
VML056	0.086	-0.27	2162.64	403.95	6.09	0.60	90.57	4.04	11.82	588.62
VML057	0.453	-0.02	1911.86	351.17	5.30	0.60	82.46	4.71	10.31	580.57
VML058	-0.51	-0.74	2350.03	440.03	6.63	0.60	84.76	4.10	11.29	744.26
VML059	0.079	0.336	2381.41	438.99	6.43	0.59	81.74	4.40	10.60	800.61
VML061	-0.02	-1.6	2075.89	351.82	4.83	0.56	79.07	3.67	8.87	430.29
VML063	0.066	0.272	2157.47	399.16	5.94	0.59	86.91	5.05	9.80	717.42
VML064	0.598	0.253	2122.09	380.35	5.54	0.58	76.83	4.65	9.88	693.27
VML065	0.575	-0.42	2025.27	337.62	4.56	0.55	88.28	4.04	9.88	556.42

VML066	-0.65	0.026	2717.47	534.04	8.24	0.61	78.47	4.50	10.12	636.92
VML067	-1.26	-1.13	2222.68	434.38	6.84	0.62	82.74	4.10	8.52	524.21
VML068	-0.17	0.543	2619.14	499.01	7.45	0.59	88.24	4.86	11.16	717.42
VML069	1.064	-0.11	1792.19	279.50	3.61	0.53	85.45	4.21	11.18	513.48
VML070	0.279	0.244	2174.40	385.18	5.50	0.58	88.62	4.82	12.54	602.03
VML073	-1.27	-0.25	2415.10	446.77	6.63	0.59	85.58	4.55	9.08	607.40
VML074	1.423	-0.03	1792.04	312.35	4.57	0.59	74.13	4.44	13.55	631.55
VML075	0.671	-0.22	2221.12	379.22	5.19	0.56	87.11	3.93	9.51	534.95
VML076	0.036	1.351	2573.88	453.29	6.29	0.56	83.91	5.12	12.28	859.64
VML080	0.546	0.259	2728.31	515.39	7.61	0.59	72.74	4.91	11.16	677.17
VML081	0.108	-0.69	1891.71	362.47	5.84	0.63	82.03	4.05	9.80	575.20
VML083	0.305	-0.83	1630.59	277.32	4.01	0.58	86.82	4.05	9.24	567.15
VML084	0.16	-0.58	1949.82	386.78	6.30	0.64	76.08	4.24	8.87	596.67
VML085	-0.65	-0.05	2425.99	451.15	6.67	0.59	75.81	4.77	11.80	765.72
VML086	-1.29	-1.22	1807.01	290.17	3.84	0.55	83.11	3.46	10.09	379.31
VML087	0.843	0.446	2437.96	431.69	6.08	0.57	91.90	4.78	11.03	760.36
VML088	1.55	-0.1	1686.63	315.86	5.00	0.63	81.42	4.63	11.29	787.19
VML089	0.627	-0.34	2331.53	413.76	5.83	0.57	78.12	4.38	12.70	669.12
VML090	-1	-0.75	2274.61	443.71	6.95	0.62	89.08	4.55	10.89	620.82
VML091	0.302	-0	2422.05	431.40	6.07	0.57	78.91	4.20	10.57	610.08
VML092	-0.06	0.272	2725.03	470.53	6.35	0.55	77.59	4.91	11.58	760.36
VML093	-0.48	-0.25	2491.26	472.10	7.06	0.59	76.02	4.42	9.56	615.45
VML094	-1.03	-1.08	2222.88	407.76	6.00	0.59	85.08	3.86	10.04	494.70
VML095	-0.51	-0.7	2122.73	384.04	5.64	0.59	84.59	3.94	10.65	567.15
VML096	-0.53	-0.41	2598.90	473.51	6.79	0.58	81.65	4.20	8.82	518.85
VML097	-0.11	-0.04	2585.66	482.08	7.07	0.58	85.29	4.54	10.65	738.89
VML099	1.124	0.208	2202.05	381.77	5.30	0.56	81.76	5.21	9.83	832.81
VML100	-0.54	0.134	2304.22	465.18	7.51	0.63	92.41	4.99	11.40	856.96
VML102	-0.86	-0.01	2359.30	479.83	7.83	0.63	83.56	4.54	11.98	618.13
VML104	-1.15	-0.78	2326.20	421.92	6.11	0.58	85.24	3.96	10.57	508.11
VML106	-0.79	0.665	2761.14	540.72	8.28	0.61	88.10	4.29	11.26	712.05
VML107	-0.9	0.014	2581.20	478.22	6.98	0.59	88.59	4.17	10.60	604.72
VML110	-1.29	-0.52	2782.60	532.67	8.00	0.60	85.90	3.81	10.31	521.53
VML111	-0.81	-0.81	2158.69	413.66	6.46	0.61	81.08	3.88	7.96	467.86
VML112	0.443	0.459	2423.68	438.74	6.30	0.58	88.52	5.23	9.48	797.93
VML113	0.659	0.781	2080.38	369.54	5.31	0.58	80.25	5.03	14.30	704.00
VML114	0.53	0.901	2536.70	511.77	8.15	0.62	73.73	5.35	11.29	720.11
VML116	-0.67	-1.08	2071.46	366.95	5.29	0.58	86.20	3.68	8.60	465.18
VML117	-1.23	-0.3	2816.55	534.72	7.90	0.59	78.85	4.47	12.41	593.98
VML118	-0.03	1.294	2614.49	578.74	10.16	0.67	93.41	5.47	10.84	945.51
VML119	1.596	0.995	2203.94	366.35	4.88	0.55	89.29	4.62	11.03	626.18
VML120	0.595	-0.55	1858.38	341.05	5.24	0.60	75.91	4.21	12.09	615.45
VML121	1.634	1.355	2021.24	361.25	5.26	0.59	80.40	5.61	12.36	956.25
VML122	-0.24	-0.56	2378.40	397.21	5.31	0.55	84.89	3.92	11.69	483.96

VML123	-0.13	0.668	2558.85	486.98	7.30	0.60	89.84	4.83	13.55	867.69
VML124	-0.07	-0.78	1961.70	323.91	4.37	0.55	84.46	3.65	9.11	398.09
VML125	-0.97	-1.23	1918.73	377.40	6.12	0.63	86.14	4.25	5.33	457.13
VML126	-0.07	0.357	2424.01	429.82	6.02	0.57	89.19	4.81	12.30	881.11
VML127	-0.99	-0.97	2266.13	465.62	7.72	0.64	78.17	3.86	11.16	521.53
VML128	0.235	0.289	1864.65	335.52	4.98	0.59	88.29	4.30	11.85	671.80
VML129	-1.02	-1.17	2347.29	446.57	6.78	0.60	79.05	3.57	9.45	470.55
VML130	1.402	0.427	1896.20	329.21	4.69	0.58	80.06	4.35	11.45	545.68
VML131	0.273	0.326	3043.21	577.50	8.45	0.58	79.13	4.45	10.81	588.62
VML132	-1.29	-1.06	2078.61	394.71	6.08	0.61	86.31	3.76	10.25	537.63
VML133	1.505	0.57	2097.69	394.64	6.02	0.61	78.83	4.72	9.06	642.29
VML134	-1.4	0.382	3079.35	571.95	8.19	0.58	91.61	4.81	12.01	674.49
VML136	0.439	0.263	1730.42	372.10	6.79	0.70	82.88	4.76	9.69	811.34
VML137	1.737	0.936	1876.74	306.30	4.11	0.55	87.41	5.51	9.93	768.41
VML138	-1.14	-0.54	3050.65	525.62	6.97	0.55	80.85	4.05	9.19	577.88
VML139	-0.13	0.237	1894.84	364.00	5.76	0.62	83.82	5.00	11.48	851.59
VML140	-0.63	0.396	3279.28	718.97	12.13	0.65	80.74	4.75	13.61	693.27
VML141	-0.86	-0.18	2683.21	440.17	5.65	0.53	83.82	4.17	11.96	722.79
VML143	-0.05	0.435	2285.41	448.90	7.11	0.63	77.69	5.08	10.71	757.67
VML144	-1.31	1.153	3272.06	686.29	11.05	0.63	84.16	4.67	13.34	814.03
VML145	0.831	-0.02	1855.51	319.26	4.53	0.58	84.59	4.88	11.85	722.79
VML146	0.578	0.296	2292.63	401.66	5.62	0.57	83.39	4.36	13.85	695.95
VML147	-0.39	-0.09	2442.65	427.46	5.93	0.56	82.09	4.66	12.70	647.65
VML148	0.696	0.343	1825.21	337.57	5.21	0.62	78.42	4.55	12.81	693.27
VML150	0.07	0.265	2230.38	405.80	5.97	0.59	85.98	4.42	11.18	585.93
VML151	-0.13	0.669	2480.65	479.87	7.34	0.61	78.47	5.23	9.69	779.14
VML152	2.076	-0.3	1554.30	226.30	2.81	0.51	79.72	4.14	12.89	526.90
VML153	2.37	0.595	1772.29	268.04	3.40	0.52	82.32	4.99	13.16	744.26
VML154	-1.32	0.225	2463.51	493.12	7.86	0.62	89.21	4.72	12.14	604.72
VML155	-0.3	0.866	2258.28	417.82	6.25	0.58	91.29	4.82	13.18	771.09
VML156	0.483	-0.65	2233.26	369.23	4.87	0.54	83.02	4.21	10.71	561.78
VML157	0.646	0.425	2347.79	434.73	6.45	0.59	79.50	4.61	10.01	628.87
VML158	2.696	-0.44	1343.05	203.81	2.73	0.55	79.10	4.35	12.25	567.15
VML159	0.18	0.348	2185.76	408.06	6.16	0.60	84.17	5.19	11.40	795.24
VML160	0.082	0.186	2406.28	472.76	7.41	0.61	77.61	4.86	10.28	830.13
VML161	0.135	-0.2	2027.13	370.94	5.53	0.59	94.63	4.49	12.17	690.59
VML162	-0.83	-1.28	2384.65	421.73	5.91	0.57	76.49	3.99	8.95	478.60
VML164	0.011	-0.15	2155.17	410.09	6.29	0.61	80.52	4.39	11.53	669.12
VML165	1.223	0.95	2339.90	416.34	5.92	0.58	87.10	5.56	9.96	875.74
VML168	0.02	-0.17	1972.76	362.01	5.45	0.60	86.83	4.66	12.41	569.83
VML170	-0.41	-0.72	1907.91	366.16	5.81	0.62	78.72	3.46	12.22	567.15
VML171	0.629	-0.27	2163.75	383.96	5.53	0.58	80.71	4.79	9.40	618.13
VML173	-1.17	-0.24	2785.36	582.60	9.56	0.63	84.46	4.05	11.29	607.40
VML174	1.345	0.882	1913.76	339.20	4.95	0.58	81.49	5.24	11.88	779.14

VML175	-1.45	-1.05	2280.69	402.62	5.70	0.57	84.82	3.75	10.79	604.72
VML176	0.047	0.289	2471.69	515.80	8.58	0.65	80.10	4.68	12.28	862.33
VML178	-1.39	-0.03	2163.26	454.69	7.80	0.66	88.03	4.49	11.11	733.52
VML179	1.051	0.801	2142.64	352.36	4.73	0.55	88.28	5.10	10.81	653.02
VML180	0.117	-0.59	1989.09	358.59	5.27	0.59	84.96	4.61	9.11	682.54
VML183	0.817	0.349	2286.88	399.17	5.55	0.57	79.10	4.31	12.38	666.44
VML184	1.045	-0.33	2186.49	376.04	5.18	0.56	77.05	4.23	10.41	634.24
VML188	-0.94	-0.16	2488.81	510.39	8.31	0.63	86.65	4.98	10.31	838.18

			Non-applied phosphorus							
**Inbred Lines**	**LPTI**	**LPPI**	**RDW**	**RSR**	**TDW**	**DG**	**LRL**	**TRL**	**RSA**	**RV**
VML001	1.318	0.999	415.76	0.58	1130.56	0.36	1647.33	2229.32	330.76	3.91
VML002	3.139	0.491	303.91	0.59	847.00	0.35	1215.18	1681.53	238.49	2.70
VML003	0.454	0.98	440.93	0.60	1178.72	0.38	1396.46	1991.23	331.88	4.41
VML004	0.78	1.105	508.04	0.59	1371.32	0.36	992.40	1617.17	307.23	4.70
VML005	0.367	0.071	320.69	0.42	1085.09	0.35	953.19	1395.09	237.67	3.25
VML006	-0.52	-1.09	303.91	0.53	892.48	0.28	650.85	1108.41	213.09	3.33
VML007	-0.26	0.835	415.76	0.53	1192.09	0.25	1615.29	2219.48	344.82	4.28
VML008	-0.45	0.311	407.37	0.56	1151.96	0.29	1192.28	1790.43	320.21	4.61
VML009	0.995	0.628	505.24	0.72	1210.82	0.23	1028.38	1550.91	280.30	4.18
VML010	-1.22	-0.78	275.95	0.48	855.03	0.27	772.07	1187.33	203.16	2.80
VML011	-0.32	0.246	371.02	0.59	1002.16	0.30	1192.50	1798.92	305.26	4.13
VML012	-1.54	-0.55	295.52	0.53	849.68	0.28	983.98	1430.17	223.82	2.80
VML015	-1.16	-0.84	329.08	0.57	913.88	0.30	630.22	1068.29	208.11	3.33
VML016	-1.32	0.534	362.63	0.53	1058.34	0.29	1324.06	1837.97	268.35	3.13
VML017	-0.9	-0.78	371.02	0.62	986.11	0.24	859.69	1411.84	274.66	4.32
VML018	0.305	-0.14	348.65	0.64	919.23	0.28	1153.20	1660.92	266.93	3.42
VML020	0.705	-0.38	351.45	0.57	970.06	0.31	878.79	1434.42	266.94	4.00
VML021	-1.02	-0.77	267.56	0.51	801.53	0.23	933.64	1345.69	208.56	2.58
VML022	-1.28	0.266	410.17	0.59	1122.54	0.28	1133.79	1702.55	281.03	3.72
VML023	-0.33	0.331	382.21	0.56	1061.01	0.30	1226.74	1787.02	290.23	3.77
VML024	-0.81	0.381	396.19	0.46	1269.67	0.29	1275.46	1853.24	298.68	3.83
VML025	-0.54	-1.4	273.15	0.60	769.43	0.26	586.47	1030.69	192.05	2.90
VML026	0.016	0.245	376.62	0.56	1047.64	0.30	1216.94	1795.55	284.63	3.59
VML027	-0.18	0.545	385.00	0.57	1063.69	0.33	1141.33	1704.06	300.08	4.25
VML028	0.908	-0.5	275.95	0.41	948.66	0.38	839.91	1329.34	232.77	3.26
VML030	1.539	1.299	508.04	0.56	1400.75	0.35	1195.23	1776.24	319.57	4.60
VML031	0.122	0.29	329.08	0.50	999.48	0.34	1255.02	1714.95	261.00	3.18
VML032	-0.23	0.821	387.80	0.53	1117.19	0.40	1404.55	1994.54	321.90	4.16
VML033	0.093	0.888	443.73	0.44	1440.87	0.40	991.55	1590.21	304.57	4.70
VML034	-0.41	0.218	393.39	0.51	1168.02	0.32	1167.75	1776.81	309.28	4.32
VML036	1.682	1.457	438.13	0.57	1197.44	0.38	1829.22	2473.67	366.28	4.31
VML039	-0.89	-0.22	348.65	0.54	991.46	0.29	950.93	1488.13	260.39	3.65
VML040	-0.28	0.552	354.25	0.56	994.13	0.33	1509.95	2144.57	335.02	4.18
VML041	1.209	0.901	393.39	0.48	1202.79	0.32	1110.24	1569.32	262.60	3.51

VML042	0.699	0.695	432.54	0.51	1272.34	0.34	1094.55	1722.14	333.21	5.20
VML043	-0.85	-1	278.75	0.62	750.70	0.26	961.90	1452.01	233.00	2.98
VML044	0.747	0.388	362.63	0.62	967.38	0.32	1389.06	1964.99	310.67	3.92
VML046	0.646	1.024	536.00	0.69	1301.77	0.31	1136.90	1750.29	326.92	4.94
VML047	0.466	-0.28	432.54	0.90	945.98	0.24	1212.00	1861.92	329.55	4.66
VML048	-0.76	-0.93	231.21	0.63	646.37	0.25	899.99	1262.95	186.52	2.20
VML049	-0.05	-0.21	326.28	0.54	940.63	0.24	1017.10	1463.03	237.27	3.07
VML050	-0.29	-1	405.98	0.59	1093.11	0.27	912.80	1455.43	279.22	4.34
VML051	-1.4	-1.41	275.95	0.59	790.83	0.24	806.21	1270.69	223.56	3.18
VML052	-0.67	0.03	298.32	0.34	1168.02	0.41	946.12	1368.25	227.37	3.04
VML053	0.453	0.206	404.58	0.56	1141.26	0.33	770.98	1266.64	243.13	3.79
VML054	-1.34	-0.32	368.23	0.55	1036.94	0.26	1253.99	1951.92	331.96	4.50
VML055	-0.33	0.144	301.12	0.37	1130.56	0.34	1314.53	1795.15	259.33	3.01
VML056	0.086	-0.27	357.04	0.64	945.98	0.32	992.32	1559.41	272.93	3.82
VML057	0.453	-0.02	398.99	0.71	978.08	0.29	812.35	1341.33	250.30	3.80
VML058	-0.51	-0.74	323.49	0.43	1069.04	0.30	788.16	1304.01	238.46	3.52
VML059	0.079	0.336	438.13	0.54	1234.89	0.28	1171.25	1709.68	291.34	4.00
VML061	-0.02	-1.6	270.36	0.68	705.22	0.24	595.73	1026.65	191.97	2.99
VML063	0.066	0.272	385.00	0.55	1101.14	0.27	1056.68	1550.87	264.10	3.65
VML064	0.598	0.253	376.62	0.55	1069.04	0.31	1250.15	1799.99	294.43	3.84
VML065	0.575	-0.42	323.49	0.59	881.78	0.30	1161.28	1689.61	276.94	3.62
VML066	-0.65	0.026	382.21	0.61	1018.21	0.28	1204.19	1796.35	309.22	4.27
VML067	-1.26	-1.13	273.15	0.53	801.53	0.23	831.58	1251.89	215.85	2.99
VML068	-0.17	0.543	424.15	0.59	1138.59	0.31	1137.72	1671.81	291.42	4.06
VML069	1.064	-0.11	306.71	0.61	822.93	0.33	1086.77	1530.35	239.01	2.98
VML070	0.279	0.244	301.12	0.50	905.86	0.37	1065.95	1569.38	250.41	3.19
VML073	-1.27	-0.25	295.52	0.48	905.86	0.24	1094.12	1532.26	230.71	2.77
VML074	1.423	-0.03	317.89	0.50	951.33	0.41	866.33	1413.50	252.27	3.62
VML075	0.671	-0.22	418.56	0.82	951.33	0.29	1081.96	1678.23	299.13	4.27
VML076	0.036	1.351	488.47	0.57	1341.90	0.34	1342.58	1937.15	315.47	4.10
VML080	0.546	0.259	379.41	0.56	1055.66	0.31	1106.79	1729.37	306.95	4.36
VML081	0.108	-0.69	354.25	0.63	929.93	0.27	892.20	1470.69	274.69	4.15
VML083	0.305	-0.83	278.75	0.49	849.68	0.29	861.50	1296.47	213.12	2.81
VML084	0.16	-0.58	354.25	0.62	951.33	0.24	1030.52	1604.97	288.02	4.15
VML085	-0.65	-0.05	312.30	0.40	1079.74	0.32	1013.07	1521.68	253.38	3.37
VML086	-1.29	-1.22	192.06	0.55	579.49	0.27	875.86	1220.01	170.78	1.93
VML087	0.843	0.446	468.89	0.62	1224.19	0.30	992.66	1547.65	288.41	4.48
VML088	1.55	-0.1	404.58	0.51	1189.42	0.30	836.13	1401.88	274.59	4.37
VML089	0.627	-0.34	340.26	0.51	1010.18	0.35	715.60	1190.36	228.90	3.58
VML090	-1	-0.75	253.58	0.41	879.10	0.29	726.86	1115.88	194.42	2.72
VML091	0.302	-0	393.39	0.65	1002.16	0.29	1044.75	1571.38	266.52	3.63
VML092	-0.06	0.272	345.86	0.45	1106.49	0.31	1017.53	1506.00	248.42	3.28
VML093	-0.48	-0.25	382.21	0.64	996.81	0.25	1022.94	1594.67	283.50	4.06
VML094	-1.03	-1.08	247.99	0.50	748.03	0.27	831.31	1260.53	201.20	2.56

VML095	-0.51	-0.7	315.10	0.56	884.46	0.30	941.41	1505.89	256.31	3.51
VML096	-0.53	-0.41	345.86	0.70	865.73	0.24	992.05	1465.18	242.16	3.20
VML097	-0.11	-0.04	382.21	0.52	1119.86	0.28	1057.64	1603.21	284.48	4.04
VML099	1.124	0.208	396.19	0.48	1226.87	0.29	910.62	1477.53	270.72	3.98
VML100	-0.54	0.134	362.63	0.42	1218.84	0.32	926.65	1457.66	258.41	3.67
VML102	-0.86	-0.01	340.26	0.57	959.36	0.32	1021.49	1554.51	266.19	3.64
VML104	-1.15	-0.78	287.14	0.58	798.85	0.28	755.68	1176.10	199.53	2.72
VML106	-0.79	0.665	480.08	0.69	1186.74	0.30	1363.13	1951.11	324.64	4.31
VML107	-0.9	0.014	359.84	0.61	964.71	0.28	1222.83	1776.22	274.93	3.40
VML110	-1.29	-0.52	337.47	0.68	860.38	0.27	991.61	1438.60	240.72	3.23
VML111	-0.81	-0.81	323.49	0.73	793.50	0.21	987.04	1444.40	238.41	3.15
VML112	0.443	0.459	440.93	0.55	1234.89	0.26	1057.31	1617.41	291.57	4.21
VML113	0.659	0.781	385.00	0.55	1087.76	0.38	1086.70	1617.28	282.99	3.97
VML114	0.53	0.901	468.89	0.65	1184.07	0.31	1187.06	1934.15	379.34	5.99
VML116	-0.67	-1.08	306.71	0.70	774.78	0.23	848.68	1307.25	222.77	3.04
VML117	-1.23	-0.3	259.17	0.44	857.70	0.33	931.91	1377.36	217.31	2.73
VML118	-0.03	1.294	552.78	0.58	1489.03	0.29	1301.20	1952.70	357.49	5.29
VML119	1.596	0.995	499.65	0.82	1119.86	0.32	1406.66	1987.62	334.78	4.50
VML120	0.595	-0.55	284.34	0.46	903.18	0.36	760.58	1217.81	215.46	3.06
VML121	1.634	1.355	485.67	0.50	1435.52	0.35	1228.44	1842.59	327.98	4.67
VML122	-0.24	-0.56	270.36	0.57	758.73	0.32	1098.37	1561.25	244.86	3.07
VML123	-0.13	0.668	382.21	0.44	1248.27	0.37	1055.68	1558.51	260.15	3.46
VML124	-0.07	-0.78	278.75	0.76	681.15	0.25	870.15	1271.03	195.80	2.41
VML125	-0.97	-1.23	306.71	0.71	766.75	0.14	688.08	1159.01	203.90	2.88
VML126	-0.07	0.357	351.45	0.40	1232.22	0.33	966.52	1444.73	236.03	3.08
VML127	-0.99	-0.97	259.17	0.52	785.48	0.30	915.01	1381.06	231.64	3.12
VML128	0.235	0.289	337.47	0.56	1010.18	0.32	1203.74	1701.20	259.14	3.14
VML129	-1.02	-1.17	261.97	0.61	737.33	0.25	859.18	1287.88	207.18	2.66
VML130	1.402	0.427	357.04	0.67	903.18	0.39	1388.45	1938.58	311.38	4.00
VML131	0.273	0.326	443.73	0.78	1028.91	0.29	1197.78	1766.57	321.19	4.69
VML132	-1.29	-1.06	250.78	0.48	793.50	0.28	977.09	1440.80	228.78	2.91
VML133	1.505	0.57	524.82	0.83	1159.99	0.27	1132.24	1737.98	317.28	4.71
VML134	-1.4	0.382	331.88	0.50	1007.51	0.32	1210.96	1699.84	267.03	3.34
VML136	0.439	0.263	552.78	0.68	1355.27	0.26	855.17	1479.47	308.30	5.23
VML137	1.737	0.936	371.02	0.48	1138.59	0.29	1482.44	2069.24	315.17	3.82
VML138	-1.14	-0.54	303.91	0.53	884.46	0.25	1231.40	1730.54	266.37	3.28
VML139	-0.13	0.237	345.86	0.40	1197.44	0.31	912.94	1351.83	231.97	3.19
VML140	-0.63	0.396	379.41	0.55	1071.71	0.36	1054.53	1717.01	311.27	4.52
VML141	-0.86	-0.18	275.95	0.38	1002.16	0.32	1299.67	1789.45	259.99	3.02
VML143	-0.05	0.435	390.60	0.51	1146.61	0.30	1110.26	1640.30	284.01	3.93
VML144	-1.31	1.153	471.69	0.60	1280.37	0.38	1419.62	2086.59	342.31	4.47
VML145	0.831	-0.02	289.93	0.40	1015.53	0.32	680.07	1058.82	184.18	2.57
VML146	0.578	0.296	348.65	0.50	1044.96	0.39	1102.59	1664.20	275.85	3.64
VML147	-0.39	-0.09	284.34	0.43	935.28	0.35	992.02	1489.78	243.06	3.16

VML148	0.696	0.343	371.02	0.55	1063.69	0.36	959.40	1471.37	249.88	3.39
VML150	0.07	0.265	385.00	0.67	970.06	0.30	1224.76	1757.57	290.13	3.82
VML151	-0.13	0.669	412.97	0.53	1189.42	0.26	1406.82	2010.60	337.95	4.53
VML152	2.076	-0.3	234.01	0.44	766.75	0.41	980.84	1399.45	210.25	2.52
VML153	2.37	0.595	303.91	0.41	1050.31	0.43	1029.38	1529.01	237.12	2.93
VML154	-1.32	0.225	323.49	0.54	929.93	0.32	1145.92	1629.15	261.17	3.36
VML155	-0.3	0.866	379.41	0.49	1149.29	0.36	1254.31	1759.87	272.72	3.37
VML156	0.483	-0.65	273.15	0.48	838.98	0.33	863.87	1325.73	222.18	2.97
VML157	0.646	0.425	426.95	0.69	1052.99	0.30	1272.58	1824.28	317.93	4.43
VML158	2.696	-0.44	239.60	0.43	812.23	0.42	773.22	1200.25	199.13	2.66
VML159	0.18	0.348	320.69	0.40	1117.19	0.32	965.50	1397.49	228.82	3.01
VML160	0.082	0.186	398.99	0.47	1226.87	0.29	1138.42	1724.69	312.82	4.60
VML161	0.135	-0.2	351.45	0.51	1042.29	0.32	710.62	1147.44	215.53	3.28
VML162	-0.83	-1.28	264.77	0.57	748.03	0.24	696.69	1133.73	200.85	2.86
VML164	0.011	-0.15	373.82	0.57	1042.29	0.31	931.35	1494.98	272.20	3.99
VML165	1.223	0.95	502.45	0.57	1371.32	0.28	1038.16	1604.57	292.67	4.31
VML168	0.02	-0.17	301.12	0.53	873.75	0.35	784.65	1201.48	214.57	3.09
VML170	-0.41	-0.72	301.12	0.54	871.08	0.33	1031.60	1544.92	254.88	3.36
VML171	0.629	-0.27	329.08	0.54	948.66	0.27	963.25	1489.68	263.91	3.76
VML173	-1.17	-0.24	376.62	0.63	983.43	0.30	1050.25	1604.16	284.04	4.03
VML174	1.345	0.882	410.17	0.53	1186.74	0.37	1152.01	1712.76	293.58	4.02
VML175	-1.45	-1.05	228.41	0.38	838.98	0.29	983.74	1384.74	208.40	2.50
VML176	0.047	0.289	385.00	0.44	1245.59	0.33	1138.66	1711.72	300.73	4.23
VML178	-1.39	-0.03	348.65	0.48	1082.41	0.30	938.36	1396.11	233.57	3.13
VML179	1.051	0.801	410.17	0.64	1061.01	0.33	1127.82	1642.43	272.18	3.60
VML180	0.117	-0.59	320.69	0.47	1004.83	0.25	711.92	1116.44	203.62	3.01
VML183	0.817	0.349	385.00	0.59	1050.31	0.36	1136.26	1714.22	287.35	3.85
VML184	1.045	-0.33	354.25	0.58	988.78	0.29	1089.27	1614.86	276.58	3.78
VML188	-0.94	-0.16	379.41	0.46	1216.17	0.28	737.43	1285.94	254.45	4.10

			Non-applied phosphorus	
**Inbred Lines**	**LPTI**	**LPPI**	**RAD**	**RTD**
VML001	1.318	0.999	0.47	106.14
VML002	3.139	0.491	0.45	114.87
VML003	0.454	0.98	0.53	99.22
VML004	0.78	1.105	0.60	107.97
VML005	0.367	0.071	0.54	101.26
VML006	-0.52	-1.09	0.64	91.23
VML007	-0.26	0.835	0.50	96.37
VML008	-0.45	0.311	0.57	89.32
VML009	0.995	0.628	0.59	120.96
VML010	-1.22	-0.78	0.56	99.82
VML011	-0.32	0.246	0.54	89.88
VML012	-1.54	-0.55	0.50	104.24
VML015	-1.16	-0.84	0.65	99.51

VML016	-1.32	0.534	0.47	115.77
VML017	-0.9	-0.78	0.63	85.70
VML018	0.305	-0.14	0.51	102.39
VML020	0.705	-0.38	0.60	88.53
VML021	-1.02	-0.77	0.50	105.51
VML022	-1.28	0.266	0.53	110.15
VML023	-0.33	0.331	0.52	101.44
VML024	-0.81	0.381	0.51	103.10
VML025	-0.54	-1.4	0.62	95.07
VML026	0.016	0.245	0.50	104.72
VML027	-0.18	0.545	0.56	91.64
VML028	0.908	-0.5	0.56	85.68
VML030	1.539	1.299	0.57	109.51
VML031	0.122	0.29	0.49	104.48
VML032	-0.23	0.821	0.52	94.01
VML033	0.093	0.888	0.61	94.76
VML034	-0.41	0.218	0.55	92.12
VML036	1.682	1.457	0.47	101.55
VML039	-0.89	-0.22	0.56	95.62
VML040	-0.28	0.552	0.50	85.31
VML041	1.209	0.901	0.53	112.23
VML042	0.699	0.695	0.61	85.07
VML043	-0.85	-1	0.51	94.62
VML044	0.747	0.388	0.51	92.83
VML046	0.646	1.024	0.60	107.18
VML047	0.466	-0.28	0.56	92.77
VML048	-0.76	-0.93	0.47	107.98
VML049	-0.05	-0.21	0.52	107.57
VML050	-0.29	-1	0.62	80.67
VML051	-1.4	-1.41	0.57	88.50
VML052	-0.67	0.03	0.53	99.94
VML053	0.453	0.206	0.62	106.58
VML054	-1.34	-0.32	0.54	82.71
VML055	-0.33	0.144	0.47	101.17
VML056	0.086	-0.27	0.56	93.74
VML057	0.453	-0.02	0.61	104.92
VML058	-0.51	-0.74	0.60	91.46
VML059	0.079	0.336	0.54	112.07
VML061	-0.02	-1.6	0.66	90.73
VML063	0.066	0.272	0.56	105.46
VML064	0.598	0.253	0.52	97.81
VML065	0.575	-0.42	0.52	89.52
VML066	-0.65	0.026	0.55	89.78
VML067	-1.26	-1.13	0.56	91.72

VML068	-0.17	0.543	0.55	104.07
VML069	1.064	-0.11	0.50	103.60
VML070	0.279	0.244	0.51	94.80
VML073	-1.27	-0.25	0.48	107.99
VML074	1.423	-0.03	0.57	89.77
VML075	0.671	-0.22	0.57	97.78
VML076	0.036	1.351	0.52	118.74
VML080	0.546	0.259	0.57	87.19
VML081	0.108	-0.69	0.61	86.50
VML083	0.305	-0.83	0.53	99.96
VML084	0.16	-0.58	0.58	86.09
VML085	-0.65	-0.05	0.53	92.60
VML086	-1.29	-1.22	0.45	104.77
VML087	0.843	0.446	0.61	106.17
VML088	1.55	-0.1	0.63	93.11
VML089	0.627	-0.34	0.63	95.43
VML090	-1	-0.75	0.57	93.97
VML091	0.302	-0	0.54	109.08
VML092	-0.06	0.272	0.53	106.32
VML093	-0.48	-0.25	0.58	94.65
VML094	-1.03	-1.08	0.51	96.83
VML095	-0.51	-0.7	0.54	88.92
VML096	-0.53	-0.41	0.53	108.54
VML097	-0.11	-0.04	0.57	95.33
VML099	1.124	0.208	0.59	99.43
VML100	-0.54	0.134	0.57	97.39
VML102	-0.86	-0.01	0.55	93.72
VML104	-1.15	-0.78	0.55	106.87
VML106	-0.79	0.665	0.53	110.81
VML107	-0.9	0.014	0.50	105.74
VML110	-1.29	-0.52	0.54	104.92
VML111	-0.81	-0.81	0.53	103.53
VML112	0.443	0.459	0.58	104.21
VML113	0.659	0.781	0.56	97.07
VML114	0.53	0.901	0.62	79.68
VML116	-0.67	-1.08	0.55	101.21
VML117	-1.23	-0.3	0.50	96.02
VML118	-0.03	1.294	0.59	103.42
VML119	1.596	0.995	0.53	109.66
VML120	0.595	-0.55	0.57	94.08
VML121	1.634	1.355	0.56	103.86
VML122	-0.24	-0.56	0.50	87.57
VML123	-0.13	0.668	0.53	110.83
VML124	-0.07	-0.78	0.49	118.67

VML125	-0.97	-1.23	0.57	108.66
VML126	-0.07	0.357	0.53	114.95
VML127	-0.99	-0.97	0.55	83.49
VML128	0.235	0.289	0.48	112.24
VML129	-1.02	-1.17	0.52	99.72
VML130	1.402	0.427	0.51	89.21
VML131	0.273	0.326	0.58	95.01
VML132	-1.29	-1.06	0.51	87.27
VML133	1.505	0.57	0.60	109.97
VML134	-1.4	0.382	0.50	99.97
VML136	0.439	0.263	0.67	104.17
VML137	1.737	0.936	0.48	97.39
VML138	-1.14	-0.54	0.49	92.90
VML139	-0.13	0.237	0.55	109.41
VML140	-0.63	0.396	0.58	85.09
VML141	-0.86	-0.18	0.47	92.23
VML143	-0.05	0.435	0.55	99.64
VML144	-1.31	1.153	0.52	104.47
VML145	0.831	-0.02	0.56	118.35
VML146	0.578	0.296	0.53	95.88
VML147	-0.39	-0.09	0.52	90.19
VML148	0.696	0.343	0.54	109.93
VML150	0.07	0.265	0.53	101.03
VML151	-0.13	0.669	0.53	91.38
VML152	2.076	-0.3	0.48	93.94
VML153	2.37	0.595	0.49	104.87
VML154	-1.32	0.225	0.51	96.30
VML155	-0.3	0.866	0.50	113.12
VML156	0.483	-0.65	0.54	92.17
VML157	0.646	0.425	0.55	96.60
VML158	2.696	-0.44	0.53	92.26
VML159	0.18	0.348	0.53	108.09
VML160	0.082	0.186	0.58	87.01
VML161	0.135	-0.2	0.61	107.62
VML162	-0.83	-1.28	0.58	93.13
VML164	0.011	-0.15	0.58	94.64
VML165	1.223	0.95	0.59	115.34
VML168	0.02	-0.17	0.57	98.13
VML170	-0.41	-0.72	0.53	90.10
VML171	0.629	-0.27	0.57	89.64
VML173	-1.17	-0.24	0.56	93.27
VML174	1.345	0.882	0.55	101.72
VML175	-1.45	-1.05	0.48	91.26
VML176	0.047	0.289	0.56	91.36

VML178	-1.39	-0.03	0.54	112.36
VML179	1.051	0.801	0.53	114.05
VML180	0.117	-0.59	0.60	107.62
VML183	0.817	0.349	0.53	102.24
VML184	1.045	-0.33	0.65	92.84
VML188	-0.94	-0.16	0.55	93.86

SD: stalk diameter (mm); PH: plant height (cm); SDW: shoot dry weight (mg); RDW: root dry weight (mg); RSR: root dry weight to shoot dry weight ratio; TDW: total dry weight (mg); DG: daily growth (cm); LRL: lateral root length (cm); TRL: total root length (cm); RSA: root surface area (cm2); RV: root volume (cm3); RAD: root average diameter (mm) and RTD: root tissue density (mg cm-3).
