# Supplementary material for: Genetic diversity, relationships among traits and selection of tropical maize inbred lines for low-P tolerance based on root and shoot traits at seedling stage
Source: Front Plant Sci. 2024 Oct 1;15:1429901. doi: 10.3389/fpls.2024.1429901 (PMC11473326; doi:10.3389/fpls.2024.1429901)
Supplement: Supplementary file 1 [file Table1.pdf]

Supplementary Material

Genetic diversity, relationships among traits and selection of tropical maize inbred lines for low-P tolerance based on root and shoot traits at seedling stage

Supplementary table 1. Genetic distance matrix for 151 tropical maize inbred lines using standardized mean Euclidean distance based on eight traits: stalk diameter, plant height, root:shoot dry weight ratio, daily growth, lateral root length, root volume, root average diameter and root tissue density measured under non-applied P (above diagonal) and applied P (below diagonal).

Lines	VML001	VML002	VML003	VML004	VML005	VML006	VML007	VML008
VML001		0.186	0.121	0.258	0.266	0.368	0.159	0.200
VML002	0.261		0.196	0.239	0.169	0.265	0.259	0.228
VML003	0.210	0.327		0.155	0.217	0.317	0.213	0.149
VML004	0.199	0.351	0.063		0.190	0.251	0.293	0.157
VML005	0.138	0.266	0.090	0.094		0.185	0.300	0.202
VML006	0.198	0.338	0.088	0.045	0.095		0.357	0.230
VML007	0.158	0.359	0.175	0.129	0.148	0.127		0.170
VML008	0.264	0.441	0.219	0.177	0.217	0.188	0.142	
VML009	0.169	0.252	0.275	0.258	0.191	0.249	0.231	0.288
VML010	0.191	0.369	0.147	0.090	0.128	0.090	0.102	0.152
VML011	0.177	0.368	0.146	0.093	0.125	0.104	0.094	0.147
VML012	0.219	0.389	0.091	0.053	0.125	0.087	0.146	0.162
VML015	0.309	0.446	0.149	0.117	0.201	0.131	0.203	0.185
VML016	0.266	0.475	0.207	0.159	0.227	0.160	0.171	0.215
VML017	0.249	0.434	0.208	0.164	0.201	0.174	0.143	0.049
VML018	0.067	0.238	0.180	0.179	0.103	0.183	0.169	0.254
VML020	0.202	0.225	0.178	0.197	0.127	0.201	0.236	0.261
VML021	0.083	0.304	0.186	0.160	0.116	0.157	0.135	0.223
VML022	0.322	0.508	0.240	0.197	0.267	0.206	0.183	0.117
VML023	0.161	0.333	0.151	0.110	0.109	0.113	0.127	0.197
VML024	0.217	0.402	0.165	0.124	0.156	0.138	0.133	0.092
VML025	0.184	0.261	0.109	0.139	0.078	0.153	0.207	0.239
VML026	0.112	0.286	0.114	0.111	0.058	0.123	0.150	0.228
VML027	0.230	0.433	0.157	0.105	0.179	0.122	0.131	0.180
VML028	0.129	0.220	0.146	0.160	0.088	0.162	0.168	0.236
VML030	0.097	0.285	0.175	0.158	0.100	0.163	0.147	0.210
VML031	0.156	0.274	0.086	0.118	0.080	0.128	0.184	0.268
VML032	0.247	0.440	0.137	0.108	0.180	0.139	0.168	0.181
VML033	0.297	0.464	0.149	0.120	0.207	0.148	0.192	0.167
VML034	0.229	0.435	0.199	0.156	0.191	0.177	0.150	0.107
VML036	0.040	0.264	0.206	0.195	0.131	0.198	0.158	0.245
VML039	0.269	0.429	0.237	0.185	0.220	0.184	0.150	0.102
VML040	0.203	0.418	0.162	0.139	0.171	0.171	0.162	0.174
VML041	0.131	0.200	0.195	0.195	0.111	0.188	0.203	0.274

VML042	0.197	0.368	0.101	0.077	0.117	0.112	0.126	0.132
VML043	0.103	0.290	0.112	0.106	0.063	0.120	0.128	0.215
VML044	0.065	0.279	0.198	0.179	0.122	0.180	0.139	0.222
VML046	0.196	0.427	0.239	0.190	0.208	0.197	0.121	0.133
VML047	0.093	0.230	0.170	0.173	0.094	0.176	0.166	0.235
VML048	0.084	0.235	0.218	0.213	0.136	0.213	0.200	0.281
VML049	0.099	0.230	0.201	0.197	0.118	0.197	0.191	0.262
VML050	0.149	0.344	0.106	0.088	0.095	0.116	0.121	0.172
VML051	0.201	0.376	0.108	0.095	0.122	0.130	0.160	0.162
VML052	0.272	0.421	0.143	0.108	0.175	0.119	0.162	0.115
VML053	0.170	0.338	0.166	0.135	0.125	0.145	0.116	0.118
VML054	0.307	0.509	0.241	0.194	0.268	0.204	0.166	0.151
VML055	0.201	0.303	0.100	0.121	0.115	0.114	0.204	0.291
VML056	0.164	0.312	0.154	0.149	0.104	0.163	0.191	0.211
VML057	0.158	0.262	0.148	0.151	0.088	0.161	0.188	0.230
VML058	0.188	0.335	0.078	0.030	0.081	0.031	0.120	0.173
VML059	0.113	0.297	0.144	0.112	0.085	0.103	0.074	0.177
VML061	0.155	0.250	0.120	0.151	0.081	0.166	0.212	0.268
VML063	0.126	0.272	0.124	0.105	0.049	0.097	0.120	0.197
VML064	0.085	0.236	0.178	0.170	0.101	0.169	0.141	0.225
VML065	0.102	0.200	0.188	0.193	0.104	0.189	0.202	0.282
VML066	0.208	0.417	0.158	0.114	0.164	0.138	0.114	0.113
VML067	0.163	0.322	0.110	0.075	0.078	0.083	0.101	0.143
VML068	0.180	0.385	0.136	0.088	0.127	0.102	0.111	0.151
VML069	0.173	0.163	0.206	0.235	0.146	0.236	0.273	0.340
VML070	0.131	0.268	0.123	0.112	0.045	0.109	0.150	0.220
VML073	0.143	0.333	0.159	0.111	0.113	0.103	0.077	0.172
VML074	0.177	0.201	0.158	0.188	0.112	0.196	0.229	0.280
VML075	0.119	0.274	0.198	0.198	0.129	0.208	0.211	0.265
VML076	0.229	0.380	0.079	0.084	0.134	0.113	0.194	0.240
VML080	0.139	0.358	0.179	0.144	0.148	0.152	0.062	0.140
VML081	0.173	0.296	0.200	0.196	0.142	0.210	0.209	0.221
VML083	0.198	0.213	0.244	0.262	0.179	0.268	0.290	0.329
VML084	0.146	0.273	0.214	0.204	0.147	0.208	0.172	0.199
VML085	0.220	0.364	0.078	0.045	0.123	0.065	0.138	0.193
VML086	0.213	0.210	0.144	0.181	0.121	0.180	0.255	0.323
VML087	0.122	0.313	0.140	0.123	0.083	0.130	0.150	0.213
VML088	0.172	0.187	0.206	0.212	0.136	0.204	0.212	0.263
VML089	0.178	0.289	0.051	0.081	0.069	0.087	0.159	0.227
VML090	0.211	0.360	0.157	0.103	0.139	0.085	0.114	0.168
VML091	0.094	0.294	0.166	0.161	0.107	0.175	0.157	0.216
VML092	0.143	0.336	0.113	0.088	0.106	0.095	0.105	0.210
VML093	0.161	0.349	0.114	0.069	0.107	0.083	0.065	0.149
VML094	0.116	0.280	0.134	0.118	0.058	0.120	0.131	0.193

VML095	0.164	0.285	0.088	0.089	0.043	0.099	0.162	0.213
VML096	0.094	0.328	0.200	0.169	0.138	0.172	0.108	0.184
VML097	0.144	0.353	0.135	0.097	0.105	0.111	0.103	0.158
VML099	0.073	0.232	0.171	0.165	0.087	0.163	0.152	0.239
VML100	0.235	0.380	0.126	0.076	0.135	0.067	0.137	0.149
VML102	0.260	0.405	0.116	0.075	0.155	0.089	0.155	0.143
VML104	0.219	0.344	0.048	0.053	0.099	0.070	0.177	0.223
VML106	0.241	0.441	0.182	0.123	0.187	0.130	0.131	0.136
VML107	0.159	0.364	0.158	0.104	0.125	0.102	0.090	0.166
VML110	0.215	0.424	0.170	0.117	0.168	0.130	0.126	0.138
VML111	0.121	0.302	0.228	0.206	0.156	0.209	0.157	0.198
VML112	0.099	0.311	0.188	0.154	0.118	0.150	0.114	0.197
VML113	0.238	0.319	0.065	0.105	0.114	0.120	0.220	0.263
VML114	0.216	0.411	0.150	0.105	0.161	0.132	0.107	0.097
VML116	0.107	0.237	0.174	0.159	0.090	0.147	0.146	0.234
VML117	0.241	0.443	0.163	0.110	0.186	0.130	0.136	0.165
VML118	0.330	0.541	0.348	0.297	0.325	0.306	0.251	0.186
VML119	0.183	0.326	0.285	0.280	0.221	0.292	0.282	0.322
VML120	0.151	0.210	0.159	0.167	0.095	0.165	0.178	0.237
VML121	0.142	0.248	0.111	0.130	0.052	0.141	0.179	0.235
VML122	0.116	0.272	0.108	0.111	0.049	0.113	0.156	0.243
VML123	0.194	0.381	0.141	0.081	0.130	0.078	0.104	0.154
VML124	0.129	0.193	0.254	0.259	0.172	0.257	0.248	0.313
VML125	0.225	0.352	0.363	0.339	0.286	0.338	0.279	0.303
VML126	0.165	0.308	0.076	0.068	0.057	0.062	0.148	0.216
VML127	0.204	0.374	0.141	0.118	0.135	0.143	0.143	0.111
VML128	0.138	0.221	0.176	0.178	0.096	0.176	0.195	0.252
VML129	0.108	0.307	0.179	0.149	0.117	0.149	0.088	0.161
VML130	0.139	0.201	0.174	0.195	0.108	0.201	0.219	0.277
VML131	0.191	0.437	0.247	0.201	0.221	0.206	0.120	0.166
VML132	0.184	0.309	0.078	0.075	0.058	0.086	0.161	0.195
VML133	0.166	0.313	0.301	0.285	0.228	0.291	0.235	0.266
VML134	0.353	0.530	0.224	0.196	0.277	0.207	0.266	0.277
VML136	0.213	0.312	0.191	0.175	0.148	0.176	0.176	0.162
VML137	0.135	0.193	0.258	0.258	0.174	0.254	0.246	0.319
VML138	0.166	0.389	0.164	0.129	0.159	0.134	0.118	0.214
VML139	0.176	0.284	0.142	0.123	0.094	0.121	0.149	0.202
VML140	0.412	0.622	0.354	0.307	0.377	0.317	0.282	0.206
VML141	0.227	0.361	0.106	0.115	0.149	0.120	0.203	0.283
VML143	0.157	0.345	0.164	0.126	0.124	0.139	0.096	0.128
VML144	0.453	0.648	0.333	0.301	0.386	0.320	0.339	0.289
VML145	0.134	0.180	0.199	0.201	0.118	0.191	0.201	0.278
VML146	0.130	0.283	0.108	0.098	0.045	0.106	0.143	0.214
VML147	0.254	0.368	0.071	0.111	0.146	0.133	0.229	0.269

VML148	0.167	0.217	0.140	0.153	0.085	0.149	0.182	0.238
VML150	0.117	0.283	0.128	0.109	0.052	0.107	0.125	0.195
VML151	0.159	0.350	0.144	0.096	0.119	0.097	0.041	0.129
VML152	0.229	0.094	0.257	0.292	0.207	0.287	0.324	0.397
VML153	0.171	0.122	0.228	0.254	0.164	0.251	0.275	0.353
VML154	0.301	0.458	0.156	0.116	0.204	0.127	0.199	0.197
VML155	0.235	0.364	0.084	0.098	0.122	0.124	0.214	0.234
VML156	0.098	0.267	0.222	0.215	0.148	0.221	0.204	0.278
VML157	0.096	0.304	0.173	0.157	0.112	0.169	0.132	0.189
VML158	0.253	0.105	0.295	0.327	0.240	0.326	0.351	0.409
VML159	0.141	0.297	0.196	0.159	0.132	0.146	0.109	0.198
VML160	0.166	0.350	0.121	0.088	0.106	0.108	0.092	0.120
VML161	0.210	0.300	0.097	0.111	0.084	0.109	0.208	0.244
VML162	0.136	0.288	0.119	0.097	0.083	0.087	0.098	0.207
VML164	0.186	0.305	0.057	0.067	0.060	0.081	0.152	0.188
VML165	0.080	0.277	0.206	0.181	0.126	0.173	0.131	0.221
VML168	0.184	0.282	0.085	0.094	0.056	0.099	0.178	0.228
VML170	0.172	0.298	0.160	0.159	0.114	0.177	0.190	0.209
VML171	0.112	0.283	0.251	0.230	0.173	0.231	0.181	0.250
VML173	0.296	0.504	0.236	0.183	0.250	0.196	0.176	0.125
VML174	0.139	0.222	0.137	0.152	0.068	0.157	0.188	0.250
VML175	0.206	0.316	0.040	0.069	0.084	0.079	0.182	0.240
VML176	0.213	0.407	0.219	0.169	0.193	0.171	0.094	0.075
VML178	0.265	0.413	0.146	0.102	0.166	0.116	0.165	0.137
VML179	0.108	0.234	0.164	0.172	0.089	0.176	0.199	0.273
VML180	0.112	0.219	0.157	0.159	0.073	0.154	0.167	0.237
VML183	0.118	0.266	0.115	0.131	0.063	0.146	0.169	0.233
VML184	0.128	0.242	0.111	0.137	0.062	0.147	0.179	0.250
VML188	0.249	0.415	0.131	0.093	0.159	0.109	0.150	0.121

	VML009	VML010	VML011	VML012	VML015	VML016	VML017	VML018
VML001	0.302	0.336	0.179	0.266	0.366	0.156	0.330	0.199
VML002	0.265	0.211	0.180	0.142	0.255	0.096	0.291	0.135
VML003	0.288	0.312	0.135	0.262	0.307	0.192	0.287	0.197
VML004	0.249	0.271	0.159	0.260	0.229	0.244	0.241	0.222
VML005	0.301	0.154	0.180	0.154	0.183	0.180	0.254	0.195
VML006	0.253	0.098	0.218	0.157	0.056	0.267	0.146	0.201
VML007	0.252	0.323	0.173	0.270	0.373	0.181	0.281	0.206
VML008	0.200	0.235	0.056	0.208	0.239	0.187	0.159	0.148
VML009		0.249	0.196	0.227	0.252	0.232	0.173	0.171
VML010	0.216		0.212	0.090	0.125	0.208	0.190	0.174
VML011	0.213	0.032		0.173	0.224	0.152	0.167	0.106
VML012	0.277	0.099	0.095		0.170	0.135	0.200	0.106
VML015	0.336	0.142	0.154	0.108		0.270	0.169	0.205

VML016	0.350	0.157	0.160	0.143	0.177		0.264	0.116
VML017	0.272	0.136	0.131	0.143	0.181	0.197		0.172
VML018	0.142	0.177	0.163	0.199	0.285	0.280	0.240	
VML020	0.170	0.214	0.211	0.220	0.272	0.342	0.252	0.143
VML021	0.153	0.131	0.119	0.170	0.258	0.217	0.198	0.089
VML022	0.378	0.196	0.197	0.178	0.177	0.170	0.125	0.324
VML023	0.177	0.061	0.058	0.126	0.181	0.194	0.180	0.138
VML024	0.248	0.106	0.099	0.099	0.161	0.177	0.057	0.202
VML025	0.206	0.178	0.172	0.157	0.222	0.286	0.227	0.129
VML026	0.194	0.132	0.119	0.127	0.219	0.216	0.209	0.083
VML027	0.304	0.102	0.098	0.092	0.136	0.082	0.167	0.230
VML028	0.188	0.188	0.179	0.191	0.261	0.289	0.232	0.095
VML030	0.134	0.138	0.124	0.169	0.255	0.249	0.189	0.065
VML031	0.251	0.177	0.170	0.148	0.224	0.235	0.255	0.133
VML032	0.332	0.147	0.137	0.071	0.139	0.115	0.164	0.240
VML033	0.355	0.157	0.157	0.091	0.088	0.150	0.159	0.281
VML034	0.262	0.123	0.105	0.122	0.182	0.180	0.078	0.217
VML036	0.149	0.184	0.167	0.210	0.301	0.273	0.230	0.049
VML039	0.250	0.123	0.130	0.183	0.181	0.228	0.109	0.255
VML040	0.293	0.155	0.133	0.104	0.199	0.162	0.152	0.198
VML041	0.101	0.188	0.186	0.224	0.290	0.314	0.258	0.083
VML042	0.256	0.110	0.095	0.066	0.144	0.186	0.123	0.172
VML043	0.201	0.129	0.113	0.127	0.218	0.210	0.200	0.082
VML044	0.130	0.153	0.137	0.192	0.277	0.255	0.206	0.060
VML046	0.245	0.138	0.123	0.173	0.241	0.181	0.110	0.208
VML047	0.149	0.180	0.167	0.194	0.275	0.286	0.225	0.051
VML048	0.111	0.197	0.185	0.230	0.313	0.304	0.263	0.053
VML049	0.099	0.183	0.172	0.216	0.294	0.301	0.245	0.049
VML050	0.233	0.113	0.092	0.083	0.183	0.179	0.156	0.131
VML051	0.261	0.129	0.113	0.064	0.160	0.188	0.140	0.175
VML052	0.305	0.130	0.138	0.093	0.096	0.175	0.107	0.253
VML053	0.181	0.108	0.096	0.137	0.199	0.230	0.107	0.145
VML054	0.382	0.191	0.189	0.179	0.187	0.133	0.158	0.317
VML055	0.284	0.182	0.186	0.155	0.210	0.215	0.275	0.187
VML056	0.176	0.150	0.138	0.144	0.228	0.254	0.183	0.120
VML057	0.147	0.152	0.144	0.168	0.232	0.283	0.216	0.098
VML058	0.236	0.077	0.085	0.070	0.127	0.169	0.159	0.167
VML059	0.177	0.096	0.091	0.139	0.210	0.199	0.165	0.110
VML061	0.210	0.192	0.181	0.169	0.248	0.282	0.253	0.105
VML063	0.161	0.103	0.103	0.136	0.205	0.223	0.181	0.098
VML064	0.135	0.163	0.152	0.197	0.271	0.278	0.217	0.057
VML065	0.127	0.195	0.189	0.219	0.298	0.304	0.265	0.057
VML066	0.277	0.110	0.093	0.085	0.161	0.138	0.094	0.203
VML067	0.198	0.075	0.072	0.095	0.161	0.197	0.130	0.137

VML068	0.246	0.080	0.070	0.070	0.160	0.127	0.122	0.175
VML069	0.189	0.260	0.253	0.260	0.333	0.363	0.324	0.125
VML070	0.156	0.113	0.112	0.138	0.209	0.231	0.201	0.093
VML073	0.183	0.056	0.057	0.131	0.191	0.176	0.157	0.139
VML074	0.196	0.221	0.213	0.216	0.277	0.333	0.273	0.121
VML075	0.150	0.194	0.178	0.202	0.294	0.291	0.241	0.077
VML076	0.305	0.151	0.147	0.081	0.147	0.166	0.221	0.208
VML080	0.233	0.132	0.114	0.148	0.227	0.186	0.135	0.153
VML081	0.141	0.182	0.168	0.198	0.268	0.308	0.205	0.120
VML083	0.158	0.268	0.258	0.276	0.347	0.387	0.312	0.142
VML084	0.137	0.188	0.175	0.216	0.285	0.310	0.193	0.114
VML085	0.284	0.109	0.113	0.080	0.109	0.161	0.189	0.203
VML086	0.237	0.226	0.228	0.217	0.263	0.321	0.311	0.168
VML087	0.184	0.121	0.109	0.126	0.221	0.204	0.184	0.101
VML088	0.140	0.215	0.214	0.244	0.297	0.344	0.259	0.130
VML089	0.255	0.153	0.152	0.121	0.183	0.218	0.219	0.155
VML090	0.221	0.051	0.079	0.128	0.143	0.177	0.158	0.197
VML091	0.185	0.167	0.147	0.164	0.263	0.247	0.196	0.074
VML092	0.255	0.121	0.112	0.113	0.193	0.152	0.198	0.149
VML093	0.238	0.080	0.070	0.090	0.160	0.156	0.143	0.155
VML094	0.151	0.111	0.102	0.135	0.215	0.229	0.174	0.081
VML095	0.192	0.117	0.115	0.114	0.180	0.230	0.197	0.121
VML096	0.172	0.137	0.119	0.172	0.260	0.217	0.165	0.109
VML097	0.217	0.090	0.073	0.090	0.187	0.159	0.132	0.137
VML099	0.132	0.161	0.151	0.191	0.271	0.270	0.225	0.036
VML100	0.258	0.078	0.099	0.088	0.104	0.164	0.132	0.216
VML102	0.296	0.108	0.118	0.070	0.064	0.163	0.135	0.239
VML104	0.273	0.127	0.132	0.079	0.127	0.184	0.208	0.192
VML106	0.284	0.084	0.087	0.098	0.134	0.103	0.113	0.237
VML107	0.210	0.051	0.049	0.109	0.178	0.141	0.143	0.159
VML110	0.269	0.089	0.080	0.086	0.152	0.113	0.110	0.212
VML111	0.122	0.170	0.155	0.212	0.289	0.284	0.184	0.107
VML112	0.142	0.111	0.101	0.165	0.245	0.216	0.175	0.099
VML113	0.281	0.173	0.176	0.131	0.162	0.244	0.253	0.200
VML114	0.274	0.100	0.084	0.084	0.142	0.156	0.092	0.205
VML116	0.117	0.141	0.140	0.190	0.254	0.264	0.221	0.080
VML117	0.308	0.104	0.098	0.087	0.125	0.089	0.153	0.238
VML118	0.322	0.234	0.226	0.269	0.300	0.270	0.166	0.330
VML119	0.175	0.259	0.241	0.275	0.362	0.350	0.296	0.157
VML120	0.160	0.181	0.177	0.201	0.257	0.304	0.234	0.107
VML121	0.183	0.160	0.151	0.154	0.228	0.268	0.222	0.088
VML122	0.201	0.140	0.134	0.136	0.221	0.217	0.224	0.094
VML123	0.238	0.041	0.053	0.082	0.138	0.124	0.131	0.187
VML124	0.117	0.252	0.242	0.278	0.358	0.363	0.297	0.099

VML125	0.139	0.285	0.273	0.343	0.409	0.400	0.290	0.219
VML126	0.232	0.118	0.121	0.096	0.173	0.183	0.195	0.147
VML127	0.241	0.125	0.110	0.099	0.169	0.211	0.096	0.178
VML128	0.111	0.173	0.169	0.202	0.269	0.302	0.236	0.082
VML129	0.161	0.121	0.107	0.162	0.238	0.226	0.151	0.105
VML130	0.164	0.214	0.204	0.218	0.294	0.326	0.264	0.081
VML131	0.278	0.164	0.148	0.187	0.259	0.161	0.150	0.219
VML132	0.211	0.110	0.111	0.093	0.157	0.215	0.177	0.145
VML133	0.130	0.248	0.231	0.288	0.366	0.359	0.254	0.153
VML134	0.422	0.221	0.226	0.170	0.160	0.123	0.259	0.349
VML136	0.169	0.156	0.156	0.188	0.224	0.294	0.160	0.173
VML137	0.087	0.242	0.234	0.280	0.354	0.361	0.303	0.101
VML138	0.284	0.137	0.127	0.130	0.211	0.108	0.197	0.188
VML139	0.159	0.106	0.111	0.155	0.193	0.253	0.195	0.134
VML140	0.464	0.288	0.286	0.276	0.271	0.228	0.207	0.420
VML141	0.323	0.181	0.184	0.139	0.188	0.176	0.268	0.220
VML143	0.182	0.086	0.066	0.129	0.194	0.211	0.118	0.136
VML144	0.516	0.312	0.312	0.263	0.238	0.219	0.281	0.450
VML145	0.113	0.197	0.195	0.234	0.295	0.322	0.268	0.094
VML146	0.177	0.109	0.101	0.121	0.199	0.219	0.197	0.093
VML147	0.330	0.193	0.193	0.119	0.165	0.211	0.255	0.229
VML148	0.179	0.177	0.177	0.190	0.241	0.296	0.234	0.123
VML150	0.161	0.105	0.099	0.129	0.208	0.216	0.176	0.089
VML151	0.221	0.076	0.069	0.112	0.174	0.164	0.122	0.158
VML152	0.246	0.325	0.322	0.325	0.387	0.424	0.387	0.195
VML153	0.188	0.275	0.269	0.285	0.355	0.380	0.341	0.132
VML154	0.341	0.135	0.148	0.100	0.053	0.136	0.184	0.285
VML155	0.280	0.154	0.152	0.089	0.152	0.209	0.210	0.200
VML156	0.127	0.195	0.179	0.225	0.311	0.299	0.259	0.065
VML157	0.168	0.146	0.126	0.162	0.254	0.241	0.172	0.078
VML158	0.237	0.351	0.345	0.356	0.418	0.464	0.401	0.211
VML159	0.136	0.107	0.109	0.186	0.235	0.239	0.190	0.132
VML160	0.228	0.098	0.083	0.089	0.169	0.183	0.108	0.150
VML161	0.229	0.152	0.159	0.128	0.181	0.239	0.222	0.172
VML162	0.210	0.112	0.111	0.139	0.197	0.201	0.203	0.129
VML164	0.232	0.126	0.125	0.095	0.157	0.217	0.177	0.151
VML165	0.117	0.145	0.137	0.199	0.276	0.253	0.204	0.084
VML168	0.203	0.127	0.130	0.124	0.177	0.240	0.212	0.139
VML170	0.164	0.156	0.141	0.162	0.230	0.282	0.195	0.116
VML171	0.090	0.187	0.173	0.243	0.316	0.306	0.237	0.098
VML173	0.338	0.152	0.149	0.149	0.164	0.135	0.109	0.295
VML174	0.161	0.172	0.165	0.180	0.249	0.289	0.238	0.080
VML175	0.265	0.142	0.146	0.103	0.156	0.205	0.226	0.177
VML176	0.237	0.117	0.112	0.165	0.206	0.199	0.074	0.213

VML178	0.274	0.093	0.105	0.094	0.080	0.185	0.125	0.238
VML179	0.153	0.181	0.172	0.192	0.276	0.282	0.252	0.057
VML180	0.136	0.164	0.159	0.187	0.260	0.281	0.224	0.068
VML183	0.203	0.167	0.153	0.148	0.239	0.250	0.217	0.080
VML184	0.210	0.180	0.170	0.163	0.245	0.264	0.238	0.090
VML188	0.297	0.119	0.122	0.068	0.110	0.153	0.102	0.234

	VML020	VML021	VML022	VML023	VML024	VML025	VML026	VML027
VML001	0.278	0.319	0.195	0.160	0.174	0.401	0.161	0.194
VML002	0.217	0.203	0.140	0.137	0.172	0.281	0.118	0.189
VML003	0.212	0.328	0.180	0.146	0.181	0.364	0.158	0.114
VML004	0.159	0.319	0.184	0.180	0.216	0.305	0.194	0.107
VML005	0.154	0.213	0.170	0.163	0.166	0.241	0.159	0.159
VML006	0.116	0.177	0.197	0.219	0.242	0.080	0.218	0.224
VML007	0.276	0.291	0.193	0.163	0.144	0.389	0.176	0.212
VML008	0.126	0.251	0.121	0.097	0.121	0.279	0.122	0.079
VML009	0.210	0.232	0.155	0.190	0.223	0.253	0.195	0.226
VML010	0.152	0.097	0.169	0.191	0.199	0.117	0.182	0.227
VML011	0.115	0.224	0.091	0.060	0.111	0.260	0.083	0.063
VML012	0.157	0.078	0.116	0.136	0.156	0.165	0.118	0.200
VML015	0.120	0.203	0.198	0.225	0.257	0.096	0.222	0.220
VML016	0.214	0.179	0.103	0.094	0.100	0.290	0.077	0.178
VML017	0.115	0.212	0.170	0.189	0.216	0.171	0.198	0.197
VML018	0.142	0.154	0.060	0.078	0.134	0.213	0.065	0.151
VML020		0.205	0.130	0.137	0.175	0.171	0.145	0.116
VML021	0.195		0.164	0.189	0.191	0.166	0.173	0.259
VML022	0.343	0.281		0.051	0.100	0.223	0.047	0.124
VML023	0.189	0.103	0.252		0.075	0.254	0.028	0.097
VML024	0.218	0.161	0.153	0.142		0.283	0.080	0.139
VML025	0.079	0.170	0.303	0.156	0.183		0.248	0.276
VML026	0.159	0.088	0.278	0.101	0.161	0.105		0.118
VML027	0.287	0.182	0.169	0.136	0.141	0.230	0.166	
VML028	0.110	0.151	0.301	0.168	0.199	0.099	0.113	0.240
VML030	0.148	0.059	0.284	0.109	0.152	0.132	0.084	0.201
VML031	0.178	0.158	0.297	0.157	0.210	0.115	0.081	0.189
VML032	0.280	0.205	0.162	0.177	0.131	0.214	0.169	0.082
VML033	0.289	0.251	0.136	0.199	0.142	0.230	0.214	0.113
VML034	0.252	0.172	0.165	0.151	0.060	0.214	0.179	0.139
VML036	0.178	0.076	0.314	0.153	0.198	0.166	0.107	0.231
VML039	0.259	0.217	0.177	0.163	0.130	0.249	0.234	0.187
VML040	0.256	0.165	0.195	0.169	0.114	0.197	0.144	0.125
VML041	0.103	0.128	0.350	0.149	0.223	0.124	0.125	0.265
VML042	0.189	0.162	0.178	0.131	0.086	0.136	0.122	0.126
VML043	0.166	0.091	0.262	0.106	0.157	0.115	0.034	0.158

VML044	0.173	0.055	0.296	0.122	0.174	0.161	0.100	0.211
VML046	0.279	0.146	0.185	0.163	0.111	0.252	0.191	0.158
VML047	0.115	0.112	0.310	0.150	0.189	0.109	0.096	0.238
VML048	0.158	0.095	0.356	0.151	0.225	0.158	0.115	0.258
VML049	0.127	0.099	0.341	0.139	0.209	0.133	0.108	0.252
VML050	0.188	0.117	0.216	0.112	0.110	0.130	0.074	0.124
VML051	0.195	0.159	0.202	0.139	0.091	0.133	0.114	0.136
VML052	0.240	0.224	0.124	0.178	0.094	0.198	0.198	0.144
VML053	0.160	0.130	0.211	0.117	0.085	0.145	0.135	0.178
VML054	0.360	0.270	0.069	0.243	0.175	0.314	0.269	0.138
VML055	0.222	0.187	0.300	0.171	0.230	0.162	0.126	0.185
VML056	0.134	0.118	0.282	0.128	0.135	0.101	0.095	0.206
VML057	0.085	0.134	0.308	0.115	0.175	0.071	0.097	0.222
VML058	0.183	0.146	0.204	0.096	0.120	0.132	0.106	0.119
VML059	0.176	0.086	0.232	0.093	0.136	0.150	0.095	0.156
VML061	0.117	0.154	0.324	0.159	0.205	0.059	0.079	0.229
VML063	0.135	0.094	0.256	0.087	0.144	0.110	0.078	0.174
VML064	0.133	0.104	0.300	0.136	0.189	0.133	0.107	0.227
VML065	0.124	0.113	0.351	0.155	0.225	0.124	0.105	0.259
VML066	0.249	0.162	0.139	0.145	0.067	0.200	0.154	0.096
VML067	0.155	0.122	0.204	0.089	0.094	0.120	0.101	0.142
VML068	0.228	0.121	0.177	0.107	0.079	0.178	0.117	0.087
VML069	0.121	0.193	0.406	0.219	0.281	0.125	0.153	0.315
VML070	0.129	0.093	0.280	0.080	0.157	0.097	0.062	0.181
VML073	0.203	0.091	0.223	0.060	0.129	0.175	0.113	0.130
VML074	0.070	0.190	0.349	0.191	0.235	0.070	0.136	0.277
VML075	0.150	0.103	0.338	0.154	0.197	0.133	0.099	0.245
VML076	0.237	0.192	0.242	0.155	0.174	0.163	0.127	0.122
VML080	0.229	0.124	0.190	0.148	0.121	0.197	0.140	0.151
VML081	0.112	0.146	0.315	0.153	0.171	0.120	0.138	0.251
VML083	0.110	0.201	0.414	0.226	0.273	0.141	0.180	0.334
VML084	0.126	0.144	0.296	0.172	0.174	0.148	0.156	0.260
VML085	0.223	0.189	0.201	0.130	0.156	0.165	0.139	0.105
VML086	0.138	0.214	0.369	0.195	0.268	0.109	0.148	0.272
VML087	0.173	0.065	0.264	0.098	0.136	0.129	0.055	0.163
VML088	0.081	0.182	0.345	0.191	0.233	0.131	0.170	0.294
VML089	0.168	0.170	0.254	0.150	0.179	0.109	0.102	0.173
VML090	0.218	0.158	0.206	0.088	0.138	0.191	0.159	0.132
VML091	0.163	0.089	0.281	0.145	0.156	0.130	0.078	0.204
VML092	0.226	0.127	0.226	0.123	0.162	0.171	0.094	0.112
VML093	0.207	0.131	0.183	0.103	0.114	0.161	0.110	0.100
VML094	0.129	0.078	0.261	0.087	0.132	0.101	0.064	0.179
VML095	0.122	0.130	0.268	0.094	0.150	0.069	0.070	0.174
VML096	0.207	0.063	0.250	0.128	0.139	0.186	0.117	0.182

VML097	0.202	0.090	0.202	0.099	0.087	0.155	0.088	0.117
VML099	0.133	0.085	0.308	0.128	0.190	0.125	0.086	0.223
VML100	0.216	0.181	0.170	0.125	0.107	0.177	0.163	0.125
VML102	0.232	0.212	0.151	0.152	0.111	0.183	0.177	0.120
VML104	0.197	0.182	0.239	0.132	0.163	0.131	0.117	0.137
VML106	0.279	0.178	0.142	0.134	0.095	0.233	0.182	0.075
VML107	0.224	0.095	0.205	0.070	0.109	0.186	0.115	0.101
VML110	0.261	0.153	0.158	0.125	0.078	0.213	0.156	0.081
VML111	0.169	0.104	0.291	0.151	0.163	0.177	0.147	0.239
VML112	0.189	0.036	0.263	0.090	0.144	0.171	0.102	0.176
VML113	0.180	0.214	0.289	0.163	0.208	0.114	0.137	0.192
VML114	0.238	0.174	0.136	0.138	0.074	0.192	0.159	0.099
VML116	0.137	0.094	0.303	0.108	0.189	0.136	0.105	0.218
VML117	0.288	0.190	0.155	0.141	0.129	0.232	0.175	0.027
VML118	0.368	0.269	0.235	0.262	0.192	0.352	0.313	0.256
VML119	0.222	0.166	0.402	0.216	0.257	0.217	0.185	0.308
VML120	0.082	0.160	0.312	0.158	0.204	0.097	0.130	0.250
VML121	0.094	0.134	0.298	0.132	0.179	0.045	0.071	0.212
VML122	0.162	0.099	0.285	0.113	0.176	0.109	0.033	0.174
VML123	0.230	0.132	0.179	0.086	0.097	0.186	0.132	0.084
VML124	0.146	0.156	0.396	0.212	0.264	0.172	0.167	0.319
VML125	0.263	0.216	0.404	0.260	0.282	0.296	0.275	0.360
VML126	0.177	0.130	0.243	0.115	0.148	0.120	0.078	0.147
VML127	0.182	0.163	0.183	0.145	0.059	0.143	0.141	0.160
VML128	0.082	0.124	0.331	0.136	0.199	0.099	0.112	0.251
VML129	0.175	0.085	0.237	0.116	0.128	0.161	0.116	0.182
VML130	0.083	0.155	0.350	0.180	0.226	0.086	0.118	0.273
VML131	0.308	0.157	0.190	0.187	0.147	0.274	0.199	0.155
VML132	0.135	0.142	0.246	0.103	0.129	0.080	0.087	0.164
VML133	0.210	0.168	0.365	0.220	0.238	0.233	0.212	0.314
VML134	0.382	0.300	0.218	0.253	0.234	0.315	0.271	0.145
VML136	0.119	0.182	0.260	0.158	0.146	0.142	0.178	0.241
VML137	0.153	0.152	0.402	0.196	0.270	0.181	0.169	0.314
VML138	0.278	0.138	0.213	0.150	0.165	0.223	0.135	0.096
VML139	0.127	0.142	0.272	0.084	0.164	0.117	0.122	0.192
VML140	0.452	0.365	0.130	0.342	0.241	0.412	0.375	0.248
VML141	0.263	0.208	0.273	0.182	0.226	0.195	0.149	0.151
VML143	0.178	0.113	0.211	0.091	0.093	0.155	0.122	0.152
VML144	0.473	0.400	0.204	0.358	0.284	0.413	0.381	0.240
VML145	0.110	0.144	0.353	0.160	0.236	0.134	0.139	0.273
VML146	0.141	0.095	0.270	0.075	0.151	0.094	0.040	0.164
VML147	0.235	0.230	0.269	0.196	0.210	0.160	0.154	0.173
VML148	0.081	0.168	0.305	0.158	0.202	0.086	0.129	0.244
VML150	0.140	0.076	0.256	0.084	0.132	0.108	0.060	0.170

VML151	0.209	0.123	0.176	0.105	0.101	0.175	0.127	0.119
VML152	0.170	0.266	0.459	0.289	0.350	0.188	0.226	0.379
VML153	0.138	0.206	0.420	0.235	0.303	0.154	0.175	0.332
VML154	0.292	0.245	0.173	0.177	0.160	0.237	0.214	0.107
VML155	0.197	0.190	0.257	0.153	0.160	0.127	0.126	0.162
VML156	0.173	0.096	0.353	0.148	0.220	0.164	0.115	0.250
VML157	0.164	0.079	0.262	0.129	0.138	0.141	0.092	0.194
VML158	0.166	0.288	0.485	0.312	0.366	0.207	0.257	0.413
VML159	0.185	0.109	0.267	0.091	0.170	0.183	0.142	0.192
VML160	0.183	0.132	0.177	0.120	0.075	0.142	0.112	0.132
VML161	0.147	0.170	0.285	0.140	0.173	0.096	0.114	0.199
VML162	0.184	0.126	0.246	0.105	0.171	0.148	0.099	0.153
VML164	0.139	0.159	0.232	0.128	0.135	0.082	0.099	0.166
VML165	0.179	0.058	0.292	0.120	0.177	0.176	0.119	0.214
VML168	0.123	0.151	0.278	0.107	0.167	0.072	0.091	0.184
VML170	0.106	0.142	0.294	0.126	0.155	0.088	0.111	0.220
VML171	0.186	0.107	0.337	0.150	0.213	0.197	0.156	0.257
VML173	0.329	0.237	0.108	0.201	0.119	0.286	0.244	0.126
VML174	0.081	0.137	0.319	0.139	0.198	0.063	0.090	0.233
VML175	0.183	0.178	0.260	0.139	0.181	0.117	0.108	0.158
VML176	0.249	0.170	0.154	0.156	0.097	0.233	0.199	0.165
VML178	0.225	0.208	0.170	0.136	0.105	0.187	0.185	0.133
VML179	0.130	0.103	0.337	0.139	0.207	0.106	0.074	0.234
VML180	0.098	0.112	0.308	0.136	0.188	0.098	0.096	0.233
VML183	0.134	0.118	0.287	0.143	0.172	0.082	0.053	0.200
VML184	0.128	0.139	0.302	0.156	0.195	0.078	0.071	0.215
VML188	0.238	0.200	0.127	0.165	0.077	0.189	0.175	0.122

	VML028	VML030	VML031	VML032	VML033	VML034	VML036	VML039
VML001	0.311	0.191	0.157	0.127	0.283	0.186	0.085	0.253
VML002	0.221	0.209	0.087	0.192	0.274	0.194	0.262	0.175
VML003	0.250	0.123	0.159	0.056	0.183	0.126	0.154	0.209
VML004	0.217	0.101	0.214	0.179	0.112	0.143	0.299	0.176
VML005	0.079	0.197	0.125	0.197	0.168	0.149	0.328	0.129
VML006	0.172	0.271	0.243	0.320	0.267	0.215	0.438	0.124
VML007	0.346	0.205	0.213	0.225	0.315	0.195	0.191	0.245
VML008	0.228	0.112	0.174	0.169	0.186	0.064	0.249	0.126
VML009	0.340	0.211	0.267	0.322	0.327	0.235	0.365	0.193
VML010	0.169	0.267	0.195	0.307	0.283	0.213	0.412	0.115
VML011	0.210	0.117	0.136	0.152	0.196	0.065	0.239	0.102
VML012	0.188	0.240	0.135	0.260	0.287	0.184	0.347	0.105
VML015	0.174	0.263	0.244	0.313	0.257	0.221	0.437	0.134
VML016	0.242	0.187	0.079	0.192	0.274	0.164	0.235	0.162
VML017	0.258	0.233	0.260	0.306	0.280	0.186	0.392	0.134

VML018	0.231	0.186	0.129	0.213	0.273	0.144	0.278	0.106
VML020	0.156	0.172	0.182	0.222	0.187	0.115	0.341	0.062
VML021	0.243	0.290	0.198	0.327	0.344	0.239	0.398	0.155
VML022	0.218	0.143	0.118	0.197	0.235	0.115	0.271	0.087
VML023	0.209	0.127	0.094	0.158	0.217	0.086	0.232	0.099
VML024	0.217	0.150	0.106	0.178	0.225	0.105	0.238	0.131
VML025	0.234	0.320	0.279	0.370	0.337	0.271	0.477	0.169
VML026	0.207	0.145	0.080	0.166	0.231	0.103	0.238	0.102
VML027	0.188	0.089	0.145	0.130	0.141	0.063	0.245	0.117
VML028		0.239	0.178	0.226	0.176	0.176	0.367	0.154
VML030	0.124		0.173	0.149	0.150	0.112	0.232	0.166
VML031	0.120	0.154		0.143	0.223	0.129	0.230	0.141
VML032	0.237	0.212	0.182		0.176	0.130	0.155	0.215
VML033	0.263	0.248	0.219	0.080		0.152	0.312	0.204
VML034	0.232	0.166	0.236	0.136	0.154		0.241	0.108
VML036	0.120	0.068	0.162	0.243	0.289	0.209		0.323
VML039	0.249	0.211	0.281	0.221	0.202	0.139	0.253	
VML040	0.214	0.166	0.183	0.085	0.147	0.103	0.192	0.223
VML041	0.109	0.099	0.162	0.280	0.305	0.249	0.116	0.255
VML042	0.157	0.144	0.149	0.105	0.119	0.110	0.182	0.170
VML043	0.104	0.086	0.080	0.163	0.206	0.175	0.100	0.227
VML044	0.125	0.050	0.167	0.230	0.272	0.184	0.041	0.219
VML046	0.238	0.159	0.253	0.180	0.212	0.091	0.183	0.154
VML047	0.057	0.081	0.137	0.240	0.274	0.214	0.073	0.243
VML048	0.132	0.083	0.171	0.274	0.316	0.237	0.070	0.270
VML049	0.114	0.069	0.164	0.265	0.300	0.225	0.077	0.250
VML050	0.139	0.110	0.117	0.112	0.159	0.123	0.138	0.200
VML051	0.173	0.143	0.149	0.098	0.130	0.107	0.184	0.199
VML052	0.222	0.215	0.215	0.126	0.091	0.136	0.259	0.149
VML053	0.142	0.102	0.190	0.185	0.197	0.110	0.145	0.128
VML054	0.305	0.283	0.286	0.149	0.142	0.176	0.305	0.196
VML055	0.174	0.197	0.074	0.187	0.217	0.261	0.213	0.293
VML056	0.146	0.083	0.158	0.195	0.225	0.154	0.137	0.219
VML057	0.110	0.092	0.142	0.227	0.246	0.195	0.135	0.219
VML058	0.148	0.145	0.122	0.131	0.141	0.157	0.183	0.172
VML059	0.113	0.092	0.138	0.182	0.211	0.164	0.110	0.173
VML061	0.100	0.127	0.086	0.215	0.248	0.228	0.144	0.279
VML063	0.098	0.080	0.119	0.192	0.214	0.177	0.117	0.188
VML064	0.073	0.078	0.148	0.243	0.273	0.211	0.070	0.223
VML065	0.103	0.090	0.141	0.268	0.303	0.249	0.092	0.275
VML066	0.207	0.164	0.194	0.085	0.118	0.067	0.196	0.159
VML067	0.124	0.105	0.140	0.152	0.164	0.131	0.149	0.147
VML068	0.189	0.133	0.162	0.095	0.135	0.096	0.172	0.170
VML069	0.131	0.164	0.156	0.310	0.341	0.307	0.165	0.339

VML070	0.108	0.080	0.111	0.196	0.223	0.185	0.121	0.206
VML073	0.160	0.108	0.163	0.174	0.199	0.147	0.140	0.148
VML074	0.077	0.152	0.134	0.269	0.288	0.265	0.163	0.284
VML075	0.144	0.074	0.164	0.243	0.287	0.206	0.092	0.269
VML076	0.205	0.195	0.115	0.099	0.132	0.191	0.228	0.256
VML080	0.158	0.130	0.182	0.162	0.200	0.133	0.132	0.177
VML081	0.146	0.096	0.199	0.249	0.271	0.181	0.138	0.221
VML083	0.164	0.160	0.213	0.328	0.356	0.291	0.174	0.325
VML084	0.113	0.102	0.208	0.263	0.285	0.195	0.114	0.207
VML085	0.178	0.191	0.130	0.119	0.123	0.182	0.221	0.196
VML086	0.138	0.196	0.114	0.271	0.287	0.300	0.212	0.314
VML087	0.144	0.066	0.126	0.167	0.211	0.153	0.109	0.219
VML088	0.092	0.145	0.190	0.302	0.314	0.269	0.155	0.253
VML089	0.111	0.161	0.063	0.165	0.184	0.216	0.180	0.242
VML090	0.193	0.165	0.190	0.180	0.176	0.165	0.207	0.123
VML091	0.114	0.067	0.139	0.195	0.243	0.169	0.070	0.240
VML092	0.152	0.146	0.100	0.130	0.177	0.181	0.154	0.224
VML093	0.148	0.134	0.135	0.122	0.147	0.136	0.158	0.164
VML094	0.099	0.053	0.127	0.189	0.218	0.159	0.098	0.190
VML095	0.112	0.110	0.100	0.177	0.196	0.180	0.153	0.208
VML096	0.152	0.076	0.183	0.199	0.244	0.145	0.078	0.195
VML097	0.157	0.097	0.145	0.122	0.166	0.104	0.133	0.177
VML099	0.080	0.064	0.132	0.237	0.272	0.212	0.060	0.235
VML100	0.199	0.178	0.183	0.142	0.124	0.148	0.227	0.140
VML102	0.211	0.207	0.189	0.116	0.083	0.146	0.251	0.154
VML104	0.172	0.180	0.105	0.131	0.142	0.193	0.217	0.226
VML106	0.247	0.193	0.223	0.110	0.122	0.095	0.234	0.136
VML107	0.183	0.120	0.169	0.144	0.176	0.122	0.156	0.153
VML110	0.225	0.168	0.204	0.098	0.132	0.075	0.206	0.152
VML111	0.147	0.076	0.215	0.252	0.284	0.172	0.088	0.199
VML112	0.150	0.058	0.172	0.205	0.242	0.156	0.087	0.185
VML113	0.169	0.202	0.105	0.180	0.186	0.237	0.235	0.263
VML114	0.200	0.169	0.194	0.098	0.110	0.078	0.203	0.138
VML116	0.102	0.084	0.148	0.243	0.269	0.215	0.100	0.212
VML117	0.245	0.206	0.202	0.077	0.103	0.123	0.239	0.174
VML118	0.360	0.272	0.380	0.274	0.280	0.156	0.309	0.186
VML119	0.231	0.151	0.252	0.308	0.356	0.250	0.159	0.320
VML120	0.052	0.126	0.145	0.257	0.272	0.238	0.138	0.233
VML121	0.076	0.099	0.092	0.207	0.232	0.206	0.127	0.241
VML122	0.109	0.103	0.064	0.179	0.221	0.201	0.119	0.246
VML123	0.195	0.148	0.173	0.123	0.142	0.119	0.190	0.143
VML124	0.140	0.129	0.209	0.325	0.362	0.282	0.111	0.309
VML125	0.269	0.199	0.345	0.382	0.411	0.278	0.199	0.282
VML126	0.135	0.135	0.087	0.146	0.177	0.184	0.165	0.222

VML127	0.166	0.138	0.184	0.137	0.149	0.087	0.181	0.157
VML128	0.101	0.086	0.155	0.260	0.282	0.226	0.117	0.238
VML129	0.121	0.075	0.173	0.200	0.231	0.148	0.090	0.168
VML130	0.081	0.114	0.138	0.267	0.295	0.251	0.120	0.283
VML131	0.247	0.184	0.251	0.177	0.224	0.133	0.189	0.197
VML132	0.128	0.124	0.115	0.159	0.173	0.165	0.171	0.195
VML133	0.205	0.141	0.279	0.325	0.360	0.236	0.134	0.265
VML134	0.348	0.320	0.272	0.139	0.142	0.237	0.356	0.289
VML136	0.148	0.142	0.220	0.247	0.242	0.180	0.186	0.152
VML137	0.155	0.129	0.213	0.331	0.364	0.286	0.120	0.301
VML138	0.207	0.173	0.157	0.120	0.181	0.171	0.178	0.234
VML139	0.129	0.118	0.155	0.217	0.221	0.191	0.162	0.170
VML140	0.416	0.374	0.406	0.244	0.227	0.227	0.403	0.253
VML141	0.210	0.223	0.105	0.150	0.183	0.249	0.240	0.292
VML143	0.150	0.095	0.181	0.171	0.191	0.103	0.136	0.128
VML144	0.445	0.410	0.393	0.217	0.192	0.271	0.447	0.325
VML145	0.097	0.121	0.165	0.291	0.313	0.264	0.125	0.258
VML146	0.109	0.084	0.096	0.175	0.206	0.172	0.121	0.207
VML147	0.206	0.226	0.113	0.140	0.158	0.236	0.255	0.290
VML148	0.060	0.138	0.133	0.248	0.259	0.240	0.156	0.235
VML150	0.102	0.066	0.120	0.182	0.214	0.162	0.105	0.192
VML151	0.151	0.129	0.164	0.146	0.167	0.128	0.153	0.138
VML152	0.168	0.242	0.205	0.374	0.400	0.382	0.229	0.396
VML153	0.132	0.180	0.172	0.334	0.364	0.330	0.167	0.349
VML154	0.273	0.252	0.222	0.117	0.081	0.177	0.297	0.194
VML155	0.196	0.178	0.131	0.133	0.149	0.183	0.225	0.247
VML156	0.154	0.080	0.178	0.264	0.308	0.223	0.077	0.270
VML157	0.120	0.048	0.157	0.196	0.236	0.149	0.066	0.209
VML158	0.193	0.253	0.251	0.408	0.430	0.396	0.243	0.408
VML159	0.151	0.112	0.190	0.236	0.253	0.189	0.135	0.157
VML160	0.139	0.118	0.151	0.126	0.149	0.106	0.151	0.156
VML161	0.154	0.158	0.123	0.190	0.204	0.211	0.202	0.240
VML162	0.113	0.133	0.108	0.181	0.207	0.199	0.143	0.201
VML164	0.111	0.140	0.104	0.154	0.166	0.175	0.175	0.202
VML165	0.136	0.064	0.179	0.241	0.276	0.193	0.068	0.207
VML168	0.124	0.131	0.103	0.188	0.199	0.199	0.174	0.217
VML170	0.130	0.098	0.166	0.216	0.237	0.168	0.143	0.208
VML171	0.165	0.092	0.221	0.284	0.318	0.215	0.088	0.230
VML173	0.300	0.247	0.285	0.136	0.133	0.101	0.285	0.150
VML174	0.072	0.101	0.110	0.235	0.258	0.226	0.123	0.248
VML175	0.154	0.173	0.078	0.152	0.168	0.213	0.205	0.244
VML176	0.214	0.168	0.249	0.190	0.195	0.107	0.198	0.088
VML178	0.225	0.197	0.212	0.144	0.109	0.131	0.251	0.125
VML179	0.112	0.080	0.117	0.238	0.276	0.227	0.096	0.271

VML180	0.066	0.080	0.130	0.241	0.267	0.219	0.097	0.233
VML183	0.084	0.094	0.081	0.187	0.227	0.196	0.106	0.254
VML184	0.066	0.117	0.066	0.205	0.240	0.223	0.122	0.267
VML188	0.213	0.194	0.196	0.097	0.081	0.117	0.238	0.161

	VML040	VML041	VML042	VML043	VML044	VML046	VML047	VML048
VML001	0.103	0.218	0.259	0.285	0.109	0.233	0.315	0.336
VML002	0.207	0.150	0.290	0.186	0.160	0.251	0.335	0.200
VML003	0.119	0.198	0.167	0.277	0.110	0.165	0.305	0.346
VML004	0.230	0.181	0.121	0.277	0.204	0.125	0.322	0.342
VML005	0.239	0.134	0.218	0.209	0.215	0.259	0.394	0.253
VML006	0.319	0.226	0.265	0.152	0.277	0.271	0.334	0.204
VML007	0.118	0.215	0.251	0.281	0.147	0.230	0.290	0.333
VML008	0.127	0.161	0.109	0.208	0.115	0.120	0.245	0.290
VML009	0.274	0.214	0.272	0.213	0.229	0.163	0.199	0.244
VML010	0.297	0.180	0.289	0.127	0.258	0.288	0.355	0.145
VML011	0.114	0.141	0.144	0.174	0.079	0.129	0.238	0.253
VML012	0.242	0.159	0.284	0.079	0.196	0.260	0.311	0.108
VML015	0.328	0.229	0.267	0.167	0.280	0.259	0.334	0.215
VML016	0.165	0.123	0.266	0.181	0.132	0.232	0.315	0.207
VML017	0.265	0.236	0.219	0.163	0.229	0.195	0.220	0.241
VML018	0.174	0.157	0.241	0.096	0.115	0.182	0.221	0.161
VML020	0.221	0.170	0.163	0.150	0.182	0.170	0.270	0.232
VML021	0.289	0.196	0.332	0.109	0.249	0.305	0.333	0.090
VML022	0.169	0.115	0.207	0.133	0.119	0.155	0.245	0.191
VML023	0.122	0.110	0.185	0.156	0.079	0.157	0.258	0.220
VML024	0.132	0.106	0.198	0.193	0.125	0.203	0.310	0.246
VML025	0.359	0.267	0.330	0.141	0.309	0.306	0.327	0.165
VML026	0.136	0.112	0.208	0.143	0.088	0.175	0.264	0.201
VML027	0.140	0.138	0.107	0.213	0.115	0.124	0.279	0.288
VML028	0.272	0.195	0.230	0.225	0.249	0.291	0.412	0.278
VML030	0.161	0.129	0.122	0.262	0.145	0.104	0.297	0.323
VML031	0.152	0.118	0.234	0.182	0.123	0.232	0.335	0.226
VML032	0.121	0.197	0.180	0.283	0.127	0.210	0.345	0.350
VML033	0.243	0.198	0.114	0.316	0.239	0.214	0.401	0.384
VML034	0.128	0.141	0.116	0.199	0.112	0.153	0.286	0.280
VML036	0.142	0.280	0.287	0.364	0.175	0.276	0.364	0.418
VML039	0.203	0.127	0.186	0.120	0.162	0.181	0.278	0.192
VML040		0.192	0.193	0.251	0.069	0.198	0.285	0.320
VML041	0.251		0.205	0.211	0.169	0.198	0.342	0.242
VML042	0.099	0.204		0.293	0.197	0.149	0.321	0.374
VML043	0.138	0.134	0.110		0.199	0.254	0.256	0.102
VML044	0.182	0.110	0.167	0.097		0.165	0.243	0.267
VML046	0.139	0.248	0.158	0.180	0.160		0.214	0.322

VML047	0.200	0.085	0.161	0.094	0.077	0.212		0.316
VML048	0.228	0.081	0.210	0.124	0.068	0.224	0.081	
VML049	0.223	0.056	0.192	0.117	0.069	0.219	0.065	0.034
VML050	0.082	0.179	0.062	0.064	0.126	0.150	0.130	0.167
VML051	0.076	0.208	0.052	0.114	0.170	0.166	0.167	0.206
VML052	0.163	0.262	0.102	0.192	0.238	0.188	0.234	0.284
VML053	0.153	0.160	0.097	0.127	0.119	0.129	0.127	0.168
VML054	0.187	0.352	0.185	0.251	0.288	0.179	0.311	0.350
VML055	0.213	0.199	0.181	0.130	0.211	0.277	0.192	0.217
VML056	0.153	0.133	0.130	0.113	0.121	0.187	0.116	0.132
VML057	0.199	0.087	0.144	0.109	0.119	0.222	0.092	0.113
VML058	0.153	0.176	0.087	0.103	0.164	0.184	0.159	0.198
VML059	0.167	0.133	0.122	0.080	0.090	0.149	0.106	0.139
VML061	0.191	0.124	0.154	0.095	0.147	0.256	0.099	0.133
VML063	0.179	0.099	0.122	0.075	0.099	0.181	0.093	0.126
VML064	0.209	0.082	0.163	0.096	0.067	0.198	0.047	0.088
VML065	0.235	0.045	0.201	0.114	0.097	0.244	0.071	0.061
VML066	0.071	0.244	0.076	0.143	0.178	0.103	0.200	0.234
VML067	0.145	0.149	0.072	0.092	0.127	0.153	0.125	0.169
VML068	0.089	0.207	0.087	0.112	0.152	0.115	0.178	0.201
VML069	0.281	0.105	0.243	0.166	0.176	0.319	0.124	0.129
VML070	0.181	0.089	0.133	0.075	0.102	0.197	0.095	0.112
VML073	0.166	0.157	0.129	0.105	0.111	0.133	0.146	0.159
VML074	0.247	0.108	0.186	0.140	0.166	0.288	0.097	0.147
VML075	0.189	0.112	0.183	0.117	0.091	0.212	0.095	0.072
VML076	0.138	0.235	0.123	0.132	0.218	0.234	0.213	0.240
VML080	0.128	0.203	0.115	0.117	0.122	0.103	0.147	0.186
VML081	0.200	0.123	0.164	0.148	0.121	0.207	0.110	0.123
VML083	0.285	0.116	0.251	0.196	0.176	0.309	0.137	0.129
VML084	0.217	0.122	0.171	0.150	0.103	0.192	0.085	0.127
VML085	0.164	0.221	0.103	0.130	0.205	0.214	0.197	0.238
VML086	0.273	0.143	0.215	0.160	0.211	0.325	0.161	0.187
VML087	0.132	0.134	0.125	0.070	0.096	0.160	0.116	0.121
VML088	0.278	0.081	0.211	0.169	0.152	0.271	0.100	0.142
VML089	0.180	0.170	0.119	0.094	0.176	0.238	0.142	0.195
VML090	0.200	0.192	0.140	0.155	0.177	0.174	0.195	0.216
VML091	0.138	0.137	0.135	0.076	0.074	0.169	0.076	0.104
VML092	0.137	0.193	0.119	0.076	0.148	0.178	0.156	0.186
VML093	0.127	0.189	0.075	0.089	0.142	0.144	0.152	0.192
VML094	0.160	0.097	0.116	0.068	0.076	0.167	0.075	0.105
VML095	0.171	0.120	0.110	0.083	0.136	0.212	0.113	0.146
VML096	0.149	0.159	0.148	0.107	0.059	0.107	0.116	0.124
VML097	0.091	0.177	0.085	0.082	0.114	0.112	0.140	0.165
VML099	0.202	0.068	0.166	0.083	0.058	0.201	0.043	0.066

VML100	0.174	0.215	0.110	0.159	0.203	0.181	0.207	0.243
VML102	0.162	0.247	0.096	0.173	0.229	0.197	0.225	0.269
VML104	0.166	0.201	0.114	0.123	0.203	0.234	0.187	0.222
VML106	0.133	0.261	0.127	0.178	0.206	0.121	0.238	0.258
VML107	0.137	0.184	0.120	0.111	0.128	0.114	0.167	0.177
VML110	0.099	0.243	0.109	0.154	0.181	0.105	0.212	0.232
VML111	0.197	0.132	0.175	0.143	0.067	0.151	0.102	0.108
VML112	0.168	0.128	0.152	0.100	0.055	0.128	0.114	0.108
VML113	0.208	0.200	0.149	0.146	0.225	0.283	0.192	0.229
VML114	0.099	0.240	0.062	0.145	0.183	0.121	0.198	0.237
VML116	0.221	0.063	0.173	0.105	0.083	0.204	0.083	0.092
VML117	0.121	0.273	0.120	0.168	0.217	0.153	0.242	0.265
VML118	0.242	0.352	0.261	0.310	0.281	0.147	0.331	0.334
VML119	0.244	0.184	0.259	0.203	0.155	0.251	0.178	0.127
VML120	0.238	0.085	0.168	0.126	0.132	0.247	0.074	0.133
VML121	0.185	0.098	0.131	0.078	0.124	0.229	0.078	0.123
VML122	0.165	0.124	0.137	0.048	0.115	0.210	0.103	0.124
VML123	0.138	0.204	0.108	0.129	0.163	0.133	0.189	0.209
VML124	0.276	0.092	0.250	0.175	0.121	0.271	0.098	0.074
VML125	0.322	0.220	0.309	0.276	0.185	0.245	0.222	0.191
VML126	0.156	0.160	0.116	0.085	0.154	0.204	0.142	0.175
VML127	0.109	0.203	0.054	0.135	0.163	0.144	0.160	0.207
VML128	0.230	0.033	0.180	0.122	0.106	0.234	0.076	0.087
VML129	0.163	0.138	0.128	0.101	0.067	0.124	0.097	0.129
VML130	0.232	0.079	0.187	0.123	0.129	0.263	0.068	0.105
VML131	0.141	0.270	0.177	0.185	0.174	0.063	0.225	0.238
VML132	0.160	0.145	0.096	0.099	0.153	0.204	0.132	0.170
VML133	0.260	0.174	0.250	0.212	0.127	0.212	0.155	0.135
VML134	0.213	0.375	0.228	0.272	0.338	0.276	0.353	0.374
VML136	0.226	0.150	0.151	0.175	0.164	0.204	0.146	0.190
VML137	0.287	0.076	0.257	0.178	0.122	0.273	0.114	0.067
VML138	0.123	0.238	0.147	0.122	0.170	0.155	0.200	0.217
VML139	0.212	0.112	0.139	0.122	0.138	0.209	0.129	0.154
VML140	0.265	0.453	0.283	0.363	0.383	0.237	0.413	0.446
VML141	0.193	0.245	0.175	0.148	0.236	0.270	0.227	0.253
VML143	0.141	0.162	0.094	0.112	0.107	0.114	0.131	0.160
VML144	0.271	0.482	0.296	0.376	0.429	0.316	0.448	0.478
VML145	0.266	0.037	0.212	0.142	0.120	0.259	0.086	0.096
VML146	0.159	0.111	0.115	0.055	0.103	0.191	0.097	0.119
VML147	0.181	0.248	0.151	0.159	0.250	0.281	0.226	0.264
VML148	0.237	0.097	0.163	0.127	0.150	0.255	0.093	0.151
VML150	0.159	0.105	0.117	0.066	0.083	0.168	0.083	0.112
VML151	0.142	0.185	0.094	0.109	0.132	0.121	0.150	0.189
VML152	0.354	0.167	0.306	0.233	0.245	0.389	0.183	0.201

VML153	0.306	0.108	0.264	0.182	0.183	0.332	0.130	0.137
VML154	0.185	0.295	0.152	0.214	0.274	0.227	0.282	0.312
VML155	0.151	0.211	0.121	0.140	0.213	0.238	0.199	0.227
VML156	0.212	0.108	0.204	0.125	0.074	0.213	0.103	0.043
VML157	0.141	0.134	0.126	0.083	0.058	0.141	0.082	0.108
VML158	0.377	0.177	0.328	0.263	0.258	0.404	0.198	0.210
VML159	0.220	0.128	0.170	0.135	0.104	0.168	0.135	0.142
VML160	0.107	0.183	0.046	0.097	0.135	0.129	0.137	0.186
VML161	0.198	0.156	0.148	0.134	0.187	0.249	0.161	0.189
VML162	0.186	0.149	0.131	0.083	0.131	0.196	0.126	0.162
VML164	0.160	0.158	0.084	0.098	0.163	0.212	0.131	0.186
VML165	0.203	0.106	0.179	0.114	0.044	0.159	0.097	0.088
VML168	0.191	0.127	0.126	0.103	0.159	0.233	0.133	0.164
VML170	0.178	0.125	0.131	0.122	0.124	0.205	0.106	0.131
VML171	0.234	0.120	0.213	0.155	0.068	0.188	0.117	0.081
VML173	0.154	0.323	0.164	0.238	0.260	0.134	0.292	0.317
VML174	0.212	0.070	0.156	0.096	0.121	0.242	0.070	0.109
VML175	0.179	0.184	0.126	0.112	0.196	0.247	0.173	0.210
VML176	0.173	0.234	0.139	0.184	0.172	0.085	0.204	0.235
VML178	0.174	0.239	0.109	0.184	0.224	0.187	0.230	0.263
VML179	0.203	0.076	0.178	0.090	0.099	0.232	0.081	0.072
VML180	0.214	0.052	0.163	0.097	0.094	0.219	0.051	0.093
VML183	0.155	0.124	0.125	0.054	0.113	0.212	0.076	0.122
VML184	0.184	0.120	0.142	0.070	0.130	0.238	0.079	0.131
VML188	0.129	0.251	0.084	0.169	0.219	0.167	0.220	0.265

	VML049	VML050	VML051	VML052	VML053	VML054	VML055	VML056
VML001	0.280	0.309	0.346	0.319	0.308	0.210	0.198	0.235
VML002	0.190	0.281	0.256	0.234	0.214	0.247	0.158	0.174
VML003	0.288	0.264	0.326	0.264	0.234	0.196	0.212	0.177
VML004	0.278	0.236	0.300	0.250	0.132	0.233	0.271	0.159
VML005	0.209	0.237	0.235	0.113	0.137	0.239	0.139	0.163
VML006	0.185	0.147	0.111	0.269	0.133	0.240	0.283	0.159
VML007	0.237	0.276	0.317	0.384	0.323	0.158	0.242	0.262
VML008	0.201	0.147	0.228	0.292	0.184	0.084	0.226	0.130
VML009	0.171	0.210	0.222	0.408	0.223	0.217	0.340	0.207
VML010	0.122	0.193	0.117	0.248	0.158	0.240	0.226	0.175
VML011	0.178	0.155	0.210	0.272	0.171	0.098	0.201	0.098
VML012	0.095	0.194	0.128	0.249	0.178	0.203	0.181	0.144
VML015	0.207	0.171	0.146	0.259	0.107	0.261	0.293	0.150
VML016	0.150	0.257	0.243	0.263	0.227	0.194	0.133	0.182
VML017	0.180	0.057	0.126	0.354	0.184	0.153	0.315	0.151
VML018	0.121	0.167	0.159	0.292	0.185	0.143	0.206	0.101
VML020	0.179	0.102	0.151	0.248	0.097	0.158	0.240	0.071

VML021	0.074	0.219	0.123	0.309	0.227	0.234	0.230	0.207
VML022	0.117	0.169	0.178	0.272	0.156	0.139	0.191	0.105
VML023	0.144	0.180	0.204	0.259	0.175	0.120	0.165	0.112
VML024	0.148	0.205	0.227	0.259	0.209	0.130	0.137	0.172
VML025	0.187	0.182	0.094	0.324	0.185	0.278	0.326	0.193
VML026	0.134	0.187	0.197	0.254	0.178	0.135	0.156	0.114
VML027	0.216	0.184	0.245	0.240	0.146	0.149	0.205	0.108
VML028	0.248	0.231	0.236	0.118	0.155	0.258	0.188	0.177
VML030	0.235	0.230	0.292	0.277	0.173	0.186	0.233	0.168
VML031	0.179	0.240	0.240	0.199	0.199	0.191	0.092	0.154
VML032	0.294	0.277	0.333	0.231	0.244	0.209	0.179	0.192
VML033	0.311	0.262	0.330	0.200	0.173	0.255	0.248	0.209
VML034	0.202	0.162	0.227	0.231	0.163	0.113	0.176	0.113
VML036	0.356	0.369	0.421	0.367	0.371	0.264	0.256	0.304
VML039	0.127	0.128	0.137	0.236	0.106	0.150	0.198	0.081
VML040	0.246	0.244	0.298	0.308	0.272	0.136	0.193	0.196
VML041	0.151	0.237	0.242	0.234	0.158	0.207	0.168	0.175
VML042	0.286	0.205	0.301	0.283	0.192	0.180	0.271	0.186
VML043	0.120	0.157	0.084	0.301	0.198	0.187	0.239	0.131
VML044	0.207	0.211	0.253	0.293	0.230	0.126	0.191	0.143
VML046	0.248	0.202	0.278	0.344	0.188	0.180	0.304	0.158
VML047	0.291	0.230	0.276	0.485	0.327	0.225	0.411	0.240
VML048	0.130	0.248	0.141	0.340	0.250	0.274	0.275	0.215
VML049		0.194	0.137	0.317	0.204	0.193	0.226	0.184
VML050	0.158		0.129	0.328	0.187	0.130	0.291	0.133
VML051	0.192	0.060		0.330	0.204	0.204	0.286	0.167
VML052	0.263	0.150	0.123		0.222	0.328	0.178	0.249
VML053	0.146	0.108	0.118	0.145		0.236	0.255	0.123
VML054	0.340	0.209	0.210	0.157	0.225		0.233	0.155
VML055	0.211	0.158	0.181	0.217	0.227	0.286		0.226
VML056	0.114	0.109	0.106	0.192	0.112	0.293	0.198	
VML057	0.085	0.130	0.145	0.212	0.119	0.314	0.187	0.082
VML058	0.181	0.094	0.106	0.111	0.121	0.204	0.124	0.140
VML059	0.127	0.100	0.144	0.171	0.096	0.225	0.163	0.138
VML061	0.121	0.124	0.143	0.229	0.170	0.325	0.144	0.108
VML063	0.106	0.105	0.137	0.173	0.101	0.257	0.146	0.111
VML064	0.071	0.137	0.180	0.232	0.120	0.297	0.196	0.138
VML065	0.049	0.166	0.200	0.268	0.170	0.350	0.183	0.128
VML066	0.223	0.088	0.086	0.113	0.117	0.137	0.216	0.161
VML067	0.148	0.081	0.098	0.120	0.066	0.211	0.166	0.112
VML068	0.192	0.075	0.085	0.124	0.117	0.171	0.174	0.133
VML069	0.120	0.213	0.236	0.311	0.230	0.409	0.201	0.168
VML070	0.094	0.108	0.136	0.189	0.118	0.279	0.138	0.093
VML073	0.148	0.112	0.148	0.168	0.105	0.212	0.176	0.148

VML074	0.125	0.173	0.193	0.253	0.179	0.357	0.188	0.149
VML075	0.071	0.141	0.165	0.261	0.156	0.338	0.214	0.081
VML076	0.231	0.113	0.110	0.163	0.199	0.231	0.109	0.171
VML080	0.180	0.100	0.139	0.172	0.105	0.179	0.216	0.169
VML081	0.099	0.151	0.157	0.231	0.113	0.328	0.249	0.078
VML083	0.115	0.227	0.239	0.318	0.216	0.424	0.263	0.148
VML084	0.105	0.162	0.184	0.231	0.097	0.309	0.259	0.132
VML085	0.224	0.115	0.128	0.124	0.164	0.191	0.125	0.188
VML086	0.172	0.200	0.218	0.265	0.230	0.370	0.133	0.187
VML087	0.115	0.082	0.109	0.190	0.123	0.261	0.156	0.074
VML088	0.114	0.204	0.228	0.258	0.162	0.357	0.229	0.170
VML089	0.180	0.111	0.134	0.166	0.164	0.251	0.087	0.160
VML090	0.199	0.150	0.166	0.137	0.131	0.204	0.183	0.181
VML091	0.100	0.090	0.125	0.218	0.120	0.279	0.194	0.091
VML092	0.183	0.085	0.132	0.175	0.159	0.202	0.114	0.171
VML093	0.180	0.073	0.109	0.130	0.108	0.170	0.157	0.156
VML094	0.086	0.089	0.120	0.179	0.085	0.264	0.165	0.077
VML095	0.126	0.098	0.112	0.167	0.121	0.272	0.129	0.092
VML096	0.124	0.112	0.153	0.211	0.104	0.242	0.221	0.133
VML097	0.156	0.053	0.083	0.145	0.096	0.197	0.171	0.108
VML099	0.052	0.131	0.173	0.235	0.130	0.304	0.179	0.121
VML100	0.223	0.138	0.131	0.077	0.133	0.183	0.175	0.170
VML102	0.250	0.137	0.118	0.047	0.149	0.167	0.185	0.186
VML104	0.208	0.116	0.116	0.140	0.173	0.237	0.093	0.157
VML106	0.248	0.138	0.134	0.115	0.149	0.135	0.222	0.190
VML107	0.170	0.101	0.129	0.154	0.115	0.192	0.176	0.144
VML110	0.224	0.108	0.107	0.123	0.132	0.151	0.212	0.162
VML111	0.097	0.153	0.181	0.238	0.098	0.295	0.261	0.126
VML112	0.103	0.118	0.157	0.208	0.105	0.256	0.200	0.120
VML113	0.213	0.150	0.149	0.183	0.201	0.290	0.105	0.172
VML114	0.223	0.094	0.092	0.100	0.107	0.136	0.217	0.168
VML116	0.074	0.148	0.186	0.226	0.130	0.300	0.177	0.136
VML117	0.258	0.126	0.130	0.132	0.172	0.129	0.201	0.205
VML118	0.326	0.271	0.261	0.257	0.224	0.248	0.398	0.275
VML119	0.139	0.218	0.234	0.333	0.221	0.401	0.298	0.150
VML120	0.106	0.160	0.187	0.224	0.137	0.319	0.189	0.147
VML121	0.101	0.110	0.133	0.204	0.134	0.301	0.146	0.096
VML122	0.118	0.095	0.133	0.203	0.152	0.276	0.100	0.116
VML123	0.198	0.107	0.120	0.118	0.122	0.172	0.171	0.154
VML124	0.074	0.216	0.247	0.319	0.199	0.398	0.259	0.162
VML125	0.190	0.289	0.312	0.362	0.223	0.410	0.388	0.239
VML126	0.164	0.095	0.115	0.153	0.148	0.241	0.088	0.127
VML127	0.188	0.088	0.064	0.109	0.071	0.203	0.220	0.113
VML128	0.056	0.159	0.182	0.239	0.138	0.337	0.195	0.105

VML129	0.117	0.109	0.147	0.187	0.071	0.236	0.211	0.130
VML130	0.084	0.163	0.188	0.261	0.167	0.355	0.195	0.125
VML131	0.240	0.158	0.185	0.210	0.167	0.167	0.272	0.218
VML132	0.150	0.095	0.095	0.139	0.116	0.254	0.135	0.094
VML133	0.134	0.225	0.250	0.317	0.175	0.370	0.331	0.181
VML134	0.368	0.236	0.220	0.200	0.297	0.198	0.240	0.298
VML136	0.159	0.172	0.173	0.175	0.083	0.285	0.253	0.141
VML137	0.066	0.223	0.257	0.324	0.204	0.402	0.256	0.170
VML138	0.219	0.111	0.150	0.191	0.184	0.179	0.161	0.197
VML139	0.128	0.140	0.160	0.181	0.111	0.276	0.177	0.134
VML140	0.436	0.312	0.294	0.236	0.307	0.143	0.408	0.370
VML141	0.248	0.156	0.175	0.205	0.239	0.252	0.062	0.220
VML143	0.143	0.093	0.116	0.153	0.041	0.214	0.216	0.119
VML144	0.469	0.323	0.293	0.251	0.359	0.201	0.380	0.385
VML145	0.075	0.190	0.223	0.268	0.169	0.355	0.201	0.159
VML146	0.104	0.083	0.116	0.182	0.117	0.266	0.131	0.091
VML147	0.253	0.149	0.141	0.180	0.228	0.265	0.102	0.196
VML148	0.125	0.159	0.182	0.211	0.144	0.314	0.171	0.149
VML150	0.097	0.088	0.121	0.174	0.094	0.257	0.152	0.087
VML151	0.176	0.094	0.126	0.131	0.089	0.169	0.183	0.158
VML152	0.194	0.282	0.308	0.365	0.294	0.462	0.242	0.251
VML153	0.130	0.235	0.265	0.331	0.244	0.420	0.216	0.201
VML154	0.297	0.179	0.159	0.109	0.210	0.173	0.200	0.229
VML155	0.212	0.121	0.095	0.155	0.181	0.263	0.136	0.131
VML156	0.058	0.160	0.197	0.285	0.168	0.345	0.227	0.125
VML157	0.099	0.091	0.128	0.206	0.091	0.261	0.208	0.097
VML158	0.200	0.308	0.330	0.391	0.303	0.493	0.296	0.261
VML159	0.127	0.157	0.194	0.209	0.115	0.262	0.210	0.166
VML160	0.170	0.061	0.077	0.114	0.069	0.183	0.185	0.124
VML161	0.172	0.141	0.136	0.172	0.168	0.295	0.123	0.118
VML162	0.151	0.112	0.157	0.180	0.138	0.233	0.126	0.167
VML164	0.165	0.093	0.098	0.129	0.120	0.241	0.130	0.121
VML165	0.084	0.146	0.188	0.236	0.122	0.286	0.213	0.139
VML168	0.143	0.120	0.129	0.171	0.141	0.283	0.122	0.112
VML170	0.107	0.122	0.127	0.203	0.103	0.304	0.215	0.069
VML171	0.081	0.182	0.220	0.282	0.147	0.332	0.266	0.156
VML173	0.306	0.187	0.171	0.136	0.186	0.111	0.289	0.239
VML174	0.084	0.136	0.162	0.225	0.143	0.322	0.161	0.111
VML175	0.195	0.120	0.129	0.162	0.179	0.257	0.071	0.157
VML176	0.220	0.157	0.168	0.147	0.096	0.164	0.270	0.194
VML178	0.242	0.150	0.128	0.076	0.136	0.188	0.214	0.176
VML179	0.064	0.138	0.169	0.251	0.163	0.333	0.164	0.101
VML180	0.068	0.140	0.171	0.223	0.128	0.311	0.173	0.115
VML183	0.111	0.087	0.119	0.206	0.139	0.286	0.143	0.098

VML184	0.119	0.111	0.144	0.217	0.157	0.300	0.130	0.124
VML188	0.248	0.124	0.097	0.040	0.137	0.150	0.200	0.173

	VML057	VML058	VML059	VML061	VML063	VML064	VML065	VML066
VML001	0.312	0.323	0.189	0.422	0.241	0.160	0.198	0.198
VML002	0.229	0.237	0.165	0.317	0.180	0.156	0.164	0.211
VML003	0.261	0.273	0.174	0.388	0.224	0.158	0.188	0.174
VML004	0.191	0.222	0.182	0.332	0.201	0.200	0.222	0.196
VML005	0.228	0.116	0.173	0.294	0.176	0.178	0.185	0.220
VML006	0.153	0.091	0.209	0.127	0.179	0.228	0.192	0.221
VML007	0.306	0.317	0.178	0.400	0.201	0.148	0.197	0.162
VML008	0.186	0.195	0.107	0.295	0.130	0.097	0.121	0.064
VML009	0.138	0.273	0.166	0.246	0.143	0.196	0.203	0.169
VML010	0.180	0.100	0.185	0.173	0.146	0.199	0.175	0.220
VML011	0.168	0.187	0.094	0.278	0.114	0.071	0.086	0.056
VML012	0.179	0.144	0.139	0.207	0.125	0.148	0.120	0.180
VML015	0.137	0.116	0.214	0.139	0.192	0.239	0.206	0.233
VML016	0.235	0.228	0.111	0.322	0.138	0.109	0.140	0.169
VML017	0.131	0.175	0.174	0.164	0.152	0.188	0.157	0.138
VML018	0.148	0.194	0.092	0.230	0.106	0.092	0.062	0.102
VML020	0.116	0.108	0.139	0.198	0.130	0.147	0.122	0.129
VML021	0.211	0.179	0.182	0.201	0.147	0.191	0.168	0.217
VML022	0.143	0.176	0.042	0.247	0.072	0.076	0.088	0.097
VML023	0.176	0.184	0.058	0.280	0.091	0.045	0.079	0.084
VML024	0.228	0.187	0.081	0.314	0.107	0.067	0.121	0.124
VML025	0.161	0.161	0.244	0.068	0.208	0.263	0.218	0.254
VML026	0.178	0.184	0.061	0.275	0.097	0.052	0.075	0.102
VML027	0.177	0.186	0.125	0.301	0.143	0.114	0.136	0.113
VML028	0.245	0.109	0.222	0.287	0.220	0.217	0.206	0.247
VML030	0.200	0.233	0.132	0.344	0.156	0.134	0.184	0.148
VML031	0.233	0.192	0.125	0.318	0.153	0.105	0.126	0.170
VML032	0.285	0.265	0.191	0.400	0.236	0.164	0.194	0.196
VML033	0.263	0.210	0.224	0.374	0.240	0.226	0.252	0.239
VML034	0.197	0.163	0.101	0.299	0.137	0.092	0.114	0.101
VML036	0.379	0.389	0.261	0.497	0.310	0.228	0.270	0.258
VML039	0.125	0.100	0.101	0.204	0.083	0.112	0.096	0.118
VML040	0.275	0.273	0.161	0.378	0.197	0.112	0.150	0.133
VML041	0.197	0.181	0.118	0.304	0.098	0.116	0.164	0.170
VML042	0.235	0.221	0.191	0.351	0.208	0.188	0.214	0.169
VML043	0.159	0.171	0.160	0.163	0.148	0.165	0.108	0.165
VML044	0.219	0.244	0.123	0.327	0.161	0.080	0.103	0.099
VML046	0.162	0.261	0.153	0.313	0.174	0.169	0.190	0.132
VML047	0.228	0.361	0.257	0.299	0.270	0.260	0.233	0.199
VML048	0.209	0.230	0.221	0.190	0.194	0.228	0.190	0.247

VML049	0.175	0.181	0.134	0.212	0.085	0.142	0.138	0.167
VML050	0.156	0.159	0.169	0.185	0.166	0.175	0.136	0.130
VML051	0.164	0.155	0.195	0.110	0.166	0.205	0.153	0.192
VML052	0.326	0.202	0.272	0.381	0.288	0.275	0.279	0.317
VML053	0.122	0.127	0.168	0.223	0.153	0.194	0.190	0.197
VML054	0.217	0.211	0.125	0.288	0.153	0.108	0.104	0.066
VML055	0.309	0.213	0.186	0.372	0.212	0.170	0.192	0.234
VML056	0.120	0.154	0.124	0.217	0.140	0.132	0.097	0.118
VML057		0.192	0.168	0.165	0.140	0.189	0.168	0.164
VML058	0.138		0.177	0.212	0.161	0.191	0.173	0.203
VML059	0.129	0.093		0.269	0.083	0.074	0.102	0.096
VML061	0.089	0.148	0.152		0.232	0.286	0.237	0.263
VML063	0.091	0.084	0.052	0.120		0.096	0.116	0.113
VML064	0.103	0.154	0.084	0.130	0.080		0.073	0.081
VML065	0.098	0.178	0.132	0.106	0.103	0.081		0.082
VML066	0.195	0.122	0.137	0.208	0.157	0.196	0.237	
VML067	0.109	0.059	0.067	0.142	0.060	0.117	0.155	0.110
VML068	0.169	0.090	0.109	0.180	0.120	0.174	0.200	0.061
VML069	0.131	0.224	0.204	0.102	0.167	0.149	0.088	0.294
VML070	0.071	0.093	0.082	0.099	0.040	0.095	0.091	0.170
VML073	0.143	0.093	0.054	0.179	0.077	0.122	0.160	0.129
VML074	0.095	0.179	0.169	0.080	0.135	0.121	0.110	0.249
VML075	0.101	0.189	0.153	0.109	0.133	0.119	0.089	0.209
VML076	0.185	0.107	0.175	0.156	0.163	0.221	0.222	0.150
VML080	0.183	0.140	0.089	0.196	0.130	0.134	0.194	0.098
VML081	0.076	0.184	0.160	0.135	0.136	0.127	0.132	0.200
VML083	0.123	0.249	0.225	0.136	0.189	0.165	0.116	0.298
VML084	0.118	0.187	0.131	0.164	0.127	0.086	0.133	0.201
VML085	0.177	0.061	0.133	0.174	0.134	0.192	0.220	0.136
VML086	0.138	0.174	0.193	0.104	0.153	0.175	0.142	0.275
VML087	0.111	0.115	0.099	0.117	0.084	0.123	0.118	0.139
VML088	0.118	0.192	0.153	0.153	0.125	0.098	0.109	0.260
VML089	0.143	0.083	0.120	0.111	0.103	0.148	0.161	0.172
VML090	0.169	0.084	0.106	0.210	0.110	0.173	0.206	0.149
VML091	0.116	0.155	0.113	0.110	0.111	0.098	0.113	0.155
VML092	0.173	0.093	0.093	0.156	0.111	0.149	0.177	0.128
VML093	0.155	0.069	0.072	0.167	0.096	0.138	0.185	0.089
VML094	0.072	0.100	0.066	0.106	0.042	0.076	0.096	0.148
VML095	0.070	0.078	0.106	0.083	0.067	0.122	0.122	0.163
VML096	0.153	0.157	0.086	0.177	0.114	0.105	0.147	0.137
VML097	0.142	0.094	0.084	0.154	0.096	0.138	0.166	0.077
VML099	0.094	0.149	0.086	0.112	0.073	0.034	0.054	0.197
VML100	0.177	0.067	0.130	0.204	0.125	0.197	0.224	0.125
VML102	0.195	0.082	0.159	0.210	0.157	0.222	0.253	0.117

VML104	0.155	0.065	0.146	0.139	0.126	0.192	0.197	0.157
VML106	0.219	0.124	0.155	0.244	0.172	0.228	0.262	0.081
VML107	0.160	0.093	0.081	0.187	0.101	0.152	0.182	0.103
VML110	0.199	0.119	0.140	0.218	0.158	0.208	0.239	0.058
VML111	0.132	0.189	0.121	0.180	0.128	0.094	0.135	0.184
VML112	0.129	0.137	0.070	0.166	0.084	0.096	0.124	0.151
VML113	0.151	0.112	0.180	0.124	0.151	0.203	0.198	0.206
VML114	0.186	0.113	0.134	0.207	0.153	0.191	0.239	0.040
VML116	0.098	0.136	0.078	0.137	0.058	0.058	0.076	0.203
VML117	0.224	0.123	0.163	0.235	0.182	0.234	0.268	0.085
VML118	0.317	0.292	0.278	0.368	0.297	0.320	0.358	0.208
VML119	0.175	0.271	0.233	0.194	0.219	0.198	0.168	0.270
VML120	0.091	0.150	0.119	0.116	0.093	0.075	0.101	0.222
VML121	0.059	0.121	0.118	0.048	0.081	0.097	0.091	0.188
VML122	0.111	0.103	0.096	0.083	0.076	0.112	0.102	0.171
VML123	0.171	0.072	0.101	0.195	0.113	0.177	0.205	0.094
VML124	0.142	0.243	0.185	0.156	0.167	0.115	0.081	0.282
VML125	0.239	0.320	0.251	0.298	0.255	0.213	0.231	0.310
VML126	0.135	0.060	0.101	0.117	0.083	0.148	0.150	0.148
VML127	0.143	0.118	0.134	0.167	0.134	0.167	0.204	0.085
VML128	0.058	0.159	0.127	0.107	0.088	0.082	0.057	0.223
VML129	0.131	0.133	0.057	0.165	0.084	0.077	0.138	0.137
VML130	0.086	0.184	0.155	0.079	0.122	0.095	0.068	0.239
VML131	0.252	0.198	0.159	0.268	0.200	0.212	0.259	0.117
VML132	0.092	0.063	0.112	0.103	0.080	0.142	0.148	0.147
VML133	0.183	0.270	0.202	0.230	0.205	0.154	0.175	0.258
VML134	0.327	0.214	0.281	0.316	0.287	0.353	0.367	0.205
VML136	0.120	0.156	0.142	0.185	0.125	0.139	0.177	0.191
VML137	0.137	0.240	0.183	0.167	0.162	0.117	0.077	0.288
VML138	0.219	0.135	0.129	0.205	0.158	0.193	0.220	0.120
VML139	0.082	0.103	0.101	0.144	0.069	0.112	0.135	0.183
VML140	0.408	0.315	0.336	0.429	0.363	0.403	0.452	0.224
VML141	0.219	0.131	0.182	0.179	0.176	0.229	0.229	0.198
VML143	0.121	0.115	0.084	0.170	0.097	0.117	0.169	0.105
VML144	0.424	0.319	0.376	0.425	0.390	0.446	0.477	0.256
VML145	0.104	0.180	0.133	0.136	0.105	0.078	0.063	0.254
VML146	0.073	0.086	0.084	0.088	0.055	0.101	0.105	0.154
VML147	0.202	0.131	0.203	0.156	0.184	0.239	0.236	0.193
VML148	0.096	0.136	0.123	0.111	0.090	0.096	0.112	0.221
VML150	0.086	0.090	0.058	0.109	0.037	0.084	0.102	0.146
VML151	0.161	0.085	0.059	0.185	0.094	0.132	0.186	0.093
VML152	0.207	0.280	0.259	0.174	0.228	0.202	0.159	0.361
VML153	0.155	0.241	0.209	0.135	0.178	0.146	0.096	0.314
VML154	0.244	0.129	0.208	0.254	0.207	0.277	0.298	0.151

VML155	0.153	0.111	0.180	0.135	0.152	0.213	0.204	0.162
VML156	0.116	0.203	0.151	0.139	0.139	0.108	0.089	0.224
VML157	0.114	0.149	0.094	0.134	0.099	0.084	0.120	0.141
VML158	0.215	0.314	0.286	0.201	0.254	0.217	0.177	0.385
VML159	0.137	0.137	0.074	0.192	0.086	0.099	0.145	0.183
VML160	0.139	0.086	0.086	0.156	0.098	0.134	0.180	0.073
VML161	0.121	0.101	0.152	0.111	0.114	0.176	0.157	0.194
VML162	0.140	0.083	0.060	0.146	0.073	0.108	0.146	0.161
VML164	0.112	0.061	0.110	0.107	0.087	0.140	0.159	0.145
VML165	0.132	0.162	0.080	0.169	0.091	0.072	0.103	0.186
VML168	0.082	0.082	0.123	0.094	0.080	0.139	0.133	0.181
VML170	0.048	0.149	0.143	0.107	0.116	0.123	0.131	0.178
VML171	0.139	0.213	0.141	0.190	0.143	0.099	0.123	0.225
VML173	0.275	0.188	0.215	0.301	0.236	0.283	0.324	0.104
VML174	0.056	0.140	0.123	0.067	0.083	0.084	0.070	0.211
VML175	0.147	0.076	0.143	0.121	0.118	0.179	0.178	0.175
VML176	0.207	0.158	0.131	0.252	0.160	0.183	0.242	0.111
VML178	0.183	0.102	0.165	0.220	0.157	0.222	0.251	0.126
VML179	0.085	0.161	0.132	0.078	0.100	0.098	0.045	0.215
VML180	0.079	0.141	0.097	0.102	0.065	0.054	0.055	0.205
VML183	0.097	0.128	0.114	0.056	0.094	0.101	0.100	0.170
VML184	0.104	0.132	0.122	0.051	0.098	0.102	0.099	0.193
VML188	0.202	0.100	0.157	0.212	0.160	0.220	0.252	0.085

	VML067	VML068	VML069	VML070	VML073	VML074	VML075	VML076
VML001	0.344	0.186	0.210	0.218	0.269	0.289	0.274	0.128
VML002	0.247	0.160	0.083	0.123	0.173	0.210	0.259	0.153
VML003	0.331	0.141	0.197	0.173	0.290	0.206	0.245	0.105
VML004	0.304	0.134	0.216	0.179	0.298	0.161	0.245	0.156
VML005	0.222	0.165	0.150	0.079	0.196	0.104	0.307	0.197
VML006	0.121	0.208	0.193	0.208	0.211	0.197	0.253	0.298
VML007	0.302	0.191	0.252	0.266	0.235	0.344	0.274	0.182
VML008	0.229	0.083	0.184	0.179	0.221	0.212	0.190	0.148
VML009	0.219	0.168	0.224	0.281	0.223	0.326	0.160	0.227
VML010	0.089	0.197	0.155	0.177	0.130	0.209	0.276	0.271
VML011	0.211	0.057	0.136	0.141	0.195	0.192	0.173	0.130
VML012	0.114	0.160	0.087	0.150	0.087	0.213	0.236	0.220
VML015	0.161	0.206	0.188	0.205	0.234	0.181	0.246	0.288
VML016	0.225	0.136	0.115	0.151	0.126	0.245	0.253	0.126
VML017	0.147	0.173	0.216	0.250	0.225	0.260	0.165	0.272
VML018	0.164	0.101	0.076	0.160	0.138	0.226	0.149	0.164
VML020	0.164	0.113	0.147	0.149	0.207	0.146	0.186	0.208
VML021	0.086	0.212	0.157	0.216	0.068	0.279	0.270	0.274
VML022	0.173	0.062	0.096	0.147	0.135	0.211	0.172	0.127

VML023	0.197	0.056	0.104	0.128	0.150	0.201	0.191	0.107
VML024	0.207	0.109	0.154	0.152	0.138	0.228	0.252	0.132
VML025	0.114	0.248	0.208	0.254	0.219	0.256	0.252	0.337
VML026	0.191	0.072	0.085	0.125	0.136	0.202	0.194	0.115
VML027	0.245	0.071	0.156	0.124	0.230	0.154	0.207	0.122
VML028	0.232	0.207	0.186	0.116	0.241	0.079	0.323	0.255
VML030	0.286	0.089	0.197	0.170	0.253	0.203	0.231	0.090
VML031	0.229	0.127	0.095	0.088	0.157	0.179	0.260	0.129
VML032	0.334	0.162	0.200	0.154	0.287	0.190	0.282	0.128
VML033	0.327	0.187	0.255	0.176	0.320	0.136	0.324	0.203
VML034	0.227	0.081	0.156	0.130	0.210	0.162	0.217	0.133
VML036	0.418	0.251	0.289	0.280	0.345	0.341	0.334	0.185
VML039	0.134	0.092	0.109	0.123	0.149	0.159	0.197	0.182
VML040	0.294	0.147	0.198	0.191	0.242	0.257	0.244	0.140
VML041	0.215	0.106	0.145	0.119	0.153	0.194	0.267	0.127
VML042	0.303	0.153	0.255	0.208	0.306	0.198	0.263	0.192
VML043	0.102	0.175	0.111	0.194	0.136	0.240	0.186	0.251
VML044	0.255	0.104	0.141	0.161	0.210	0.229	0.189	0.117
VML046	0.286	0.112	0.217	0.229	0.282	0.248	0.158	0.151
VML047	0.305	0.250	0.284	0.354	0.331	0.386	0.099	0.304
VML048	0.133	0.240	0.155	0.241	0.135	0.300	0.253	0.299
VML049	0.100	0.162	0.145	0.200	0.061	0.270	0.230	0.226
VML050	0.158	0.169	0.204	0.233	0.229	0.236	0.170	0.265
VML051	0.058	0.211	0.179	0.237	0.167	0.262	0.213	0.301
VML052	0.322	0.263	0.236	0.157	0.295	0.135	0.397	0.269
VML053	0.205	0.140	0.166	0.150	0.229	0.132	0.236	0.208
VML054	0.211	0.136	0.195	0.217	0.209	0.259	0.187	0.198
VML055	0.267	0.202	0.175	0.138	0.186	0.214	0.342	0.191
VML056	0.187	0.097	0.105	0.137	0.205	0.153	0.152	0.179
VML057	0.177	0.142	0.165	0.210	0.223	0.223	0.143	0.229
VML058	0.148	0.180	0.178	0.158	0.189	0.153	0.280	0.256
VML059	0.189	0.068	0.128	0.161	0.148	0.218	0.190	0.121
VML061	0.139	0.271	0.242	0.298	0.252	0.301	0.237	0.364
VML063	0.144	0.090	0.135	0.162	0.119	0.225	0.202	0.165
VML064	0.196	0.075	0.122	0.142	0.150	0.214	0.197	0.125
VML065	0.160	0.104	0.099	0.150	0.151	0.208	0.164	0.177
VML066	0.197	0.085	0.158	0.188	0.192	0.236	0.146	0.162
VML067		0.208	0.179	0.227	0.129	0.267	0.243	0.293
VML068	0.089		0.122	0.133	0.183	0.184	0.174	0.103
VML069	0.211	0.260		0.109	0.145	0.180	0.201	0.168
VML070	0.081	0.128	0.149		0.192	0.107	0.270	0.162
VML073	0.077	0.094	0.235	0.095		0.273	0.271	0.228
VML074	0.161	0.228	0.088	0.126	0.204		0.295	0.225
VML075	0.160	0.179	0.133	0.113	0.170	0.146		0.241

VML076	0.147	0.123	0.247	0.153	0.172	0.217	0.212	
VML080	0.108	0.107	0.261	0.155	0.108	0.220	0.185	0.196
VML081	0.141	0.186	0.168	0.125	0.172	0.140	0.092	0.230
VML083	0.221	0.270	0.091	0.168	0.248	0.120	0.116	0.280
VML084	0.135	0.194	0.185	0.139	0.161	0.145	0.133	0.259
VML085	0.107	0.121	0.258	0.141	0.131	0.206	0.231	0.095
VML086	0.187	0.240	0.112	0.135	0.216	0.100	0.191	0.192
VML087	0.099	0.094	0.178	0.072	0.105	0.169	0.093	0.142
VML088	0.160	0.237	0.134	0.134	0.187	0.098	0.167	0.267
VML089	0.107	0.146	0.184	0.110	0.147	0.138	0.188	0.114
VML090	0.093	0.120	0.268	0.126	0.074	0.227	0.224	0.174
VML091	0.122	0.137	0.166	0.110	0.141	0.149	0.075	0.185
VML092	0.111	0.100	0.230	0.122	0.104	0.199	0.186	0.115
VML093	0.065	0.078	0.243	0.117	0.077	0.195	0.189	0.133
VML094	0.066	0.115	0.160	0.042	0.088	0.130	0.099	0.165
VML095	0.077	0.129	0.156	0.047	0.118	0.117	0.132	0.127
VML096	0.119	0.120	0.224	0.127	0.099	0.204	0.127	0.212
VML097	0.075	0.041	0.229	0.105	0.084	0.199	0.144	0.135
VML099	0.119	0.166	0.131	0.076	0.121	0.118	0.096	0.206
VML100	0.087	0.102	0.274	0.140	0.116	0.228	0.231	0.144
VML102	0.108	0.115	0.293	0.168	0.150	0.237	0.250	0.131
VML104	0.114	0.125	0.222	0.119	0.148	0.183	0.204	0.063
VML106	0.131	0.077	0.324	0.182	0.123	0.285	0.242	0.162
VML107	0.087	0.060	0.253	0.110	0.045	0.225	0.175	0.153
VML110	0.119	0.053	0.300	0.164	0.116	0.264	0.211	0.149
VML111	0.138	0.172	0.206	0.137	0.138	0.184	0.115	0.255
VML112	0.104	0.116	0.206	0.092	0.069	0.192	0.119	0.200
VML113	0.148	0.180	0.200	0.136	0.189	0.157	0.212	0.101
VML114	0.098	0.082	0.293	0.168	0.125	0.239	0.217	0.154
VML116	0.111	0.167	0.152	0.066	0.101	0.135	0.128	0.211
VML117	0.143	0.089	0.323	0.188	0.137	0.281	0.249	0.130
VML118	0.262	0.228	0.429	0.307	0.250	0.396	0.314	0.336
VML119	0.240	0.246	0.199	0.197	0.239	0.226	0.096	0.286
VML120	0.123	0.201	0.130	0.101	0.156	0.072	0.151	0.220
VML121	0.105	0.160	0.114	0.068	0.146	0.072	0.109	0.158
VML122	0.109	0.129	0.147	0.062	0.119	0.135	0.119	0.128
VML123	0.085	0.055	0.269	0.123	0.071	0.233	0.200	0.136
VML124	0.209	0.254	0.113	0.158	0.215	0.143	0.105	0.290
VML125	0.270	0.299	0.290	0.257	0.255	0.290	0.210	0.386
VML126	0.091	0.104	0.190	0.080	0.116	0.163	0.163	0.100
VML127	0.083	0.101	0.248	0.144	0.142	0.194	0.174	0.164
VML128	0.130	0.190	0.108	0.075	0.149	0.096	0.098	0.218
VML129	0.086	0.123	0.212	0.108	0.084	0.177	0.139	0.208
VML130	0.157	0.214	0.071	0.113	0.189	0.057	0.102	0.221

VML131	0.178	0.129	0.334	0.217	0.150	0.305	0.232	0.236
VML132	0.070	0.115	0.181	0.069	0.121	0.138	0.150	0.117
VML133	0.219	0.249	0.231	0.206	0.216	0.226	0.145	0.326
VML134	0.256	0.197	0.406	0.286	0.258	0.373	0.349	0.167
VML136	0.109	0.184	0.222	0.139	0.155	0.164	0.185	0.242
VML137	0.209	0.255	0.121	0.150	0.204	0.153	0.116	0.291
VML138	0.149	0.095	0.280	0.167	0.125	0.257	0.211	0.140
VML139	0.082	0.155	0.186	0.074	0.101	0.140	0.164	0.183
VML140	0.312	0.264	0.513	0.382	0.317	0.463	0.423	0.336
VML141	0.178	0.164	0.252	0.172	0.186	0.231	0.243	0.082
VML143	0.065	0.102	0.234	0.112	0.080	0.187	0.153	0.185
VML144	0.341	0.282	0.520	0.397	0.354	0.474	0.445	0.290
VML145	0.156	0.221	0.112	0.103	0.162	0.105	0.138	0.247
VML146	0.078	0.117	0.156	0.033	0.095	0.128	0.112	0.133
VML147	0.173	0.170	0.242	0.175	0.211	0.208	0.237	0.061
VML148	0.118	0.197	0.133	0.098	0.158	0.071	0.163	0.205
VML150	0.065	0.109	0.166	0.038	0.081	0.138	0.110	0.157
VML151	0.063	0.083	0.252	0.122	0.062	0.206	0.191	0.166
VML152	0.272	0.331	0.094	0.218	0.297	0.132	0.221	0.309
VML153	0.226	0.281	0.049	0.167	0.243	0.102	0.161	0.274
VML154	0.168	0.141	0.340	0.210	0.184	0.294	0.291	0.131
VML155	0.136	0.132	0.221	0.136	0.179	0.192	0.188	0.070
VML156	0.173	0.195	0.149	0.123	0.162	0.163	0.059	0.234
VML157	0.105	0.126	0.186	0.108	0.118	0.160	0.093	0.195
VML158	0.295	0.358	0.113	0.244	0.323	0.147	0.227	0.349
VML159	0.107	0.155	0.222	0.106	0.066	0.192	0.174	0.225
VML160	0.051	0.076	0.234	0.118	0.100	0.183	0.168	0.148
VML161	0.122	0.154	0.174	0.094	0.162	0.149	0.167	0.128
VML162	0.090	0.133	0.201	0.092	0.084	0.162	0.181	0.155
VML164	0.070	0.124	0.187	0.091	0.132	0.131	0.169	0.119
VML165	0.125	0.155	0.190	0.103	0.097	0.181	0.121	0.230
VML168	0.093	0.145	0.159	0.062	0.132	0.119	0.153	0.128
VML170	0.114	0.163	0.163	0.101	0.153	0.121	0.101	0.191
VML171	0.170	0.203	0.197	0.144	0.148	0.192	0.119	0.272
VML173	0.186	0.134	0.385	0.248	0.188	0.339	0.294	0.215
VML174	0.119	0.181	0.096	0.072	0.152	0.060	0.110	0.186
VML175	0.118	0.141	0.198	0.111	0.152	0.162	0.195	0.077
VML176	0.122	0.128	0.313	0.186	0.119	0.265	0.232	0.236
VML178	0.109	0.121	0.297	0.165	0.143	0.243	0.244	0.156
VML179	0.143	0.176	0.096	0.078	0.154	0.112	0.066	0.188
VML180	0.112	0.173	0.116	0.068	0.133	0.093	0.109	0.204
VML183	0.112	0.144	0.131	0.087	0.147	0.105	0.099	0.149
VML184	0.124	0.167	0.116	0.093	0.159	0.085	0.122	0.157
VML188	0.109	0.092	0.297	0.174	0.153	0.246	0.238	0.139

	VML080	VML081	VML083	VML084	VML085	VML086	VML087	VML088
VML001	0.214	0.298	0.310	0.276	0.255	0.345	0.251	0.302
VML002	0.211	0.253	0.192	0.253	0.180	0.197	0.223	0.255
VML003	0.154	0.250	0.297	0.253	0.220	0.352	0.189	0.230
VML004	0.142	0.211	0.277	0.241	0.206	0.353	0.122	0.147
VML005	0.176	0.221	0.162	0.244	0.069	0.225	0.198	0.161
VML006	0.204	0.132	0.122	0.178	0.179	0.205	0.188	0.132
VML007	0.196	0.271	0.301	0.221	0.256	0.360	0.239	0.285
VML008	0.044	0.139	0.226	0.126	0.169	0.310	0.101	0.131
VML009	0.201	0.177	0.242	0.158	0.278	0.306	0.144	0.218
VML010	0.210	0.174	0.051	0.190	0.139	0.137	0.207	0.171
VML011	0.048	0.136	0.200	0.126	0.152	0.272	0.106	0.143
VML012	0.189	0.172	0.066	0.173	0.139	0.106	0.194	0.194
VML015	0.212	0.147	0.148	0.202	0.195	0.216	0.181	0.138
VML016	0.184	0.236	0.186	0.215	0.163	0.215	0.202	0.239
VML017	0.152	0.049	0.188	0.069	0.224	0.276	0.134	0.129
VML018	0.139	0.140	0.149	0.126	0.176	0.192	0.143	0.187
VML020	0.097	0.077	0.154	0.124	0.142	0.243	0.099	0.065
VML021	0.236	0.202	0.077	0.188	0.184	0.109	0.236	0.232
VML022	0.115	0.142	0.153	0.131	0.151	0.216	0.107	0.156
VML023	0.094	0.159	0.174	0.143	0.136	0.237	0.124	0.164
VML024	0.126	0.197	0.182	0.170	0.123	0.254	0.165	0.182
VML025	0.254	0.162	0.133	0.199	0.236	0.180	0.229	0.199
VML026	0.116	0.166	0.160	0.152	0.137	0.216	0.138	0.176
VML027	0.057	0.163	0.223	0.174	0.147	0.299	0.107	0.130
VML028	0.198	0.224	0.177	0.258	0.109	0.244	0.228	0.167
VML030	0.109	0.208	0.260	0.210	0.189	0.342	0.114	0.164
VML031	0.162	0.224	0.174	0.220	0.120	0.215	0.196	0.211
VML032	0.169	0.268	0.292	0.271	0.201	0.345	0.217	0.239
VML033	0.169	0.253	0.291	0.281	0.183	0.377	0.192	0.162
VML034	0.062	0.155	0.203	0.158	0.126	0.284	0.118	0.124
VML036	0.267	0.362	0.387	0.340	0.316	0.426	0.310	0.359
VML039	0.099	0.101	0.114	0.120	0.106	0.203	0.108	0.095
VML040	0.143	0.238	0.275	0.211	0.208	0.332	0.208	0.243
VML041	0.141	0.212	0.176	0.207	0.114	0.253	0.155	0.171
VML042	0.109	0.201	0.291	0.214	0.202	0.384	0.145	0.139
VML043	0.193	0.136	0.102	0.138	0.191	0.128	0.194	0.201
VML044	0.121	0.196	0.234	0.177	0.192	0.285	0.168	0.215
VML046	0.125	0.176	0.282	0.181	0.249	0.357	0.088	0.174
VML047	0.257	0.215	0.335	0.197	0.372	0.373	0.235	0.305
VML048	0.273	0.223	0.125	0.219	0.241	0.082	0.263	0.275
VML049	0.189	0.172	0.103	0.146	0.172	0.170	0.186	0.200
VML050	0.141	0.049	0.183	0.078	0.208	0.271	0.142	0.125

VML051	0.212	0.120	0.106	0.130	0.210	0.166	0.205	0.187
VML052	0.270	0.318	0.257	0.347	0.168	0.293	0.295	0.252
VML053	0.153	0.156	0.177	0.201	0.156	0.257	0.119	0.095
VML054	0.109	0.140	0.220	0.102	0.196	0.294	0.155	0.176
VML055	0.219	0.285	0.211	0.277	0.129	0.243	0.264	0.256
VML056	0.109	0.104	0.167	0.138	0.162	0.227	0.109	0.127
VML057	0.163	0.109	0.183	0.145	0.223	0.251	0.108	0.145
VML058	0.170	0.155	0.118	0.186	0.105	0.213	0.180	0.108
VML059	0.112	0.151	0.169	0.136	0.150	0.241	0.107	0.152
VML061	0.275	0.166	0.181	0.198	0.284	0.223	0.244	0.228
VML063	0.116	0.138	0.139	0.120	0.139	0.225	0.114	0.140
VML064	0.096	0.161	0.174	0.137	0.142	0.247	0.138	0.171
VML065	0.113	0.122	0.146	0.107	0.155	0.209	0.147	0.168
VML066	0.076	0.116	0.203	0.085	0.185	0.277	0.110	0.155
VML067	0.211	0.145	0.083	0.141	0.188	0.158	0.210	0.190
VML068	0.070	0.141	0.187	0.141	0.148	0.263	0.077	0.132
VML069	0.163	0.176	0.133	0.183	0.150	0.162	0.173	0.195
VML070	0.149	0.211	0.172	0.227	0.093	0.226	0.186	0.176
VML073	0.211	0.212	0.108	0.186	0.159	0.151	0.223	0.229
VML074	0.180	0.220	0.215	0.262	0.145	0.278	0.202	0.161
VML075	0.190	0.144	0.257	0.147	0.294	0.303	0.160	0.227
VML076	0.151	0.242	0.259	0.234	0.194	0.314	0.160	0.218
VML080		0.125	0.203	0.126	0.145	0.291	0.091	0.108
VML081	0.188		0.168	0.069	0.198	0.252	0.112	0.112
VML083	0.272	0.120		0.176	0.142	0.120	0.206	0.183
VML084	0.150	0.092	0.161		0.205	0.257	0.131	0.150
VML085	0.163	0.228	0.290	0.231		0.222	0.187	0.147
VML086	0.261	0.206	0.176	0.224	0.191		0.293	0.284
VML087	0.135	0.132	0.189	0.154	0.160	0.186		0.098
VML088	0.212	0.145	0.143	0.108	0.233	0.159	0.184	
VML089	0.164	0.200	0.232	0.196	0.092	0.126	0.136	0.177
VML090	0.158	0.208	0.283	0.202	0.113	0.223	0.153	0.209
VML091	0.119	0.113	0.169	0.114	0.192	0.201	0.079	0.168
VML092	0.113	0.214	0.269	0.205	0.097	0.197	0.115	0.221
VML093	0.083	0.188	0.265	0.174	0.084	0.212	0.118	0.203
VML094	0.124	0.103	0.167	0.106	0.150	0.164	0.063	0.131
VML095	0.166	0.130	0.176	0.155	0.118	0.121	0.091	0.148
VML096	0.079	0.147	0.223	0.122	0.196	0.248	0.098	0.189
VML097	0.086	0.156	0.238	0.160	0.132	0.222	0.066	0.208
VML099	0.143	0.124	0.151	0.103	0.190	0.161	0.100	0.108
VML100	0.163	0.212	0.289	0.214	0.099	0.225	0.152	0.225
VML102	0.174	0.227	0.308	0.234	0.085	0.236	0.176	0.250
VML104	0.189	0.210	0.257	0.230	0.066	0.154	0.136	0.223
VML106	0.147	0.233	0.329	0.241	0.138	0.291	0.162	0.286

VML107	0.108	0.183	0.264	0.183	0.128	0.234	0.096	0.219
VML110	0.126	0.208	0.303	0.218	0.138	0.277	0.134	0.270
VML111	0.133	0.100	0.180	0.064	0.235	0.248	0.131	0.151
VML112	0.111	0.138	0.208	0.128	0.183	0.222	0.079	0.172
VML113	0.229	0.213	0.239	0.241	0.107	0.115	0.169	0.215
VML114	0.104	0.196	0.296	0.195	0.120	0.266	0.152	0.249
VML116	0.156	0.138	0.171	0.119	0.180	0.160	0.114	0.103
VML117	0.152	0.249	0.337	0.258	0.111	0.280	0.170	0.298
VML118	0.243	0.283	0.395	0.288	0.321	0.436	0.275	0.372
VML119	0.252	0.139	0.150	0.191	0.312	0.270	0.171	0.239
VML120	0.179	0.134	0.152	0.107	0.185	0.131	0.155	0.055
VML121	0.170	0.115	0.140	0.133	0.156	0.110	0.102	0.120
VML122	0.152	0.161	0.188	0.170	0.135	0.131	0.073	0.166
VML123	0.127	0.199	0.282	0.203	0.104	0.234	0.116	0.232
VML124	0.228	0.137	0.098	0.127	0.286	0.201	0.173	0.124
VML125	0.262	0.190	0.237	0.173	0.367	0.355	0.254	0.238
VML126	0.153	0.184	0.226	0.191	0.098	0.148	0.091	0.187
VML127	0.121	0.139	0.239	0.148	0.149	0.238	0.130	0.207
VML128	0.192	0.096	0.107	0.108	0.206	0.140	0.119	0.083
VML129	0.073	0.134	0.214	0.092	0.174	0.225	0.108	0.152
VML130	0.203	0.116	0.093	0.122	0.220	0.129	0.141	0.097
VML131	0.098	0.244	0.336	0.221	0.218	0.337	0.177	0.295
VML132	0.164	0.140	0.196	0.164	0.108	0.142	0.098	0.165
VML133	0.207	0.132	0.183	0.114	0.314	0.299	0.198	0.193
VML134	0.280	0.359	0.430	0.381	0.190	0.344	0.265	0.404
VML136	0.177	0.118	0.200	0.100	0.200	0.220	0.172	0.125
VML137	0.237	0.143	0.109	0.142	0.283	0.197	0.175	0.126
VML138	0.118	0.245	0.310	0.239	0.138	0.257	0.135	0.273
VML139	0.171	0.136	0.194	0.142	0.141	0.156	0.132	0.127
VML140	0.282	0.403	0.510	0.392	0.312	0.484	0.353	0.457
VML141	0.216	0.276	0.307	0.287	0.109	0.184	0.174	0.275
VML143	0.093	0.124	0.226	0.116	0.151	0.230	0.112	0.178
VML144	0.343	0.435	0.528	0.451	0.299	0.470	0.367	0.498
VML145	0.205	0.145	0.135	0.126	0.221	0.140	0.156	0.065
VML146	0.144	0.126	0.177	0.146	0.125	0.138	0.067	0.152
VML147	0.230	0.252	0.285	0.277	0.114	0.172	0.178	0.268
VML148	0.187	0.148	0.163	0.128	0.170	0.114	0.155	0.070
VML150	0.122	0.121	0.180	0.121	0.140	0.162	0.062	0.138
VML151	0.067	0.183	0.267	0.158	0.114	0.230	0.124	0.196
VML152	0.317	0.246	0.162	0.240	0.306	0.140	0.258	0.161
VML153	0.267	0.193	0.119	0.190	0.273	0.128	0.203	0.125
VML154	0.223	0.279	0.357	0.298	0.113	0.274	0.209	0.314
VML155	0.211	0.193	0.240	0.236	0.127	0.175	0.130	0.241
VML156	0.186	0.115	0.137	0.139	0.241	0.205	0.116	0.170

VML157	0.097	0.107	0.182	0.095	0.188	0.215	0.082	0.161
VML158	0.340	0.237	0.140	0.234	0.346	0.185	0.282	0.163
VML159	0.139	0.164	0.229	0.137	0.175	0.216	0.139	0.151
VML160	0.077	0.154	0.242	0.142	0.118	0.219	0.109	0.190
VML161	0.212	0.172	0.198	0.202	0.139	0.124	0.123	0.181
VML162	0.124	0.191	0.240	0.170	0.101	0.164	0.127	0.164
VML164	0.153	0.159	0.212	0.164	0.094	0.142	0.119	0.162
VML165	0.127	0.141	0.194	0.112	0.207	0.218	0.107	0.146
VML168	0.187	0.148	0.184	0.174	0.118	0.108	0.112	0.152
VML170	0.176	0.048	0.136	0.110	0.187	0.177	0.117	0.146
VML171	0.171	0.124	0.176	0.110	0.253	0.245	0.149	0.160
VML173	0.181	0.275	0.381	0.280	0.194	0.356	0.221	0.340
VML174	0.183	0.115	0.124	0.126	0.176	0.105	0.117	0.095
VML175	0.192	0.207	0.241	0.224	0.082	0.127	0.134	0.206
VML176	0.103	0.202	0.306	0.171	0.187	0.305	0.179	0.236
VML178	0.186	0.209	0.298	0.225	0.119	0.249	0.175	0.248
VML179	0.186	0.122	0.121	0.145	0.201	0.137	0.089	0.138
VML180	0.162	0.118	0.138	0.101	0.186	0.138	0.110	0.078
VML183	0.147	0.131	0.160	0.140	0.159	0.141	0.085	0.151
VML184	0.164	0.150	0.162	0.151	0.159	0.115	0.112	0.139
VML188	0.152	0.220	0.306	0.223	0.118	0.258	0.162	0.257

	VML089	VML090	VML091	VML092	VML093	VML094	VML095	VML096
VML001	0.331	0.358	0.225	0.241	0.259	0.328	0.261	0.291
VML002	0.234	0.235	0.150	0.144	0.223	0.200	0.182	0.205
VML003	0.256	0.325	0.200	0.214	0.230	0.317	0.224	0.290
VML004	0.173	0.278	0.191	0.193	0.210	0.299	0.210	0.280
VML005	0.119	0.135	0.188	0.080	0.224	0.173	0.146	0.258
VML006	0.113	0.121	0.178	0.179	0.171	0.130	0.118	0.195
VML007	0.353	0.348	0.236	0.246	0.219	0.323	0.263	0.268
VML008	0.211	0.256	0.147	0.169	0.116	0.250	0.149	0.215
VML009	0.280	0.298	0.149	0.238	0.129	0.267	0.220	0.135
VML010	0.150	0.066	0.166	0.126	0.179	0.057	0.116	0.173
VML011	0.197	0.237	0.113	0.145	0.107	0.220	0.122	0.186
VML012	0.184	0.137	0.116	0.112	0.156	0.068	0.097	0.132
VML015	0.092	0.147	0.173	0.186	0.184	0.156	0.134	0.202
VML016	0.253	0.236	0.142	0.127	0.192	0.200	0.177	0.189
VML017	0.202	0.229	0.156	0.219	0.082	0.208	0.135	0.162
VML018	0.212	0.221	0.052	0.147	0.100	0.164	0.104	0.102
VML020	0.105	0.173	0.119	0.143	0.107	0.174	0.073	0.182
VML021	0.236	0.149	0.166	0.159	0.181	0.077	0.150	0.135
VML022	0.194	0.209	0.056	0.114	0.098	0.175	0.112	0.128
VML023	0.203	0.220	0.094	0.113	0.118	0.195	0.121	0.166
VML024	0.230	0.218	0.153	0.111	0.159	0.205	0.154	0.207

VML025	0.177	0.160	0.193	0.224	0.192	0.130	0.155	0.174
VML026	0.205	0.214	0.086	0.109	0.127	0.180	0.117	0.156
VML027	0.172	0.239	0.143	0.144	0.150	0.243	0.146	0.228
VML028	0.103	0.138	0.223	0.143	0.246	0.186	0.153	0.289
VML030	0.218	0.280	0.171	0.170	0.182	0.290	0.204	0.253
VML031	0.208	0.209	0.148	0.103	0.199	0.190	0.149	0.218
VML032	0.254	0.312	0.221	0.206	0.253	0.310	0.226	0.311
VML033	0.175	0.267	0.254	0.199	0.262	0.313	0.232	0.342
VML034	0.177	0.226	0.143	0.133	0.144	0.225	0.127	0.226
VML036	0.393	0.428	0.302	0.310	0.326	0.407	0.333	0.369
VML039	0.124	0.144	0.092	0.093	0.098	0.135	0.053	0.152
VML040	0.291	0.315	0.202	0.210	0.204	0.295	0.212	0.265
VML041	0.193	0.189	0.154	0.073	0.180	0.204	0.168	0.212
VML042	0.211	0.291	0.227	0.215	0.204	0.315	0.214	0.305
VML043	0.204	0.183	0.108	0.173	0.129	0.099	0.090	0.096
VML044	0.254	0.284	0.141	0.181	0.160	0.253	0.168	0.208
VML046	0.240	0.317	0.158	0.226	0.150	0.308	0.211	0.225
VML047	0.363	0.407	0.220	0.351	0.193	0.350	0.273	0.211
VML048	0.261	0.201	0.170	0.210	0.205	0.111	0.176	0.119
VML049	0.230	0.172	0.131	0.138	0.133	0.126	0.140	0.107
VML050	0.191	0.226	0.156	0.214	0.098	0.203	0.115	0.182
VML051	0.203	0.170	0.156	0.202	0.136	0.109	0.112	0.129
VML052	0.186	0.215	0.286	0.189	0.329	0.258	0.242	0.361
VML053	0.071	0.168	0.148	0.136	0.170	0.195	0.139	0.213
VML054	0.252	0.270	0.160	0.201	0.117	0.240	0.149	0.203
VML055	0.248	0.221	0.228	0.139	0.265	0.218	0.203	0.288
VML056	0.140	0.207	0.081	0.151	0.111	0.181	0.078	0.162
VML057	0.169	0.223	0.107	0.193	0.109	0.203	0.143	0.131
VML058	0.095	0.092	0.180	0.124	0.180	0.132	0.104	0.225
VML059	0.203	0.220	0.088	0.120	0.110	0.194	0.127	0.157
VML061	0.224	0.219	0.212	0.271	0.195	0.182	0.189	0.174
VML063	0.191	0.181	0.102	0.105	0.094	0.167	0.122	0.134
VML064	0.219	0.226	0.114	0.127	0.122	0.201	0.128	0.174
VML065	0.203	0.212	0.092	0.147	0.099	0.166	0.082	0.142
VML066	0.225	0.254	0.113	0.175	0.075	0.225	0.130	0.164
VML067	0.207	0.141	0.166	0.178	0.145	0.091	0.123	0.134
VML068	0.180	0.225	0.089	0.122	0.108	0.213	0.126	0.172
VML069	0.181	0.190	0.087	0.120	0.156	0.143	0.105	0.146
VML070	0.148	0.174	0.162	0.092	0.204	0.185	0.137	0.236
VML073	0.245	0.169	0.159	0.130	0.176	0.120	0.156	0.150
VML074	0.099	0.194	0.210	0.161	0.241	0.227	0.166	0.290
VML075	0.272	0.326	0.136	0.271	0.128	0.275	0.192	0.151
VML076	0.249	0.291	0.162	0.164	0.203	0.281	0.213	0.243
VML080	0.178	0.227	0.135	0.146	0.110	0.227	0.126	0.205

VML081	0.172	0.213	0.121	0.193	0.067	0.188	0.097	0.149
VML083	0.171	0.101	0.149	0.128	0.168	0.048	0.103	0.154
VML084	0.223	0.233	0.128	0.197	0.045	0.197	0.120	0.135
VML085	0.146	0.124	0.179	0.061	0.193	0.157	0.126	0.238
VML086	0.249	0.176	0.203	0.203	0.246	0.089	0.180	0.186
VML087	0.170	0.237	0.109	0.164	0.097	0.233	0.143	0.179
VML088	0.110	0.180	0.160	0.151	0.136	0.207	0.124	0.221
VML089		0.142	0.185	0.150	0.202	0.184	0.136	0.247
VML090	0.156		0.214	0.131	0.224	0.102	0.150	0.234
VML091	0.152	0.199		0.144	0.090	0.168	0.102	0.100
VML092	0.097	0.140	0.137		0.175	0.147	0.125	0.202
VML093	0.108	0.102	0.137	0.067		0.190	0.109	0.113
VML094	0.121	0.132	0.079	0.122	0.106		0.117	0.166
VML095	0.088	0.131	0.123	0.125	0.115	0.066		0.150
VML096	0.183	0.168	0.083	0.135	0.121	0.094	0.154	
VML097	0.136	0.129	0.097	0.092	0.073	0.083	0.112	0.085
VML099	0.142	0.174	0.085	0.142	0.142	0.065	0.109	0.103
VML100	0.140	0.073	0.199	0.143	0.103	0.142	0.128	0.186
VML102	0.142	0.113	0.213	0.152	0.112	0.167	0.143	0.209
VML104	0.079	0.136	0.180	0.114	0.117	0.138	0.088	0.203
VML106	0.198	0.115	0.205	0.150	0.116	0.172	0.179	0.170
VML107	0.155	0.087	0.143	0.100	0.079	0.103	0.127	0.103
VML110	0.184	0.129	0.171	0.134	0.106	0.150	0.162	0.143
VML111	0.212	0.194	0.101	0.194	0.168	0.102	0.163	0.078
VML112	0.172	0.137	0.100	0.132	0.119	0.071	0.129	0.053
VML113	0.090	0.178	0.199	0.155	0.161	0.160	0.096	0.238
VML114	0.164	0.134	0.166	0.133	0.080	0.147	0.155	0.149
VML116	0.145	0.142	0.125	0.148	0.140	0.070	0.106	0.120
VML117	0.182	0.135	0.207	0.127	0.106	0.183	0.178	0.185
VML118	0.360	0.263	0.291	0.313	0.269	0.281	0.316	0.239
VML119	0.281	0.293	0.154	0.266	0.268	0.183	0.216	0.184
VML120	0.130	0.179	0.140	0.176	0.160	0.099	0.107	0.166
VML121	0.097	0.175	0.101	0.141	0.138	0.072	0.058	0.153
VML122	0.087	0.158	0.100	0.090	0.116	0.076	0.073	0.134
VML123	0.148	0.071	0.167	0.107	0.078	0.120	0.126	0.139
VML124	0.228	0.265	0.140	0.240	0.242	0.148	0.185	0.172
VML125	0.348	0.303	0.230	0.327	0.302	0.232	0.288	0.201
VML126	0.069	0.129	0.135	0.086	0.101	0.094	0.072	0.154
VML127	0.156	0.157	0.131	0.158	0.111	0.116	0.127	0.141
VML128	0.158	0.182	0.121	0.186	0.175	0.078	0.099	0.150
VML129	0.159	0.141	0.094	0.132	0.101	0.073	0.132	0.052
VML130	0.153	0.227	0.115	0.192	0.192	0.110	0.120	0.172
VML131	0.241	0.197	0.179	0.165	0.149	0.190	0.232	0.118
VML132	0.090	0.125	0.133	0.128	0.111	0.078	0.033	0.160

	VML097	VML099	VML100	VML102	VML104	VML106	VML107	VML110
VML133	0.286	0.272	0.157	0.269	0.248	0.176	0.232	0.148
VML134	0.255	0.237	0.316	0.224	0.228	0.293	0.268	0.310
VML136	0.187	0.157	0.171	0.215	0.166	0.121	0.139	0.170
VML137	0.233	0.252	0.158	0.241	0.242	0.149	0.183	0.178
VML138	0.158	0.165	0.161	0.067	0.101	0.160	0.174	0.140
VML139	0.134	0.103	0.158	0.155	0.124	0.085	0.079	0.159
VML140	0.372	0.306	0.371	0.327	0.290	0.361	0.375	0.331
VML141	0.111	0.189	0.214	0.108	0.152	0.191	0.157	0.234
VML143	0.162	0.118	0.117	0.139	0.088	0.083	0.119	0.091
VML144	0.367	0.332	0.401	0.334	0.315	0.389	0.376	0.387
VML145	0.168	0.195	0.152	0.195	0.191	0.111	0.131	0.169
VML146	0.099	0.130	0.099	0.105	0.103	0.047	0.040	0.123
VML147	0.106	0.207	0.210	0.147	0.166	0.191	0.142	0.249
VML148	0.112	0.172	0.150	0.170	0.156	0.103	0.096	0.180
VML150	0.112	0.124	0.087	0.110	0.099	0.021	0.064	0.096
VML151	0.133	0.093	0.139	0.094	0.041	0.103	0.129	0.104
VML152	0.225	0.323	0.239	0.283	0.300	0.231	0.223	0.291
VML153	0.197	0.278	0.185	0.240	0.254	0.177	0.181	0.233
VML154	0.189	0.143	0.260	0.179	0.157	0.217	0.188	0.252
VML155	0.124	0.177	0.175	0.154	0.153	0.148	0.103	0.213
VML156	0.206	0.222	0.100	0.189	0.193	0.113	0.152	0.123
VML157	0.160	0.178	0.042	0.138	0.122	0.070	0.126	0.057
VML158	0.268	0.351	0.255	0.324	0.330	0.251	0.250	0.307
VML159	0.175	0.104	0.158	0.153	0.125	0.100	0.138	0.116
VML160	0.122	0.127	0.113	0.108	0.058	0.092	0.111	0.110
VML161	0.108	0.158	0.167	0.159	0.159	0.117	0.068	0.200
VML162	0.087	0.111	0.143	0.074	0.075	0.098	0.101	0.137
VML164	0.061	0.134	0.137	0.118	0.100	0.093	0.058	0.166
VML165	0.182	0.160	0.107	0.153	0.143	0.084	0.144	0.069
VML168	0.088	0.134	0.148	0.138	0.130	0.089	0.028	0.178
VML170	0.164	0.181	0.109	0.184	0.159	0.085	0.092	0.148
VML171	0.231	0.207	0.130	0.207	0.192	0.124	0.178	0.108
VML173	0.256	0.180	0.251	0.211	0.171	0.231	0.241	0.215
VML174	0.117	0.182	0.114	0.159	0.155	0.079	0.076	0.159
VML175	0.058	0.148	0.173	0.111	0.123	0.133	0.082	0.203
VML176	0.216	0.134	0.187	0.180	0.124	0.159	0.195	0.132
VML178	0.173	0.102	0.217	0.178	0.129	0.164	0.145	0.207
VML179	0.146	0.200	0.094	0.158	0.170	0.085	0.101	0.141
VML180	0.129	0.171	0.104	0.155	0.149	0.065	0.094	0.131
VML183	0.099	0.190	0.067	0.120	0.129	0.078	0.084	0.132
VML184	0.084	0.195	0.097	0.126	0.140	0.094	0.088	0.155
VML188	0.154	0.137	0.194	0.152	0.112	0.163	0.155	0.189

VML001	0.228	0.285	0.275	0.221	0.334	0.144	0.176	0.263
VML002	0.194	0.228	0.204	0.150	0.194	0.190	0.122	0.167
VML003	0.189	0.243	0.227	0.168	0.307	0.138	0.186	0.247
VML004	0.171	0.185	0.179	0.151	0.266	0.198	0.225	0.241
VML005	0.153	0.157	0.085	0.117	0.178	0.256	0.192	0.211
VML006	0.163	0.156	0.160	0.160	0.108	0.291	0.222	0.166
VML007	0.202	0.246	0.267	0.240	0.341	0.174	0.189	0.272
VML008	0.080	0.138	0.160	0.119	0.248	0.137	0.149	0.192
VML009	0.184	0.191	0.254	0.215	0.233	0.184	0.186	0.174
VML010	0.157	0.144	0.137	0.152	0.074	0.283	0.186	0.154
VML011	0.075	0.142	0.153	0.082	0.217	0.118	0.109	0.156
VML012	0.138	0.164	0.156	0.125	0.088	0.220	0.110	0.102
VML015	0.178	0.174	0.172	0.159	0.107	0.285	0.226	0.164
VML016	0.157	0.197	0.187	0.151	0.210	0.159	0.087	0.170
VML017	0.129	0.150	0.201	0.170	0.194	0.226	0.196	0.160
VML018	0.111	0.169	0.185	0.105	0.156	0.124	0.034	0.070
VML020	0.083	0.112	0.122	0.076	0.155	0.200	0.161	0.141
VML021	0.179	0.185	0.195	0.189	0.115	0.269	0.160	0.145
VML022	0.080	0.132	0.150	0.091	0.163	0.118	0.058	0.104
VML023	0.079	0.141	0.146	0.085	0.195	0.113	0.066	0.138
VML024	0.098	0.138	0.137	0.133	0.225	0.166	0.116	0.191
VML025	0.212	0.208	0.223	0.205	0.095	0.315	0.237	0.159
VML026	0.092	0.150	0.151	0.090	0.182	0.120	0.048	0.126
VML027	0.101	0.146	0.140	0.083	0.232	0.148	0.152	0.190
VML028	0.183	0.185	0.118	0.145	0.194	0.298	0.236	0.237
VML030	0.140	0.158	0.168	0.146	0.272	0.150	0.183	0.229
VML031	0.144	0.185	0.151	0.116	0.202	0.177	0.110	0.177
VML032	0.198	0.247	0.217	0.172	0.310	0.179	0.200	0.264
VML033	0.196	0.197	0.160	0.182	0.301	0.268	0.273	0.299
VML034	0.076	0.137	0.126	0.089	0.227	0.151	0.141	0.187
VML036	0.293	0.345	0.335	0.289	0.413	0.207	0.257	0.342
VML039	0.055	0.090	0.096	0.053	0.127	0.186	0.119	0.116
VML040	0.170	0.229	0.226	0.177	0.308	0.143	0.160	0.240
VML041	0.124	0.112	0.106	0.125	0.202	0.200	0.150	0.197
VML042	0.156	0.176	0.179	0.172	0.307	0.217	0.244	0.274
VML043	0.150	0.191	0.202	0.142	0.101	0.213	0.117	0.064
VML044	0.139	0.205	0.207	0.133	0.255	0.095	0.104	0.179
VML046	0.158	0.191	0.225	0.169	0.276	0.125	0.190	0.209
VML047	0.265	0.317	0.364	0.281	0.324	0.194	0.239	0.223
VML048	0.226	0.242	0.253	0.214	0.113	0.277	0.173	0.128
VML049	0.137	0.140	0.172	0.162	0.139	0.222	0.127	0.131
VML050	0.121	0.159	0.193	0.157	0.198	0.219	0.189	0.161
VML051	0.164	0.183	0.207	0.178	0.114	0.265	0.182	0.122
VML052	0.257	0.266	0.188	0.214	0.265	0.337	0.285	0.305

VML053	0.139	0.128	0.120	0.111	0.153	0.234	0.199	0.175
VML054	0.107	0.176	0.200	0.155	0.253	0.152	0.144	0.186
VML055	0.196	0.228	0.175	0.182	0.252	0.251	0.184	0.249
VML056	0.102	0.158	0.159	0.062	0.155	0.153	0.120	0.104
VML057	0.150	0.156	0.196	0.142	0.150	0.202	0.175	0.120
VML058	0.128	0.123	0.091	0.130	0.141	0.274	0.204	0.188
VML059	0.074	0.129	0.146	0.108	0.189	0.120	0.083	0.136
VML061	0.238	0.239	0.269	0.239	0.147	0.324	0.259	0.174
VML063	0.076	0.083	0.126	0.111	0.162	0.174	0.112	0.136
VML064	0.084	0.137	0.150	0.107	0.210	0.129	0.085	0.155
VML065	0.091	0.157	0.167	0.096	0.174	0.144	0.074	0.108
VML066	0.082	0.150	0.182	0.119	0.223	0.115	0.110	0.149
VML067	0.159	0.160	0.186	0.179	0.114	0.274	0.181	0.142
VML068	0.074	0.121	0.137	0.077	0.197	0.114	0.103	0.144
VML069	0.138	0.179	0.167	0.092	0.132	0.170	0.081	0.096
VML070	0.144	0.165	0.115	0.088	0.187	0.216	0.157	0.189
VML073	0.158	0.169	0.177	0.174	0.156	0.238	0.132	0.158
VML074	0.185	0.196	0.142	0.132	0.213	0.268	0.232	0.233
VML075	0.194	0.244	0.284	0.197	0.241	0.148	0.173	0.143
VML076	0.162	0.199	0.195	0.155	0.269	0.119	0.146	0.217
VML080	0.067	0.115	0.133	0.088	0.223	0.154	0.146	0.178
VML081	0.105	0.141	0.180	0.129	0.169	0.198	0.165	0.128
VML083	0.149	0.150	0.148	0.149	0.082	0.262	0.160	0.135
VML084	0.102	0.145	0.194	0.149	0.196	0.182	0.147	0.137
VML085	0.117	0.120	0.056	0.107	0.181	0.244	0.172	0.203
VML086	0.242	0.259	0.245	0.219	0.117	0.308	0.197	0.165
VML087	0.092	0.118	0.157	0.114	0.202	0.148	0.156	0.158
VML088	0.096	0.091	0.107	0.116	0.190	0.228	0.201	0.191
VML089	0.161	0.155	0.121	0.127	0.155	0.275	0.226	0.199
VML090	0.182	0.156	0.124	0.172	0.128	0.321	0.229	0.210
VML091	0.106	0.154	0.175	0.097	0.139	0.128	0.074	0.061
VML092	0.110	0.110	0.070	0.097	0.155	0.217	0.142	0.173
VML093	0.086	0.131	0.179	0.123	0.175	0.155	0.123	0.111
VML094	0.175	0.183	0.172	0.164	0.077	0.282	0.174	0.143
VML095	0.087	0.132	0.130	0.075	0.119	0.198	0.122	0.104
VML096	0.168	0.196	0.234	0.176	0.141	0.193	0.125	0.074
VML097		0.084	0.106	0.077	0.175	0.162	0.118	0.143
VML099	0.130		0.080	0.123	0.180	0.227	0.179	0.187
VML100	0.121	0.195		0.109	0.179	0.246	0.187	0.205
VML102	0.137	0.222	0.058		0.153	0.167	0.114	0.128
VML104	0.132	0.181	0.109	0.104		0.264	0.173	0.112
VML106	0.110	0.225	0.103	0.108	0.160		0.117	0.171
VML107	0.062	0.145	0.109	0.138	0.140	0.089		0.096
VML110	0.081	0.203	0.116	0.118	0.153	0.042	0.078	

VML111	0.137	0.101	0.214	0.239	0.235	0.217	0.154	0.193
VML112	0.087	0.087	0.165	0.198	0.183	0.164	0.082	0.143
VML113	0.178	0.193	0.158	0.148	0.063	0.216	0.191	0.207
VML114	0.093	0.196	0.114	0.100	0.151	0.087	0.110	0.078
VML116	0.138	0.051	0.175	0.210	0.175	0.215	0.134	0.200
VML117	0.120	0.231	0.124	0.111	0.143	0.062	0.108	0.069
VML118	0.237	0.323	0.264	0.275	0.337	0.197	0.231	0.198
VML119	0.215	0.179	0.307	0.325	0.286	0.298	0.238	0.266
VML120	0.172	0.085	0.193	0.211	0.178	0.250	0.186	0.234
VML121	0.132	0.085	0.173	0.186	0.130	0.221	0.161	0.199
VML122	0.105	0.089	0.162	0.180	0.110	0.193	0.124	0.170
VML123	0.077	0.172	0.072	0.099	0.119	0.064	0.044	0.066
VML124	0.216	0.104	0.285	0.311	0.266	0.313	0.236	0.287
VML125	0.269	0.219	0.335	0.365	0.366	0.329	0.272	0.309
VML126	0.097	0.132	0.114	0.130	0.067	0.159	0.111	0.143
VML127	0.093	0.170	0.127	0.120	0.152	0.136	0.133	0.114
VML128	0.159	0.069	0.197	0.226	0.184	0.244	0.172	0.225
VML129	0.090	0.088	0.161	0.185	0.186	0.169	0.103	0.148
VML130	0.180	0.085	0.231	0.249	0.196	0.277	0.209	0.253
VML131	0.126	0.214	0.205	0.216	0.243	0.139	0.129	0.121
VML132	0.105	0.132	0.107	0.117	0.075	0.161	0.123	0.145
VML133	0.214	0.160	0.295	0.319	0.310	0.292	0.232	0.266
VML134	0.233	0.342	0.203	0.181	0.195	0.170	0.225	0.182
VML136	0.166	0.153	0.159	0.180	0.201	0.211	0.176	0.202
VML137	0.220	0.105	0.280	0.311	0.265	0.310	0.229	0.287
VML138	0.100	0.184	0.167	0.175	0.156	0.134	0.102	0.116
VML139	0.140	0.115	0.130	0.158	0.140	0.187	0.131	0.181
VML140	0.292	0.409	0.278	0.261	0.346	0.214	0.290	0.231
VML141	0.172	0.215	0.174	0.172	0.093	0.202	0.176	0.194
VML143	0.081	0.126	0.132	0.148	0.166	0.133	0.090	0.116
VML144	0.318	0.444	0.290	0.258	0.318	0.238	0.320	0.254
VML145	0.190	0.073	0.222	0.252	0.208	0.272	0.194	0.256
VML146	0.092	0.084	0.140	0.159	0.107	0.172	0.106	0.152
VML147	0.177	0.226	0.170	0.151	0.073	0.210	0.199	0.198
VML148	0.172	0.101	0.179	0.197	0.159	0.246	0.186	0.232
VML150	0.079	0.071	0.135	0.160	0.128	0.165	0.093	0.143
VML151	0.075	0.139	0.104	0.123	0.144	0.112	0.070	0.103
VML152	0.300	0.193	0.331	0.348	0.278	0.392	0.321	0.371
VML153	0.249	0.134	0.291	0.313	0.246	0.344	0.268	0.322
VML154	0.175	0.272	0.103	0.080	0.126	0.111	0.161	0.132
VML155	0.138	0.197	0.137	0.132	0.073	0.174	0.163	0.160
VML156	0.159	0.089	0.246	0.270	0.225	0.251	0.175	0.224
VML157	0.087	0.081	0.184	0.202	0.186	0.188	0.125	0.159
VML158	0.325	0.212	0.360	0.378	0.317	0.419	0.348	0.397

VML159	0.136	0.108	0.157	0.194	0.190	0.180	0.108	0.175
VML160	0.061	0.139	0.109	0.113	0.132	0.123	0.097	0.103
VML161	0.147	0.159	0.137	0.150	0.085	0.198	0.160	0.184
VML162	0.117	0.109	0.136	0.155	0.121	0.173	0.109	0.163
VML164	0.113	0.136	0.110	0.111	0.076	0.171	0.137	0.156
VML165	0.122	0.067	0.192	0.227	0.207	0.204	0.121	0.184
VML168	0.133	0.127	0.128	0.144	0.082	0.191	0.143	0.179
VML170	0.137	0.119	0.184	0.193	0.169	0.210	0.163	0.186
VML171	0.170	0.101	0.244	0.273	0.254	0.246	0.172	0.225
VML173	0.164	0.285	0.158	0.147	0.221	0.076	0.157	0.093
VML174	0.152	0.073	0.188	0.207	0.153	0.240	0.173	0.220
VML175	0.140	0.167	0.127	0.129	0.033	0.185	0.152	0.175
VML176	0.128	0.196	0.142	0.160	0.218	0.122	0.120	0.122
VML178	0.142	0.223	0.062	0.060	0.130	0.104	0.134	0.119
VML179	0.144	0.068	0.210	0.234	0.172	0.241	0.166	0.216
VML180	0.141	0.043	0.184	0.211	0.170	0.231	0.158	0.212
VML183	0.113	0.085	0.183	0.193	0.137	0.214	0.154	0.186
VML184	0.138	0.089	0.191	0.200	0.137	0.233	0.171	0.209
VML188	0.116	0.219	0.077	0.057	0.127	0.098	0.133	0.098

	VML111	VML112	VML113	VML114	VML116	VML117	VML118	VML119
VML001	0.324	0.252	0.220	0.299	0.343	0.280	0.218	0.186
VML002	0.248	0.224	0.184	0.363	0.246	0.153	0.283	0.240
VML003	0.329	0.225	0.125	0.225	0.333	0.250	0.173	0.176
VML004	0.325	0.191	0.097	0.203	0.312	0.237	0.171	0.230
VML005	0.302	0.207	0.133	0.335	0.275	0.068	0.276	0.323
VML006	0.219	0.201	0.246	0.340	0.168	0.175	0.314	0.345
VML007	0.291	0.192	0.278	0.262	0.323	0.308	0.171	0.221
VML008	0.247	0.108	0.163	0.160	0.251	0.226	0.108	0.193
VML009	0.161	0.128	0.287	0.268	0.184	0.303	0.203	0.203
VML010	0.203	0.188	0.247	0.373	0.164	0.120	0.317	0.345
VML011	0.222	0.115	0.142	0.198	0.225	0.193	0.143	0.176
VML012	0.167	0.176	0.226	0.358	0.146	0.116	0.290	0.286
VML015	0.231	0.214	0.230	0.342	0.179	0.181	0.316	0.333
VML016	0.230	0.176	0.204	0.333	0.241	0.173	0.235	0.229
VML017	0.173	0.139	0.263	0.240	0.157	0.255	0.228	0.267
VML018	0.140	0.139	0.202	0.285	0.147	0.182	0.220	0.184
VML020	0.216	0.137	0.158	0.237	0.188	0.167	0.214	0.251
VML021	0.156	0.193	0.294	0.396	0.140	0.171	0.326	0.332
VML022	0.174	0.100	0.175	0.263	0.177	0.173	0.182	0.189
VML023	0.207	0.113	0.155	0.247	0.213	0.170	0.167	0.185
VML024	0.245	0.123	0.189	0.271	0.251	0.177	0.178	0.242
VML025	0.185	0.236	0.301	0.388	0.125	0.215	0.360	0.360
VML026	0.198	0.127	0.165	0.271	0.203	0.160	0.190	0.193

VML027	0.269	0.141	0.087	0.191	0.264	0.188	0.145	0.197
VML028	0.326	0.246	0.162	0.347	0.290	0.101	0.313	0.359
VML030	0.298	0.139	0.127	0.186	0.300	0.236	0.103	0.190
VML031	0.260	0.190	0.153	0.321	0.256	0.127	0.240	0.245
VML032	0.350	0.243	0.123	0.259	0.350	0.231	0.207	0.224
VML033	0.386	0.232	0.110	0.239	0.367	0.228	0.215	0.313
VML034	0.264	0.136	0.122	0.206	0.257	0.180	0.151	0.218
VML036	0.400	0.312	0.265	0.312	0.422	0.349	0.244	0.229
VML039	0.192	0.111	0.160	0.264	0.170	0.130	0.213	0.248
VML040	0.295	0.197	0.194	0.232	0.311	0.252	0.168	0.191
VML041	0.261	0.124	0.158	0.290	0.257	0.151	0.198	0.258
VML042	0.341	0.180	0.149	0.133	0.333	0.263	0.131	0.256
VML043	0.114	0.187	0.254	0.344	0.088	0.175	0.296	0.267
VML044	0.241	0.171	0.171	0.236	0.255	0.222	0.171	0.150
VML046	0.261	0.146	0.181	0.145	0.269	0.287	0.103	0.137
VML047	0.200	0.256	0.348	0.266	0.233	0.390	0.265	0.169
VML048	0.131	0.241	0.316	0.427	0.113	0.205	0.364	0.324
VML049	0.141	0.129	0.264	0.340	0.142	0.183	0.263	0.280
VML050	0.194	0.157	0.247	0.238	0.174	0.241	0.231	0.266
VML051	0.132	0.192	0.291	0.349	0.088	0.207	0.315	0.317
VML052	0.403	0.317	0.182	0.409	0.372	0.147	0.360	0.401
VML053	0.258	0.166	0.145	0.280	0.225	0.163	0.241	0.281
VML054	0.221	0.141	0.228	0.206	0.231	0.246	0.165	0.213
VML055	0.325	0.246	0.201	0.373	0.318	0.141	0.292	0.325
VML056	0.198	0.157	0.145	0.248	0.179	0.169	0.216	0.204
VML057	0.167	0.147	0.217	0.267	0.146	0.228	0.236	0.227
VML058	0.258	0.183	0.201	0.322	0.219	0.123	0.282	0.341
VML059	0.199	0.096	0.180	0.251	0.203	0.187	0.163	0.198
VML061	0.170	0.252	0.337	0.389	0.116	0.270	0.370	0.359
VML063	0.177	0.057	0.198	0.266	0.178	0.175	0.186	0.233
VML064	0.211	0.111	0.177	0.245	0.220	0.184	0.166	0.196
VML065	0.170	0.140	0.195	0.265	0.170	0.176	0.212	0.205
VML066	0.191	0.103	0.195	0.194	0.202	0.227	0.143	0.170
VML067	0.146	0.176	0.290	0.359	0.115	0.190	0.311	0.331
VML068	0.216	0.095	0.133	0.215	0.217	0.184	0.141	0.174
VML069	0.188	0.179	0.174	0.324	0.176	0.129	0.260	0.225
VML070	0.280	0.195	0.105	0.310	0.263	0.089	0.252	0.273
VML073	0.180	0.167	0.268	0.372	0.182	0.163	0.287	0.305
VML074	0.331	0.244	0.108	0.310	0.299	0.141	0.285	0.317
VML075	0.160	0.198	0.267	0.244	0.175	0.306	0.230	0.144
VML076	0.289	0.171	0.143	0.252	0.298	0.225	0.153	0.174
VML080	0.241	0.103	0.139	0.176	0.238	0.197	0.133	0.205
VML081	0.168	0.135	0.224	0.235	0.148	0.223	0.219	0.241
VML083	0.183	0.183	0.250	0.369	0.150	0.127	0.309	0.325

VML084	0.148	0.112	0.249	0.233	0.151	0.240	0.201	0.229
VML085	0.278	0.169	0.156	0.311	0.258	0.082	0.249	0.314
VML086	0.201	0.276	0.311	0.457	0.172	0.170	0.395	0.366
VML087	0.218	0.094	0.165	0.190	0.211	0.224	0.141	0.193
VML088	0.256	0.131	0.168	0.224	0.230	0.190	0.199	0.281
VML089	0.285	0.210	0.159	0.312	0.244	0.143	0.286	0.326
VML090	0.266	0.218	0.244	0.390	0.227	0.105	0.338	0.385
VML091	0.145	0.129	0.189	0.274	0.140	0.185	0.212	0.183
VML092	0.248	0.145	0.155	0.314	0.232	0.087	0.237	0.286
VML093	0.140	0.091	0.221	0.229	0.141	0.222	0.186	0.203
VML094	0.189	0.213	0.265	0.394	0.152	0.125	0.338	0.345
VML095	0.181	0.150	0.185	0.286	0.154	0.134	0.246	0.259
VML096	0.053	0.162	0.280	0.331	0.071	0.235	0.273	0.231
VML097	0.205	0.081	0.165	0.228	0.197	0.166	0.168	0.230
VML099	0.235	0.081	0.191	0.255	0.220	0.173	0.195	0.284
VML100	0.277	0.144	0.153	0.289	0.254	0.115	0.229	0.311
VML102	0.218	0.136	0.116	0.255	0.201	0.125	0.206	0.226
VML104	0.173	0.205	0.247	0.379	0.126	0.146	0.327	0.318
VML106	0.224	0.173	0.203	0.228	0.245	0.269	0.157	0.088
VML107	0.162	0.147	0.200	0.293	0.173	0.181	0.217	0.185
VML110	0.109	0.170	0.230	0.318	0.093	0.191	0.266	0.219
VML111		0.200	0.325	0.355	0.062	0.274	0.307	0.256
VML112	0.087		0.209	0.222	0.205	0.218	0.145	0.225
VML113	0.257	0.217		0.252	0.310	0.176	0.211	0.247
VML114	0.186	0.158	0.195		0.363	0.368	0.126	0.231
VML116	0.116	0.085	0.189	0.198		0.246	0.322	0.283
VML117	0.239	0.183	0.196	0.086	0.226		0.309	0.335
VML118	0.250	0.248	0.383	0.218	0.319	0.242		0.186
VML119	0.160	0.181	0.293	0.282	0.205	0.309	0.323	
VML120	0.145	0.151	0.171	0.209	0.086	0.254	0.357	0.234
VML121	0.151	0.137	0.125	0.182	0.102	0.218	0.341	0.199
VML122	0.165	0.115	0.128	0.175	0.101	0.185	0.335	0.207
VML123	0.184	0.120	0.174	0.097	0.157	0.086	0.235	0.268
VML124	0.132	0.163	0.264	0.284	0.130	0.324	0.374	0.147
VML125	0.142	0.203	0.382	0.312	0.223	0.359	0.287	0.190
VML126	0.191	0.135	0.107	0.152	0.130	0.156	0.321	0.249
VML127	0.152	0.149	0.182	0.079	0.178	0.150	0.231	0.240
VML128	0.121	0.122	0.183	0.218	0.070	0.256	0.334	0.174
VML129	0.073	0.062	0.217	0.136	0.095	0.185	0.250	0.208
VML130	0.149	0.161	0.184	0.236	0.115	0.278	0.373	0.183
VML131	0.181	0.149	0.294	0.140	0.222	0.154	0.192	0.275
VML132	0.173	0.138	0.095	0.140	0.126	0.164	0.304	0.232
VML133	0.085	0.159	0.324	0.261	0.180	0.313	0.284	0.142
VML134	0.365	0.301	0.242	0.213	0.335	0.144	0.335	0.406

VML136	0.133	0.157	0.212	0.174	0.140	0.236	0.274	0.249
VML137	0.139	0.157	0.263	0.288	0.116	0.321	0.373	0.152
VML138	0.213	0.146	0.207	0.139	0.191	0.112	0.287	0.277
VML139	0.152	0.124	0.149	0.165	0.085	0.195	0.303	0.238
VML140	0.373	0.348	0.399	0.231	0.406	0.232	0.222	0.468
VML141	0.284	0.219	0.121	0.201	0.215	0.168	0.388	0.323
VML143	0.102	0.088	0.198	0.094	0.124	0.149	0.220	0.216
VML144	0.436	0.393	0.363	0.263	0.441	0.227	0.322	0.493
VML145	0.144	0.141	0.204	0.247	0.063	0.281	0.368	0.210
VML146	0.142	0.097	0.124	0.151	0.086	0.170	0.304	0.195
VML147	0.285	0.238	0.080	0.192	0.231	0.180	0.385	0.316
VML148	0.166	0.161	0.150	0.207	0.096	0.249	0.365	0.250
VML150	0.114	0.072	0.154	0.146	0.070	0.175	0.285	0.194
VML151	0.148	0.103	0.188	0.085	0.132	0.121	0.246	0.265
VML152	0.272	0.276	0.247	0.356	0.208	0.388	0.505	0.283
VML153	0.213	0.216	0.225	0.310	0.152	0.342	0.447	0.225
VML154	0.292	0.236	0.174	0.143	0.257	0.101	0.289	0.357
VML155	0.238	0.196	0.094	0.164	0.200	0.165	0.326	0.260
VML156	0.110	0.110	0.234	0.229	0.116	0.256	0.318	0.102
VML157	0.072	0.076	0.210	0.148	0.112	0.197	0.263	0.165
VML158	0.271	0.294	0.286	0.379	0.229	0.420	0.508	0.274
VML159	0.121	0.081	0.217	0.173	0.073	0.198	0.273	0.237
VML160	0.141	0.118	0.171	0.067	0.146	0.129	0.246	0.245
VML161	0.212	0.174	0.090	0.192	0.151	0.202	0.344	0.246
VML162	0.171	0.120	0.147	0.151	0.097	0.164	0.324	0.265
VML164	0.183	0.154	0.093	0.135	0.136	0.168	0.319	0.257
VML165	0.077	0.044	0.234	0.190	0.067	0.221	0.278	0.183
VML168	0.186	0.149	0.082	0.171	0.117	0.189	0.334	0.236
VML170	0.122	0.136	0.170	0.170	0.130	0.217	0.290	0.164
VML171	0.070	0.097	0.269	0.224	0.109	0.261	0.285	0.148
VML173	0.257	0.221	0.274	0.112	0.278	0.106	0.165	0.341
VML174	0.147	0.139	0.145	0.204	0.086	0.240	0.353	0.198
VML175	0.233	0.182	0.054	0.169	0.163	0.167	0.357	0.281
VML176	0.154	0.141	0.262	0.103	0.187	0.157	0.174	0.287
VML178	0.226	0.189	0.169	0.106	0.205	0.123	0.244	0.309
VML179	0.141	0.119	0.174	0.219	0.094	0.243	0.346	0.153
VML180	0.118	0.110	0.176	0.201	0.054	0.240	0.334	0.196
VML183	0.148	0.130	0.144	0.173	0.118	0.207	0.334	0.191
VML184	0.167	0.149	0.134	0.192	0.117	0.223	0.362	0.215
VML188	0.223	0.188	0.177	0.084	0.216	0.112	0.248	0.311

	VML120	VML121	VML122	VML123	VML124	VML125	VML126	VML127
VML001	0.322	0.196	0.217	0.232	0.355	0.468	0.272	0.283
VML002	0.206	0.226	0.129	0.151	0.228	0.376	0.160	0.189

VML003	0.267	0.126	0.197	0.179	0.359	0.469	0.243	0.249
VML004	0.219	0.116	0.227	0.161	0.347	0.431	0.214	0.238
VML005	0.088	0.191	0.143	0.064	0.303	0.399	0.067	0.132
VML006	0.130	0.286	0.184	0.230	0.236	0.271	0.201	0.114
VML007	0.352	0.204	0.252	0.283	0.363	0.406	0.294	0.290
VML008	0.234	0.118	0.173	0.197	0.313	0.364	0.221	0.186
VML009	0.308	0.249	0.259	0.295	0.225	0.234	0.284	0.264
VML010	0.125	0.278	0.157	0.200	0.208	0.260	0.145	0.102
VML011	0.211	0.130	0.127	0.175	0.276	0.350	0.200	0.157
VML012	0.155	0.256	0.101	0.182	0.170	0.271	0.143	0.097
VML015	0.125	0.283	0.190	0.220	0.230	0.292	0.201	0.136
VML016	0.233	0.198	0.144	0.164	0.246	0.354	0.158	0.192
VML017	0.235	0.255	0.207	0.279	0.252	0.245	0.267	0.172
VML018	0.209	0.212	0.100	0.199	0.178	0.283	0.196	0.140
VML020	0.139	0.188	0.135	0.178	0.255	0.315	0.185	0.106
VML021	0.207	0.305	0.170	0.245	0.170	0.219	0.188	0.149
VML022	0.198	0.168	0.124	0.165	0.211	0.299	0.163	0.150
VML023	0.204	0.143	0.113	0.154	0.250	0.337	0.166	0.152
VML024	0.220	0.148	0.154	0.160	0.293	0.356	0.156	0.175
VML025	0.183	0.343	0.210	0.283	0.185	0.216	0.246	0.154
VML026	0.197	0.163	0.102	0.152	0.232	0.330	0.158	0.146
VML027	0.195	0.095	0.149	0.144	0.309	0.390	0.188	0.173
VML028	0.061	0.233	0.160	0.131	0.328	0.416	0.137	0.128
VML030	0.242	0.051	0.214	0.163	0.337	0.409	0.205	0.237
VML031	0.180	0.177	0.104	0.113	0.270	0.386	0.129	0.155
VML032	0.252	0.139	0.190	0.162	0.375	0.488	0.228	0.241
VML033	0.205	0.128	0.247	0.150	0.412	0.485	0.206	0.244
VML034	0.189	0.111	0.139	0.143	0.310	0.384	0.176	0.155
VML036	0.387	0.228	0.291	0.289	0.437	0.542	0.337	0.354
VML039	0.133	0.181	0.106	0.152	0.227	0.296	0.143	0.085
VML040	0.288	0.157	0.185	0.220	0.350	0.431	0.255	0.236
VML041	0.186	0.135	0.172	0.119	0.283	0.354	0.111	0.185
VML042	0.249	0.106	0.241	0.206	0.395	0.443	0.250	0.241
VML043	0.191	0.285	0.110	0.238	0.139	0.236	0.213	0.106
VML044	0.253	0.159	0.139	0.198	0.287	0.384	0.229	0.198
VML046	0.282	0.142	0.235	0.236	0.312	0.374	0.270	0.256
VML047	0.391	0.333	0.293	0.391	0.277	0.314	0.403	0.317
VML048	0.234	0.347	0.180	0.280	0.095	0.209	0.236	0.178
VML049	0.216	0.254	0.168	0.228	0.184	0.219	0.183	0.159
VML050	0.217	0.248	0.184	0.264	0.266	0.283	0.260	0.150
VML051	0.199	0.312	0.170	0.275	0.178	0.200	0.240	0.122
VML052	0.152	0.262	0.221	0.126	0.388	0.502	0.153	0.218
VML053	0.124	0.192	0.180	0.148	0.265	0.340	0.155	0.156
VML054	0.261	0.193	0.172	0.245	0.301	0.341	0.254	0.185

VML055	0.207	0.220	0.161	0.137	0.336	0.439	0.139	0.192
VML056	0.160	0.192	0.100	0.172	0.225	0.326	0.192	0.114
VML057	0.206	0.236	0.191	0.237	0.195	0.254	0.234	0.180
VML058	0.091	0.235	0.158	0.168	0.280	0.329	0.140	0.092
VML059	0.210	0.153	0.146	0.165	0.244	0.318	0.165	0.165
VML061	0.239	0.371	0.244	0.332	0.188	0.186	0.301	0.196
VML063	0.197	0.175	0.155	0.182	0.228	0.272	0.161	0.153
VML064	0.213	0.149	0.127	0.173	0.263	0.335	0.181	0.160
VML065	0.196	0.202	0.086	0.198	0.223	0.305	0.200	0.116
VML066	0.241	0.167	0.152	0.220	0.265	0.317	0.232	0.172
VML067	0.196	0.302	0.176	0.262	0.191	0.196	0.215	0.127
VML068	0.197	0.117	0.139	0.151	0.258	0.338	0.172	0.164
VML069	0.161	0.218	0.068	0.156	0.185	0.319	0.156	0.119
VML070	0.122	0.170	0.106	0.082	0.285	0.394	0.113	0.133
VML073	0.217	0.264	0.161	0.216	0.207	0.262	0.165	0.161
VML074	0.099	0.203	0.166	0.120	0.331	0.434	0.163	0.159
VML075	0.299	0.270	0.215	0.306	0.219	0.291	0.320	0.238
VML076	0.256	0.106	0.195	0.152	0.314	0.416	0.193	0.241
VML080	0.201	0.117	0.152	0.175	0.298	0.354	0.199	0.160
VML081	0.201	0.232	0.166	0.244	0.234	0.265	0.241	0.139
VML083	0.138	0.275	0.138	0.203	0.192	0.255	0.153	0.096
VML084	0.240	0.231	0.175	0.262	0.238	0.250	0.253	0.161
VML085	0.120	0.179	0.133	0.106	0.301	0.370	0.088	0.116
VML086	0.204	0.358	0.171	0.260	0.158	0.276	0.213	0.162
VML087	0.211	0.146	0.187	0.192	0.266	0.320	0.208	0.186
VML088	0.157	0.174	0.187	0.181	0.299	0.336	0.185	0.150
VML089	0.076	0.226	0.175	0.154	0.288	0.364	0.157	0.132
VML090	0.105	0.282	0.182	0.191	0.269	0.315	0.133	0.121
VML091	0.194	0.204	0.121	0.189	0.173	0.277	0.189	0.144
VML092	0.131	0.171	0.131	0.095	0.262	0.342	0.062	0.128
VML093	0.224	0.208	0.160	0.235	0.217	0.252	0.230	0.156
VML094	0.147	0.302	0.139	0.218	0.186	0.260	0.167	0.097
VML095	0.132	0.219	0.083	0.178	0.215	0.292	0.169	0.053
VML096	0.250	0.285	0.176	0.271	0.113	0.187	0.247	0.183
VML097	0.175	0.152	0.131	0.164	0.260	0.312	0.163	0.123
VML099	0.169	0.166	0.186	0.175	0.282	0.304	0.154	0.156
VML100	0.117	0.163	0.162	0.114	0.303	0.358	0.097	0.132
VML102	0.135	0.159	0.085	0.127	0.244	0.337	0.144	0.099
VML104	0.141	0.294	0.150	0.213	0.150	0.246	0.173	0.117
VML106	0.290	0.181	0.189	0.232	0.270	0.368	0.261	0.239
VML107	0.218	0.205	0.104	0.189	0.196	0.304	0.188	0.152
VML110	0.200	0.260	0.121	0.225	0.127	0.246	0.213	0.135
VML111	0.287	0.330	0.208	0.319	0.115	0.160	0.293	0.211
VML112	0.229	0.159	0.194	0.208	0.266	0.285	0.198	0.189

VML113	0.177	0.123	0.172	0.103	0.338	0.444	0.173	0.197
VML114	0.357	0.191	0.316	0.321	0.429	0.454	0.360	0.325
VML116	0.246	0.330	0.192	0.301	0.105	0.162	0.271	0.177
VML117	0.090	0.235	0.118	0.116	0.267	0.367	0.086	0.106
VML118	0.317	0.113	0.267	0.251	0.374	0.411	0.281	0.286
VML119	0.348	0.226	0.249	0.295	0.299	0.396	0.331	0.301
VML120		0.247	0.151	0.140	0.280	0.369	0.127	0.108
VML121	0.081		0.225	0.156	0.371	0.439	0.203	0.246
VML122	0.127	0.076		0.164	0.222	0.339	0.169	0.081
VML123	0.197	0.169	0.137		0.319	0.426	0.082	0.178
VML124	0.138	0.149	0.172	0.264		0.201	0.289	0.230
VML125	0.256	0.275	0.292	0.304	0.188		0.378	0.311
VML126	0.148	0.104	0.063	0.104	0.218	0.322		0.161
VML127	0.173	0.143	0.160	0.125	0.240	0.279	0.142	
VML128	0.077	0.077	0.116	0.190	0.101	0.219	0.146	0.176
VML129	0.128	0.131	0.128	0.130	0.168	0.207	0.142	0.122
VML130	0.082	0.068	0.122	0.226	0.099	0.252	0.162	0.189
VML131	0.266	0.247	0.214	0.150	0.288	0.279	0.210	0.175
VML132	0.126	0.081	0.090	0.113	0.207	0.300	0.066	0.110
VML133	0.201	0.211	0.232	0.264	0.134	0.082	0.267	0.223
VML134	0.360	0.312	0.271	0.194	0.431	0.485	0.227	0.257
VML136	0.121	0.145	0.188	0.177	0.200	0.230	0.182	0.127
VML137	0.141	0.154	0.173	0.258	0.050	0.184	0.220	0.250
VML138	0.232	0.195	0.137	0.113	0.276	0.337	0.129	0.177
VML139	0.099	0.102	0.125	0.135	0.192	0.260	0.127	0.149
VML140	0.426	0.406	0.387	0.271	0.490	0.464	0.350	0.279
VML141	0.229	0.181	0.132	0.164	0.302	0.415	0.110	0.220
VML143	0.147	0.135	0.142	0.105	0.205	0.226	0.143	0.086
VML144	0.457	0.415	0.390	0.290	0.530	0.546	0.347	0.310
VML145	0.069	0.108	0.133	0.214	0.101	0.231	0.167	0.215
VML146	0.111	0.063	0.052	0.118	0.169	0.267	0.075	0.132
VML147	0.223	0.165	0.146	0.179	0.303	0.419	0.116	0.191
VML148	0.032	0.077	0.121	0.191	0.159	0.280	0.133	0.172
VML150	0.105	0.079	0.066	0.110	0.158	0.244	0.080	0.122
VML151	0.159	0.152	0.133	0.075	0.235	0.277	0.117	0.110
VML152	0.167	0.183	0.213	0.333	0.169	0.349	0.248	0.315
VML153	0.129	0.137	0.166	0.286	0.113	0.291	0.211	0.271
VML154	0.274	0.237	0.215	0.121	0.363	0.412	0.166	0.180
VML155	0.202	0.136	0.130	0.145	0.267	0.363	0.101	0.144
VML156	0.156	0.131	0.133	0.208	0.102	0.189	0.184	0.201
VML157	0.136	0.111	0.115	0.152	0.147	0.208	0.143	0.118
VML158	0.184	0.205	0.251	0.363	0.161	0.325	0.288	0.329
VML159	0.133	0.153	0.145	0.132	0.187	0.218	0.156	0.174
VML160	0.152	0.127	0.127	0.096	0.226	0.278	0.111	0.058

VML161	0.151	0.106	0.105	0.149	0.219	0.330	0.077	0.160
VML162	0.120	0.117	0.089	0.118	0.209	0.299	0.091	0.162
VML164	0.118	0.084	0.097	0.125	0.217	0.317	0.069	0.108
VML165	0.135	0.139	0.126	0.156	0.134	0.185	0.155	0.175
VML168	0.114	0.070	0.087	0.137	0.200	0.307	0.079	0.145
VML170	0.120	0.086	0.134	0.173	0.158	0.229	0.153	0.118
VML171	0.157	0.164	0.172	0.207	0.120	0.132	0.211	0.203
VML173	0.306	0.278	0.258	0.137	0.367	0.359	0.224	0.162
VML174	0.062	0.033	0.091	0.185	0.129	0.262	0.124	0.165
VML175	0.162	0.113	0.095	0.137	0.250	0.364	0.063	0.164
VML176	0.213	0.216	0.212	0.124	0.274	0.255	0.196	0.125
VML178	0.215	0.191	0.193	0.100	0.306	0.339	0.153	0.119
VML179	0.118	0.072	0.078	0.188	0.110	0.247	0.129	0.184
VML180	0.059	0.067	0.094	0.175	0.111	0.234	0.125	0.165
VML183	0.112	0.051	0.063	0.167	0.153	0.278	0.100	0.139
VML184	0.097	0.048	0.066	0.182	0.155	0.295	0.103	0.161
VML188	0.221	0.190	0.181	0.100	0.304	0.351	0.134	0.095

	VML128	VML129	VML130	VML131	VML132	VML133	VML134	VML136
VML001	0.171	0.326	0.127	0.237	0.283	0.288	0.172	0.347
VML002	0.065	0.206	0.182	0.260	0.190	0.293	0.110	0.334
VML003	0.173	0.318	0.096	0.193	0.274	0.262	0.157	0.286
VML004	0.211	0.305	0.204	0.201	0.279	0.246	0.191	0.205
VML005	0.143	0.221	0.240	0.296	0.156	0.342	0.115	0.304
VML006	0.230	0.143	0.305	0.276	0.147	0.299	0.226	0.232
VML007	0.222	0.321	0.214	0.231	0.276	0.265	0.198	0.304
VML008	0.178	0.250	0.161	0.142	0.205	0.202	0.145	0.179
VML009	0.234	0.231	0.274	0.167	0.268	0.119	0.246	0.152
VML010	0.183	0.108	0.299	0.297	0.097	0.317	0.181	0.278
VML011	0.136	0.218	0.130	0.137	0.178	0.199	0.108	0.203
VML012	0.110	0.084	0.242	0.257	0.074	0.283	0.133	0.282
VML015	0.226	0.159	0.299	0.270	0.177	0.291	0.230	0.229
VML016	0.063	0.208	0.191	0.243	0.175	0.274	0.088	0.307
VML017	0.244	0.185	0.266	0.179	0.192	0.196	0.236	0.130
VML018	0.096	0.138	0.163	0.162	0.140	0.194	0.123	0.232
VML020	0.176	0.177	0.205	0.183	0.152	0.228	0.159	0.175
VML021	0.176	0.090	0.303	0.299	0.106	0.308	0.192	0.303
VML022	0.089	0.166	0.175	0.166	0.153	0.195	0.102	0.208
VML023	0.086	0.195	0.144	0.168	0.157	0.215	0.072	0.230
VML024	0.121	0.224	0.194	0.227	0.163	0.267	0.087	0.261
VML025	0.255	0.113	0.337	0.296	0.170	0.303	0.266	0.260
VML026	0.065	0.180	0.148	0.182	0.146	0.224	0.070	0.244
VML027	0.152	0.251	0.139	0.162	0.206	0.227	0.114	0.211
VML028	0.200	0.235	0.260	0.318	0.166	0.370	0.171	0.319

VML030	0.178	0.295	0.167	0.180	0.261	0.219	0.150	0.208
VML031	0.058	0.211	0.160	0.248	0.154	0.295	0.050	0.310
VML032	0.174	0.323	0.112	0.237	0.263	0.306	0.147	0.321
VML033	0.245	0.342	0.240	0.281	0.283	0.335	0.203	0.272
VML034	0.144	0.238	0.149	0.181	0.178	0.244	0.107	0.218
VML036	0.252	0.407	0.173	0.285	0.357	0.339	0.241	0.397
VML039	0.130	0.146	0.203	0.196	0.113	0.234	0.115	0.201
VML040	0.175	0.297	0.116	0.198	0.240	0.263	0.144	0.296
VML041	0.125	0.229	0.215	0.247	0.189	0.272	0.091	0.260
VML042	0.248	0.328	0.211	0.206	0.275	0.265	0.204	0.202
VML043	0.154	0.052	0.238	0.224	0.096	0.249	0.178	0.260
VML044	0.128	0.245	0.082	0.155	0.206	0.217	0.117	0.263
VML046	0.216	0.290	0.181	0.098	0.284	0.132	0.208	0.140
VML047	0.305	0.292	0.257	0.126	0.329	0.113	0.324	0.221
VML048	0.192	0.072	0.307	0.300	0.152	0.302	0.228	0.327
VML049	0.157	0.124	0.266	0.249	0.131	0.252	0.164	0.253
VML050	0.232	0.186	0.241	0.182	0.173	0.218	0.222	0.161
VML051	0.219	0.079	0.293	0.253	0.118	0.268	0.228	0.242
VML052	0.224	0.310	0.299	0.383	0.242	0.438	0.204	0.398
VML053	0.186	0.208	0.248	0.231	0.199	0.259	0.176	0.200
VML054	0.193	0.232	0.182	0.161	0.187	0.216	0.173	0.211
VML055	0.135	0.260	0.231	0.325	0.178	0.377	0.110	0.374
VML056	0.136	0.167	0.159	0.152	0.155	0.207	0.140	0.200
VML057	0.203	0.173	0.240	0.166	0.211	0.166	0.212	0.149
VML058	0.195	0.175	0.278	0.283	0.120	0.321	0.174	0.251
VML059	0.107	0.192	0.182	0.175	0.165	0.206	0.110	0.204
VML061	0.291	0.142	0.353	0.288	0.212	0.286	0.304	0.251
VML063	0.138	0.170	0.219	0.196	0.153	0.212	0.123	0.198
VML064	0.112	0.204	0.144	0.178	0.159	0.219	0.091	0.234
VML065	0.120	0.154	0.150	0.169	0.121	0.216	0.120	0.233
VML066	0.163	0.210	0.155	0.117	0.183	0.170	0.147	0.181
VML067	0.212	0.089	0.304	0.274	0.107	0.285	0.213	0.256
VML068	0.117	0.210	0.147	0.146	0.184	0.188	0.102	0.190
VML069	0.066	0.138	0.169	0.214	0.129	0.250	0.102	0.276
VML070	0.111	0.216	0.178	0.256	0.159	0.307	0.077	0.299
VML073	0.143	0.140	0.273	0.284	0.118	0.299	0.149	0.303
VML074	0.195	0.258	0.221	0.282	0.208	0.337	0.172	0.296
VML075	0.231	0.222	0.198	0.085	0.258	0.093	0.252	0.178
VML076	0.127	0.285	0.150	0.199	0.252	0.239	0.121	0.266
VML080	0.167	0.232	0.160	0.152	0.187	0.209	0.129	0.181
VML081	0.208	0.165	0.226	0.158	0.167	0.190	0.203	0.144
VML083	0.164	0.088	0.275	0.284	0.075	0.303	0.170	0.280
VML084	0.206	0.173	0.225	0.154	0.168	0.180	0.197	0.161
VML085	0.144	0.207	0.234	0.280	0.125	0.326	0.095	0.286

VML086	0.191	0.098	0.320	0.342	0.132	0.361	0.225	0.369
VML087	0.180	0.222	0.198	0.134	0.215	0.162	0.170	0.118
VML088	0.209	0.221	0.244	0.211	0.188	0.249	0.182	0.159
VML089	0.206	0.210	0.266	0.272	0.183	0.311	0.191	0.245
VML090	0.211	0.168	0.322	0.337	0.125	0.365	0.193	0.311
VML091	0.111	0.141	0.178	0.152	0.157	0.175	0.139	0.198
VML092	0.108	0.187	0.228	0.261	0.132	0.296	0.078	0.269
VML093	0.178	0.164	0.207	0.134	0.168	0.157	0.175	0.148
VML094	0.174	0.077	0.294	0.303	0.070	0.327	0.185	0.306
VML095	0.140	0.121	0.203	0.205	0.085	0.248	0.136	0.224
VML096	0.178	0.110	0.253	0.196	0.175	0.187	0.208	0.226
VML097	0.141	0.182	0.191	0.177	0.138	0.220	0.114	0.185
VML099	0.187	0.206	0.250	0.232	0.169	0.253	0.153	0.190
VML100	0.166	0.215	0.246	0.269	0.152	0.306	0.123	0.249
VML102	0.110	0.174	0.165	0.185	0.135	0.237	0.090	0.221
VML104	0.173	0.075	0.286	0.275	0.124	0.290	0.196	0.271
VML106	0.154	0.256	0.130	0.111	0.249	0.157	0.172	0.226
VML107	0.076	0.157	0.162	0.176	0.146	0.211	0.108	0.251
VML110	0.137	0.094	0.212	0.182	0.143	0.201	0.173	0.228
VML111	0.222	0.123	0.284	0.216	0.198	0.202	0.252	0.247
VML112	0.179	0.212	0.227	0.178	0.194	0.191	0.158	0.159
VML113	0.165	0.283	0.170	0.229	0.237	0.290	0.134	0.271
VML114	0.324	0.385	0.245	0.175	0.355	0.222	0.292	0.185
VML116	0.220	0.082	0.292	0.232	0.172	0.227	0.248	0.242
VML117	0.137	0.181	0.252	0.307	0.117	0.350	0.120	0.326
VML118	0.242	0.334	0.207	0.163	0.302	0.197	0.214	0.185
VML119	0.223	0.309	0.150	0.103	0.317	0.132	0.240	0.244
VML120	0.187	0.191	0.267	0.306	0.148	0.346	0.173	0.298
VML121	0.194	0.318	0.185	0.215	0.268	0.261	0.150	0.238
VML122	0.100	0.146	0.168	0.222	0.099	0.276	0.104	0.286
VML123	0.131	0.255	0.219	0.285	0.199	0.331	0.105	0.305
VML124	0.224	0.122	0.316	0.280	0.222	0.266	0.273	0.313
VML125	0.353	0.214	0.430	0.345	0.293	0.306	0.372	0.309
VML126	0.137	0.217	0.267	0.312	0.164	0.345	0.120	0.311
VML127	0.154	0.124	0.229	0.250	0.063	0.296	0.144	0.269
VML128		0.181	0.170	0.227	0.152	0.266	0.072	0.291
VML129	0.128		0.283	0.267	0.109	0.280	0.208	0.282
VML130	0.069	0.153		0.168	0.252	0.234	0.167	0.288
VML131	0.260	0.144	0.281		0.275	0.093	0.231	0.163
VML132	0.121	0.137	0.144	0.226		0.313	0.149	0.295
VML133	0.169	0.155	0.186	0.241	0.245		0.280	0.153
VML134	0.358	0.312	0.377	0.270	0.248	0.440		0.284
VML136	0.128	0.128	0.164	0.245	0.136	0.193	0.347	
VML137	0.094	0.172	0.112	0.294	0.208	0.142	0.429	0.201

VML138	0.231	0.155	0.242	0.127	0.172	0.285	0.202	0.252
VML139	0.098	0.122	0.143	0.238	0.093	0.219	0.302	0.106
VML140	0.433	0.332	0.456	0.239	0.352	0.436	0.264	0.362
VML141	0.237	0.229	0.238	0.259	0.155	0.357	0.190	0.277
VML143	0.143	0.065	0.174	0.149	0.119	0.178	0.281	0.112
VML144	0.460	0.389	0.476	0.315	0.352	0.505	0.167	0.412
VML145	0.059	0.142	0.089	0.278	0.156	0.186	0.385	0.155
VML146	0.095	0.109	0.118	0.207	0.064	0.211	0.271	0.151
VML147	0.231	0.238	0.224	0.282	0.132	0.356	0.196	0.257
VML148	0.087	0.141	0.093	0.273	0.112	0.226	0.346	0.124
VML150	0.089	0.078	0.121	0.186	0.074	0.190	0.281	0.131
VML151	0.173	0.079	0.198	0.131	0.123	0.230	0.248	0.150
VML152	0.179	0.272	0.140	0.398	0.246	0.294	0.466	0.274
VML153	0.122	0.218	0.088	0.344	0.207	0.236	0.431	0.233
VML154	0.276	0.239	0.303	0.240	0.165	0.370	0.116	0.246
VML155	0.189	0.204	0.196	0.256	0.089	0.305	0.205	0.209
VML156	0.106	0.136	0.120	0.230	0.176	0.130	0.367	0.199
VML157	0.118	0.061	0.127	0.159	0.135	0.141	0.317	0.148
VML158	0.186	0.287	0.149	0.421	0.273	0.276	0.508	0.273
VML159	0.128	0.086	0.177	0.191	0.149	0.189	0.321	0.137
VML160	0.162	0.087	0.176	0.148	0.101	0.221	0.248	0.135
VML161	0.137	0.183	0.158	0.269	0.058	0.279	0.257	0.174
VML162	0.145	0.110	0.163	0.197	0.112	0.247	0.270	0.172
VML164	0.137	0.138	0.145	0.227	0.043	0.258	0.252	0.139
VML165	0.109	0.066	0.146	0.176	0.159	0.142	0.340	0.158
VML168	0.108	0.154	0.130	0.254	0.043	0.254	0.269	0.147
VML170	0.094	0.130	0.111	0.238	0.103	0.170	0.324	0.116
VML171	0.122	0.110	0.154	0.212	0.197	0.089	0.388	0.172
VML173	0.303	0.216	0.332	0.153	0.221	0.326	0.186	0.248
VML174	0.054	0.134	0.051	0.261	0.102	0.201	0.337	0.142
VML175	0.170	0.185	0.176	0.254	0.079	0.306	0.222	0.202
VML176	0.219	0.113	0.253	0.123	0.185	0.225	0.285	0.153
VML178	0.216	0.183	0.250	0.220	0.123	0.301	0.204	0.160
VML179	0.071	0.139	0.073	0.247	0.127	0.186	0.336	0.182
VML180	0.046	0.105	0.069	0.238	0.115	0.178	0.344	0.131
VML183	0.108	0.123	0.086	0.220	0.100	0.208	0.301	0.171
VML184	0.109	0.139	0.080	0.244	0.106	0.227	0.313	0.178
VML188	0.228	0.173	0.248	0.186	0.128	0.303	0.186	0.182

	VML137	VML138	VML139	VML140	VML141	VML143	VML144	VML145
VML001	0.150	0.219	0.287	0.237	0.205	0.208	0.115	0.373
VML002	0.203	0.183	0.182	0.232	0.169	0.173	0.197	0.230
VML003	0.190	0.243	0.254	0.133	0.219	0.174	0.023	0.338
VML004	0.258	0.283	0.211	0.097	0.278	0.161	0.159	0.274

VML005	0.236	0.212	0.079	0.164	0.145	0.144	0.227	0.139
VML006	0.321	0.221	0.172	0.232	0.268	0.195	0.329	0.181
VML007	0.091	0.180	0.288	0.269	0.231	0.186	0.210	0.385
VML008	0.161	0.168	0.206	0.122	0.213	0.090	0.156	0.298
VML009	0.255	0.219	0.263	0.279	0.333	0.188	0.285	0.316
VML010	0.276	0.174	0.119	0.252	0.213	0.172	0.320	0.129
VML011	0.150	0.143	0.189	0.121	0.189	0.077	0.144	0.277
VML012	0.228	0.113	0.137	0.239	0.168	0.149	0.268	0.177
VML015	0.336	0.246	0.179	0.224	0.284	0.205	0.317	0.177
VML016	0.136	0.126	0.171	0.233	0.138	0.135	0.189	0.245
VML017	0.278	0.191	0.237	0.221	0.295	0.172	0.295	0.291
VML018	0.186	0.099	0.191	0.204	0.194	0.122	0.201	0.256
VML020	0.244	0.183	0.162	0.128	0.227	0.116	0.224	0.221
VML021	0.254	0.129	0.172	0.301	0.213	0.190	0.332	0.196
VML022	0.166	0.116	0.157	0.180	0.186	0.081	0.182	0.232
VML023	0.129	0.112	0.162	0.158	0.157	0.067	0.150	0.250
VML024	0.099	0.112	0.151	0.190	0.129	0.079	0.183	0.249
VML025	0.358	0.237	0.222	0.293	0.310	0.242	0.373	0.211
VML026	0.139	0.104	0.159	0.174	0.149	0.083	0.161	0.241
VML027	0.177	0.196	0.181	0.069	0.201	0.090	0.125	0.265
VML028	0.284	0.245	0.135	0.171	0.184	0.187	0.265	0.168
VML030	0.171	0.232	0.199	0.129	0.240	0.110	0.122	0.285
VML031	0.153	0.145	0.146	0.185	0.099	0.124	0.164	0.224
VML032	0.187	0.244	0.242	0.139	0.188	0.180	0.070	0.326
VML033	0.267	0.305	0.203	0.100	0.255	0.183	0.193	0.270
VML034	0.164	0.165	0.170	0.094	0.168	0.082	0.137	0.261
VML036	0.193	0.293	0.352	0.277	0.264	0.270	0.147	0.442
VML039	0.206	0.138	0.121	0.151	0.185	0.080	0.218	0.190
VML040	0.108	0.177	0.256	0.184	0.183	0.155	0.125	0.352
VML041	0.146	0.177	0.100	0.186	0.174	0.077	0.199	0.188
VML042	0.229	0.268	0.235	0.090	0.267	0.155	0.177	0.320
VML043	0.258	0.118	0.202	0.250	0.219	0.178	0.284	0.238
VML044	0.131	0.149	0.231	0.169	0.182	0.130	0.116	0.318
VML046	0.228	0.247	0.259	0.160	0.303	0.156	0.164	0.332
VML047	0.328	0.271	0.390	0.318	0.395	0.288	0.306	0.450
VML048	0.301	0.172	0.228	0.330	0.261	0.238	0.351	0.234
VML049	0.204	0.107	0.161	0.268	0.212	0.140	0.291	0.212
VML050	0.271	0.180	0.234	0.200	0.269	0.170	0.275	0.293
VML051	0.298	0.159	0.215	0.273	0.262	0.202	0.336	0.242
VML052	0.318	0.307	0.181	0.220	0.194	0.249	0.274	0.199
VML053	0.278	0.240	0.137	0.152	0.255	0.144	0.242	0.170
VML054	0.173	0.129	0.240	0.186	0.211	0.136	0.203	0.328
VML055	0.180	0.183	0.164	0.230	0.041	0.180	0.218	0.237
VML056	0.235	0.170	0.185	0.132	0.215	0.127	0.187	0.243

VML057	0.283	0.219	0.211	0.209	0.301	0.165	0.266	0.246
VML058	0.271	0.196	0.114	0.189	0.200	0.153	0.285	0.154
VML059	0.156	0.123	0.159	0.177	0.182	0.078	0.174	0.242
VML061	0.381	0.257	0.274	0.322	0.354	0.271	0.397	0.272
VML063	0.165	0.125	0.136	0.198	0.202	0.066	0.228	0.213
VML064	0.113	0.104	0.172	0.172	0.159	0.071	0.163	0.263
VML065	0.175	0.086	0.189	0.181	0.173	0.111	0.197	0.264
VML066	0.162	0.129	0.217	0.165	0.217	0.101	0.180	0.302
VML067	0.275	0.145	0.186	0.280	0.244	0.186	0.340	0.214
VML068	0.159	0.156	0.163	0.131	0.199	0.059	0.145	0.246
VML069	0.208	0.138	0.160	0.197	0.170	0.136	0.204	0.212
VML070	0.199	0.191	0.121	0.144	0.140	0.124	0.184	0.189
VML073	0.194	0.098	0.153	0.281	0.172	0.155	0.292	0.205
VML074	0.286	0.266	0.166	0.125	0.216	0.184	0.221	0.204
VML075	0.286	0.221	0.307	0.244	0.330	0.217	0.249	0.362
VML076	0.146	0.210	0.204	0.172	0.205	0.130	0.094	0.287
VML080	0.169	0.169	0.181	0.103	0.207	0.071	0.165	0.269
VML081	0.261	0.174	0.215	0.186	0.266	0.149	0.260	0.271
VML083	0.256	0.143	0.133	0.254	0.196	0.165	0.306	0.158
VML084	0.222	0.135	0.227	0.214	0.255	0.141	0.260	0.295
VML085	0.192	0.168	0.075	0.166	0.120	0.107	0.230	0.163
VML086	0.318	0.190	0.214	0.330	0.230	0.254	0.358	0.207
VML087	0.222	0.201	0.189	0.143	0.260	0.105	0.192	0.259
VML088	0.253	0.216	0.157	0.133	0.246	0.121	0.240	0.219
VML089	0.304	0.252	0.142	0.159	0.245	0.175	0.268	0.161
VML090	0.291	0.213	0.106	0.253	0.210	0.190	0.335	0.103
VML091	0.214	0.139	0.181	0.191	0.221	0.120	0.203	0.238
VML092	0.183	0.158	0.053	0.179	0.139	0.089	0.219	0.144
VML093	0.214	0.137	0.207	0.195	0.248	0.119	0.236	0.273
VML094	0.279	0.155	0.153	0.271	0.201	0.193	0.325	0.164
VML095	0.229	0.129	0.153	0.171	0.185	0.121	0.235	0.210
VML096	0.257	0.147	0.231	0.279	0.274	0.185	0.293	0.270
VML097	0.173	0.127	0.143	0.143	0.182	0.054	0.197	0.228
VML099	0.202	0.178	0.116	0.179	0.219	0.080	0.250	0.192
VML100	0.208	0.190	0.065	0.156	0.171	0.096	0.236	0.153
VML102	0.196	0.153	0.135	0.117	0.173	0.084	0.178	0.207
VML104	0.300	0.189	0.160	0.259	0.243	0.193	0.314	0.157
VML106	0.187	0.186	0.263	0.199	0.248	0.163	0.132	0.341
VML107	0.167	0.093	0.189	0.208	0.174	0.122	0.188	0.259
VML110	0.253	0.141	0.205	0.231	0.237	0.166	0.252	0.245
VML111	0.291	0.167	0.276	0.318	0.307	0.228	0.333	0.311
VML112	0.170	0.156	0.170	0.193	0.236	0.077	0.227	0.251
VML113	0.227	0.252	0.180	0.078	0.209	0.148	0.136	0.247
VML114	0.278	0.318	0.342	0.195	0.364	0.233	0.229	0.425

VML116	0.311	0.178	0.253	0.302	0.300	0.225	0.339	0.274
VML117	0.242	0.189	0.093	0.204	0.137	0.161	0.260	0.131
VML118	0.181	0.247	0.268	0.183	0.289	0.156	0.169	0.359
VML119	0.240	0.254	0.330	0.243	0.324	0.226	0.169	0.402
VML120	0.293	0.233	0.118	0.191	0.204	0.182	0.279	0.130
VML121	0.163	0.243	0.198	0.123	0.227	0.119	0.127	0.290
VML122	0.212	0.128	0.171	0.179	0.144	0.143	0.209	0.229
VML123	0.218	0.229	0.108	0.154	0.156	0.142	0.185	0.173
VML124	0.345	0.226	0.283	0.351	0.326	0.274	0.361	0.286
VML125	0.402	0.286	0.350	0.434	0.421	0.333	0.472	0.356
VML126	0.226	0.206	0.047	0.209	0.152	0.145	0.247	0.111
VML127	0.249	0.146	0.147	0.189	0.171	0.150	0.262	0.192
VML128	0.176	0.138	0.153	0.198	0.139	0.128	0.175	0.219
VML129	0.293	0.152	0.204	0.285	0.242	0.207	0.326	0.219
VML130	0.196	0.215	0.271	0.170	0.228	0.183	0.107	0.349
VML131	0.249	0.228	0.301	0.200	0.315	0.192	0.196	0.371
VML132	0.236	0.111	0.152	0.234	0.152	0.161	0.284	0.199
VML133	0.286	0.261	0.329	0.270	0.369	0.230	0.260	0.389
VML134	0.136	0.138	0.127	0.161	0.109	0.089	0.163	0.214
VML136	0.310	0.278	0.285	0.231	0.365	0.208	0.289	0.340
VML137		0.163	0.222	0.233	0.174	0.139	0.189	0.319
VML138	0.277		0.197	0.246	0.158	0.141	0.249	0.270
VML139	0.176	0.203		0.204	0.168	0.126	0.260	0.103
VML140	0.496	0.294	0.378		0.227	0.144	0.148	0.279
VML141	0.301	0.139	0.205	0.374		0.173	0.227	0.243
VML143	0.204	0.160	0.106	0.303	0.224		0.179	0.220
VML144	0.533	0.305	0.400	0.174	0.331	0.350		0.343
VML145	0.087	0.245	0.116	0.460	0.248	0.171	0.493	
VML146	0.165	0.151	0.084	0.371	0.157	0.105	0.380	0.123
VML147	0.307	0.183	0.203	0.372	0.085	0.222	0.318	0.256
VML148	0.161	0.229	0.096	0.423	0.212	0.155	0.446	0.085
VML150	0.158	0.148	0.090	0.358	0.178	0.090	0.383	0.116
VML151	0.235	0.116	0.124	0.281	0.185	0.074	0.326	0.187
VML152	0.178	0.339	0.243	0.574	0.297	0.301	0.584	0.155
VML153	0.116	0.292	0.196	0.530	0.269	0.247	0.546	0.102
VML154	0.358	0.184	0.206	0.257	0.170	0.200	0.214	0.304
VML155	0.269	0.183	0.166	0.351	0.136	0.178	0.313	0.228
VML156	0.093	0.214	0.162	0.437	0.256	0.155	0.465	0.129
VML157	0.158	0.160	0.141	0.352	0.225	0.086	0.395	0.148
VML158	0.172	0.378	0.260	0.597	0.349	0.314	0.616	0.170
VML159	0.169	0.184	0.090	0.363	0.234	0.099	0.413	0.126
VML160	0.233	0.133	0.128	0.283	0.186	0.067	0.317	0.190
VML161	0.220	0.201	0.129	0.390	0.155	0.173	0.375	0.169
VML162	0.204	0.131	0.104	0.358	0.145	0.123	0.379	0.141

VML164	0.223	0.169	0.107	0.347	0.148	0.126	0.351	0.162
VML165	0.127	0.175	0.130	0.381	0.240	0.112	0.433	0.114
VML168	0.194	0.190	0.080	0.387	0.154	0.140	0.382	0.137
VML170	0.161	0.221	0.110	0.388	0.238	0.108	0.407	0.142
VML171	0.106	0.229	0.156	0.418	0.293	0.135	0.473	0.131
VML173	0.368	0.185	0.247	0.146	0.262	0.176	0.194	0.334
VML174	0.129	0.213	0.097	0.427	0.202	0.145	0.442	0.079
VML175	0.249	0.162	0.136	0.371	0.088	0.173	0.346	0.190
VML176	0.274	0.181	0.173	0.236	0.268	0.093	0.323	0.239
VML178	0.301	0.197	0.143	0.266	0.206	0.134	0.271	0.249
VML179	0.109	0.198	0.134	0.434	0.203	0.158	0.447	0.099
VML180	0.113	0.203	0.100	0.415	0.215	0.133	0.446	0.058
VML183	0.166	0.166	0.137	0.391	0.171	0.137	0.401	0.135
VML184	0.168	0.180	0.137	0.412	0.166	0.157	0.419	0.123
VML188	0.310	0.162	0.179	0.234	0.186	0.142	0.245	0.260

	VML146	VML147	VML148	VML150	VML151	VML152	VML153	VML154
VML001	0.211	0.256	0.240	0.169	0.184	0.286	0.270	0.186
VML002	0.160	0.164	0.129	0.149	0.250	0.169	0.173	0.111
VML003	0.138	0.212	0.182	0.150	0.190	0.254	0.230	0.155
VML004	0.154	0.202	0.145	0.186	0.237	0.266	0.229	0.176
VML005	0.101	0.047	0.090	0.209	0.258	0.117	0.107	0.111
VML006	0.236	0.187	0.184	0.233	0.300	0.244	0.273	0.203
VML007	0.284	0.285	0.293	0.188	0.078	0.356	0.338	0.215
VML008	0.181	0.191	0.185	0.118	0.103	0.278	0.266	0.137
VML009	0.312	0.302	0.251	0.171	0.214	0.373	0.363	0.237
VML010	0.227	0.153	0.168	0.220	0.280	0.208	0.234	0.170
VML011	0.155	0.164	0.153	0.076	0.121	0.243	0.238	0.093
VML012	0.198	0.147	0.146	0.160	0.242	0.191	0.222	0.124
VML015	0.225	0.193	0.164	0.229	0.318	0.238	0.264	0.205
VML016	0.186	0.176	0.164	0.133	0.182	0.220	0.213	0.109
VML017	0.271	0.247	0.234	0.185	0.220	0.326	0.340	0.216
VML018	0.192	0.184	0.153	0.064	0.180	0.237	0.253	0.109
VML020	0.163	0.149	0.132	0.146	0.214	0.224	0.235	0.132
VML021	0.271	0.205	0.215	0.213	0.266	0.252	0.280	0.189
VML022	0.175	0.168	0.131	0.074	0.157	0.236	0.234	0.095
VML023	0.153	0.152	0.137	0.070	0.128	0.225	0.219	0.070
VML024	0.179	0.158	0.174	0.141	0.114	0.237	0.221	0.108
VML025	0.290	0.241	0.226	0.254	0.340	0.281	0.320	0.244
VML026	0.153	0.150	0.131	0.075	0.148	0.212	0.212	0.070
VML027	0.120	0.149	0.130	0.115	0.157	0.228	0.209	0.098
VML028	0.124	0.079	0.133	0.246	0.297	0.114	0.135	0.157
VML030	0.167	0.201	0.161	0.148	0.155	0.272	0.228	0.149
VML031	0.118	0.116	0.120	0.139	0.197	0.153	0.150	0.068

VML032	0.116	0.189	0.181	0.178	0.205	0.221	0.196	0.150
VML033	0.142	0.179	0.175	0.249	0.258	0.241	0.194	0.196
VML034	0.123	0.141	0.141	0.125	0.141	0.222	0.211	0.100
VML036	0.263	0.317	0.307	0.241	0.222	0.344	0.319	0.255
VML039	0.157	0.122	0.113	0.126	0.191	0.205	0.213	0.093
VML040	0.193	0.218	0.227	0.143	0.111	0.277	0.265	0.152
VML041	0.166	0.137	0.128	0.162	0.177	0.211	0.171	0.103
VML042	0.187	0.215	0.211	0.207	0.183	0.299	0.269	0.195
VML043	0.232	0.196	0.185	0.151	0.250	0.239	0.280	0.160
VML044	0.169	0.197	0.182	0.079	0.130	0.250	0.247	0.115
VML046	0.225	0.260	0.204	0.135	0.178	0.331	0.307	0.194
VML047	0.363	0.382	0.336	0.202	0.268	0.436	0.451	0.306
VML048	0.293	0.245	0.231	0.219	0.313	0.267	0.307	0.217
VML049	0.255	0.201	0.199	0.170	0.207	0.265	0.274	0.162
VML050	0.244	0.228	0.222	0.179	0.216	0.299	0.320	0.202
VML051	0.274	0.225	0.226	0.207	0.274	0.279	0.318	0.210
VML052	0.137	0.133	0.170	0.299	0.350	0.118	0.112	0.203
VML053	0.164	0.153	0.100	0.187	0.264	0.218	0.208	0.153
VML054	0.224	0.222	0.230	0.135	0.110	0.301	0.307	0.168
VML055	0.155	0.131	0.182	0.220	0.234	0.160	0.157	0.136
VML056	0.143	0.156	0.113	0.097	0.213	0.213	0.230	0.110
VML057	0.237	0.228	0.170	0.153	0.252	0.287	0.293	0.186
VML058	0.181	0.121	0.151	0.221	0.261	0.195	0.213	0.160
VML059	0.178	0.175	0.147	0.096	0.140	0.249	0.240	0.111
VML061	0.332	0.291	0.271	0.268	0.353	0.335	0.373	0.280
VML063	0.207	0.171	0.158	0.126	0.153	0.254	0.245	0.119
VML064	0.170	0.164	0.160	0.093	0.111	0.233	0.226	0.094
VML065	0.177	0.166	0.162	0.088	0.161	0.225	0.245	0.105
VML066	0.204	0.205	0.193	0.083	0.109	0.283	0.284	0.136
VML067	0.276	0.211	0.224	0.214	0.261	0.273	0.303	0.200
VML068	0.150	0.161	0.123	0.076	0.141	0.236	0.220	0.090
VML069	0.150	0.140	0.105	0.108	0.227	0.169	0.193	0.083
VML070	0.077	0.060	0.079	0.164	0.229	0.113	0.106	0.064
VML073	0.248	0.187	0.203	0.189	0.218	0.243	0.256	0.157
VML074	0.087	0.109	0.105	0.223	0.293	0.133	0.134	0.151
VML075	0.279	0.299	0.245	0.133	0.239	0.348	0.363	0.229
VML076	0.161	0.201	0.157	0.129	0.164	0.255	0.217	0.131
VML080	0.160	0.162	0.157	0.116	0.129	0.250	0.239	0.114
VML081	0.229	0.213	0.194	0.150	0.212	0.285	0.303	0.179
VML083	0.222	0.157	0.169	0.202	0.264	0.201	0.232	0.160
VML084	0.253	0.230	0.223	0.141	0.167	0.309	0.322	0.182
VML085	0.134	0.054	0.128	0.193	0.211	0.156	0.149	0.097
VML086	0.274	0.219	0.223	0.249	0.340	0.221	0.275	0.216
VML087	0.194	0.203	0.154	0.123	0.178	0.282	0.267	0.153

VML088	0.184	0.166	0.155	0.187	0.216	0.253	0.246	0.164
VML089	0.156	0.133	0.116	0.222	0.295	0.186	0.195	0.167
VML090	0.223	0.134	0.178	0.259	0.302	0.189	0.211	0.184
VML091	0.187	0.186	0.132	0.075	0.199	0.241	0.251	0.120
VML092	0.142	0.084	0.101	0.167	0.206	0.168	0.153	0.082
VML093	0.230	0.214	0.191	0.110	0.166	0.292	0.300	0.157
VML094	0.234	0.165	0.184	0.218	0.289	0.202	0.245	0.174
VML095	0.165	0.133	0.133	0.136	0.214	0.197	0.226	0.112
VML096	0.277	0.252	0.215	0.145	0.240	0.303	0.323	0.193
VML097	0.167	0.145	0.143	0.117	0.143	0.235	0.233	0.103
VML099	0.203	0.157	0.161	0.182	0.183	0.249	0.231	0.147
VML100	0.149	0.089	0.125	0.198	0.212	0.186	0.166	0.119
VML102	0.114	0.105	0.087	0.106	0.190	0.182	0.187	0.059
VML104	0.229	0.181	0.157	0.202	0.301	0.215	0.249	0.177
VML106	0.214	0.252	0.203	0.075	0.160	0.306	0.299	0.167
VML107	0.187	0.182	0.155	0.068	0.171	0.234	0.246	0.103
VML110	0.219	0.206	0.163	0.114	0.240	0.249	0.277	0.152
VML111	0.320	0.293	0.263	0.182	0.267	0.341	0.369	0.237
VML112	0.228	0.204	0.188	0.141	0.131	0.293	0.276	0.154
VML113	0.073	0.134	0.105	0.173	0.233	0.189	0.157	0.119
VML114	0.295	0.327	0.307	0.240	0.202	0.408	0.384	0.281
VML116	0.301	0.268	0.239	0.193	0.289	0.312	0.347	0.228
VML117	0.139	0.057	0.114	0.210	0.272	0.105	0.131	0.113
VML118	0.249	0.273	0.249	0.178	0.117	0.357	0.322	0.214
VML119	0.270	0.316	0.257	0.132	0.211	0.362	0.353	0.230
VML120	0.147	0.096	0.114	0.232	0.303	0.125	0.153	0.155
VML121	0.163	0.193	0.177	0.177	0.153	0.270	0.220	0.154
VML122	0.137	0.119	0.128	0.125	0.222	0.159	0.198	0.082
VML123	0.084	0.090	0.082	0.197	0.247	0.140	0.097	0.109
VML124	0.325	0.301	0.254	0.224	0.344	0.318	0.355	0.257
VML125	0.443	0.393	0.374	0.327	0.376	0.445	0.470	0.360
VML126	0.150	0.095	0.111	0.217	0.258	0.151	0.127	0.128
VML127	0.166	0.113	0.143	0.174	0.246	0.170	0.213	0.123
VML128	0.141	0.141	0.111	0.113	0.206	0.178	0.183	0.076
VML129	0.259	0.213	0.202	0.195	0.290	0.247	0.291	0.192
VML130	0.167	0.224	0.195	0.124	0.197	0.251	0.251	0.156
VML131	0.256	0.287	0.238	0.117	0.192	0.350	0.350	0.212
VML132	0.201	0.137	0.176	0.188	0.242	0.189	0.232	0.140
VML133	0.319	0.339	0.279	0.167	0.231	0.399	0.395	0.264
VML134	0.118	0.100	0.114	0.127	0.171	0.171	0.159	0.036
VML136	0.304	0.308	0.264	0.216	0.240	0.388	0.377	0.267
VML137	0.230	0.218	0.237	0.176	0.096	0.287	0.260	0.160
VML138	0.230	0.193	0.211	0.142	0.165	0.255	0.275	0.142
VML139	0.167	0.098	0.122	0.214	0.245	0.172	0.152	0.130

VML140	0.112	0.158	0.147	0.172	0.211	0.228	0.211	0.142
VML141	0.163	0.127	0.190	0.212	0.221	0.167	0.178	0.131
VML143	0.158	0.136	0.134	0.120	0.130	0.226	0.207	0.086
VML144	0.151	0.224	0.189	0.153	0.191	0.266	0.238	0.164
VML145	0.231	0.164	0.169	0.288	0.343	0.192	0.188	0.211
VML146		0.101	0.093	0.179	0.247	0.131	0.122	0.106
VML147	0.158		0.106	0.198	0.243	0.111	0.116	0.093
VML148	0.109	0.203		0.156	0.252	0.144	0.136	0.095
VML150	0.044	0.182	0.106		0.159	0.255	0.259	0.111
VML151	0.115	0.200	0.159	0.097		0.328	0.308	0.180
VML152	0.227	0.291	0.168	0.234	0.308		0.092	0.164
VML153	0.177	0.269	0.136	0.183	0.261	0.070		0.163
VML154	0.199	0.161	0.258	0.208	0.173	0.399	0.363	
VML155	0.123	0.086	0.186	0.145	0.178	0.290	0.255	0.148
VML156	0.122	0.264	0.174	0.123	0.193	0.229	0.163	0.308
VML157	0.101	0.226	0.149	0.081	0.117	0.257	0.199	0.253
VML158	0.255	0.335	0.191	0.258	0.335	0.072	0.094	0.433
VML159	0.117	0.256	0.143	0.100	0.105	0.276	0.221	0.237
VML160	0.106	0.177	0.151	0.093	0.060	0.296	0.250	0.173
VML161	0.098	0.128	0.131	0.110	0.171	0.234	0.204	0.184
VML162	0.087	0.174	0.116	0.087	0.086	0.246	0.204	0.198
VML164	0.083	0.123	0.101	0.088	0.116	0.241	0.208	0.170
VML165	0.114	0.261	0.150	0.087	0.126	0.252	0.191	0.272
VML168	0.063	0.136	0.097	0.087	0.145	0.221	0.182	0.186
VML170	0.095	0.212	0.127	0.101	0.162	0.240	0.190	0.243
VML171	0.150	0.305	0.180	0.135	0.178	0.263	0.199	0.317
VML173	0.236	0.260	0.303	0.227	0.164	0.453	0.405	0.146
VML174	0.077	0.192	0.064	0.088	0.164	0.163	0.113	0.259
VML175	0.100	0.074	0.142	0.124	0.152	0.250	0.220	0.156
VML176	0.184	0.274	0.219	0.160	0.091	0.372	0.322	0.207
VML178	0.161	0.186	0.204	0.162	0.132	0.359	0.318	0.095
VML179	0.083	0.205	0.123	0.090	0.178	0.180	0.119	0.274
VML180	0.085	0.216	0.068	0.071	0.147	0.176	0.122	0.266
VML183	0.074	0.160	0.112	0.081	0.145	0.200	0.153	0.241
VML184	0.084	0.158	0.093	0.094	0.157	0.173	0.133	0.250
VML188	0.165	0.164	0.209	0.158	0.118	0.356	0.318	0.108

	VML155	VML156	VML157	VML158	VML159	VML160	VML161	VML162
VML001	0.162	0.290	0.180	0.341	0.276	0.233	0.332	0.375
VML002	0.103	0.175	0.214	0.219	0.168	0.249	0.212	0.261
VML003	0.142	0.256	0.155	0.291	0.252	0.190	0.273	0.352
VML004	0.183	0.234	0.191	0.262	0.224	0.182	0.192	0.309
VML005	0.124	0.103	0.255	0.122	0.085	0.192	0.119	0.231
VML006	0.270	0.122	0.264	0.208	0.197	0.220	0.103	0.097

VML007	0.233	0.305	0.171	0.392	0.281	0.183	0.348	0.348
VML008	0.187	0.206	0.100	0.290	0.215	0.063	0.223	0.258
VML009	0.281	0.270	0.164	0.372	0.275	0.222	0.254	0.234
VML010	0.228	0.085	0.266	0.189	0.134	0.221	0.113	0.088
VML011	0.156	0.177	0.088	0.263	0.193	0.099	0.205	0.237
VML012	0.180	0.097	0.219	0.207	0.141	0.210	0.152	0.132
VML015	0.262	0.136	0.265	0.202	0.205	0.239	0.080	0.131
VML016	0.108	0.188	0.186	0.265	0.161	0.201	0.230	0.256
VML017	0.291	0.202	0.179	0.310	0.257	0.162	0.204	0.158
VML018	0.170	0.158	0.122	0.261	0.194	0.176	0.196	0.186
VML020	0.203	0.124	0.166	0.211	0.181	0.133	0.123	0.169
VML021	0.243	0.146	0.261	0.258	0.175	0.242	0.196	0.114
VML022	0.143	0.157	0.121	0.255	0.163	0.144	0.175	0.198
VML023	0.121	0.163	0.112	0.252	0.163	0.124	0.196	0.227
VML024	0.134	0.177	0.164	0.264	0.149	0.113	0.220	0.247
VML025	0.311	0.162	0.290	0.250	0.241	0.277	0.153	0.063
VML026	0.116	0.153	0.126	0.243	0.158	0.144	0.193	0.219
VML027	0.141	0.183	0.128	0.242	0.186	0.113	0.190	0.263
VML028	0.189	0.104	0.284	0.087	0.143	0.213	0.132	0.233
VML030	0.153	0.231	0.138	0.282	0.203	0.135	0.223	0.306
VML031	0.068	0.146	0.190	0.201	0.135	0.183	0.199	0.254
VML032	0.128	0.244	0.190	0.264	0.237	0.195	0.273	0.356
VML033	0.195	0.232	0.253	0.231	0.213	0.181	0.211	0.337
VML034	0.141	0.168	0.135	0.239	0.177	0.081	0.193	0.252
VML036	0.225	0.361	0.234	0.399	0.341	0.279	0.401	0.452
VML039	0.167	0.096	0.163	0.203	0.136	0.128	0.116	0.150
VML040	0.172	0.252	0.137	0.318	0.249	0.156	0.301	0.330
VML041	0.115	0.159	0.192	0.221	0.092	0.155	0.172	0.240
VML042	0.224	0.250	0.185	0.296	0.247	0.114	0.242	0.321
VML043	0.231	0.135	0.201	0.250	0.209	0.221	0.184	0.113
VML044	0.149	0.215	0.094	0.289	0.226	0.156	0.258	0.284
VML046	0.224	0.266	0.102	0.336	0.270	0.167	0.246	0.300
VML047	0.362	0.351	0.170	0.449	0.399	0.290	0.362	0.318
VML048	0.270	0.180	0.274	0.277	0.229	0.298	0.222	0.133
VML049	0.219	0.156	0.206	0.271	0.164	0.195	0.194	0.138
VML050	0.276	0.185	0.173	0.288	0.253	0.148	0.204	0.172
VML051	0.286	0.151	0.237	0.268	0.231	0.225	0.185	0.060
VML052	0.182	0.194	0.346	0.134	0.181	0.284	0.201	0.324
VML053	0.193	0.142	0.218	0.191	0.160	0.189	0.067	0.196
VML054	0.227	0.218	0.118	0.319	0.247	0.101	0.260	0.248
VML055	0.114	0.182	0.266	0.216	0.149	0.218	0.242	0.296
VML056	0.175	0.139	0.137	0.221	0.197	0.162	0.149	0.191
VML057	0.247	0.186	0.171	0.272	0.228	0.208	0.154	0.167
VML058	0.215	0.088	0.254	0.169	0.139	0.172	0.098	0.152

VML059	0.145	0.172	0.125	0.267	0.168	0.123	0.188	0.218
VML061	0.353	0.215	0.294	0.307	0.293	0.298	0.205	0.098
VML063	0.177	0.152	0.151	0.258	0.144	0.130	0.167	0.174
VML064	0.143	0.166	0.110	0.257	0.170	0.111	0.214	0.231
VML065	0.177	0.143	0.120	0.247	0.192	0.141	0.201	0.189
VML066	0.200	0.199	0.069	0.299	0.224	0.103	0.228	0.228
VML067	0.273	0.141	0.247	0.262	0.199	0.217	0.180	0.055
VML068	0.136	0.168	0.098	0.250	0.169	0.115	0.177	0.229
VML069	0.135	0.119	0.174	0.199	0.158	0.205	0.164	0.191
VML070	0.095	0.112	0.211	0.141	0.112	0.184	0.153	0.239
VML073	0.201	0.155	0.234	0.262	0.149	0.212	0.210	0.168
VML074	0.173	0.141	0.256	0.117	0.174	0.214	0.140	0.263
VML075	0.286	0.264	0.119	0.356	0.318	0.235	0.271	0.249
VML076	0.102	0.236	0.149	0.288	0.200	0.179	0.243	0.317
VML080	0.175	0.175	0.112	0.256	0.189	0.070	0.193	0.235
VML081	0.255	0.170	0.154	0.274	0.234	0.151	0.178	0.154
VML083	0.219	0.079	0.246	0.193	0.142	0.213	0.140	0.096
VML084	0.257	0.193	0.134	0.309	0.240	0.136	0.220	0.170
VML085	0.139	0.098	0.232	0.163	0.076	0.147	0.144	0.210
VML086	0.260	0.159	0.311	0.236	0.214	0.312	0.213	0.155
VML087	0.200	0.194	0.121	0.277	0.207	0.127	0.170	0.222
VML088	0.219	0.155	0.194	0.229	0.181	0.119	0.129	0.197
VML089	0.211	0.119	0.256	0.147	0.165	0.207	0.062	0.194
VML090	0.234	0.091	0.303	0.159	0.119	0.232	0.117	0.140
VML091	0.175	0.155	0.125	0.254	0.191	0.176	0.166	0.178
VML092	0.115	0.103	0.213	0.178	0.054	0.159	0.128	0.198
VML093	0.229	0.181	0.114	0.293	0.220	0.137	0.195	0.168
VML094	0.233	0.094	0.271	0.200	0.161	0.241	0.153	0.095
VML095	0.190	0.086	0.175	0.199	0.165	0.153	0.136	0.137
VML096	0.255	0.198	0.181	0.310	0.239	0.235	0.216	0.146
VML097	0.169	0.138	0.135	0.240	0.156	0.080	0.160	0.187
VML099	0.200	0.143	0.192	0.232	0.132	0.110	0.144	0.182
VML100	0.160	0.109	0.226	0.172	0.082	0.132	0.119	0.203
VML102	0.135	0.110	0.151	0.193	0.143	0.137	0.132	0.192
VML104	0.232	0.110	0.256	0.203	0.174	0.250	0.114	0.091
VML106	0.186	0.257	0.078	0.338	0.267	0.190	0.271	0.298
VML107	0.151	0.168	0.130	0.267	0.189	0.177	0.209	0.208
VML110	0.213	0.156	0.167	0.261	0.215	0.217	0.176	0.146
VML111	0.302	0.233	0.209	0.348	0.285	0.266	0.258	0.157
VML112	0.206	0.191	0.138	0.292	0.181	0.105	0.195	0.206
VML113	0.122	0.191	0.202	0.204	0.182	0.184	0.182	0.299
VML114	0.318	0.344	0.183	0.408	0.353	0.186	0.338	0.379
VML116	0.297	0.198	0.227	0.310	0.265	0.265	0.216	0.107
VML117	0.148	0.075	0.266	0.120	0.088	0.217	0.130	0.197

VML118	0.233	0.295	0.133	0.368	0.275	0.134	0.292	0.339
VML119	0.250	0.318	0.107	0.391	0.332	0.250	0.326	0.349
VML120	0.195	0.071	0.276	0.085	0.132	0.223	0.084	0.187
VML121	0.152	0.240	0.170	0.283	0.200	0.129	0.237	0.326
VML122	0.152	0.108	0.184	0.192	0.170	0.189	0.175	0.191
VML123	0.079	0.152	0.242	0.161	0.107	0.197	0.152	0.273
VML124	0.305	0.237	0.273	0.327	0.289	0.334	0.250	0.177
VML125	0.425	0.321	0.342	0.430	0.362	0.364	0.328	0.191
VML126	0.124	0.124	0.266	0.159	0.049	0.207	0.130	0.228
VML127	0.197	0.064	0.220	0.172	0.157	0.183	0.134	0.136
VML128	0.088	0.151	0.178	0.221	0.148	0.198	0.187	0.232
VML129	0.256	0.140	0.246	0.249	0.213	0.255	0.180	0.085
VML130	0.176	0.241	0.125	0.289	0.267	0.205	0.281	0.324
VML131	0.261	0.278	0.077	0.363	0.310	0.198	0.279	0.288
VML132	0.206	0.087	0.235	0.201	0.157	0.196	0.170	0.133
VML133	0.308	0.314	0.129	0.405	0.340	0.245	0.304	0.296
VML134	0.074	0.140	0.176	0.207	0.118	0.153	0.183	0.239
VML136	0.317	0.283	0.185	0.370	0.307	0.195	0.248	0.261
VML137	0.171	0.249	0.180	0.322	0.209	0.161	0.297	0.317
VML138	0.201	0.169	0.176	0.283	0.195	0.171	0.237	0.191
VML139	0.151	0.108	0.256	0.163	0.034	0.185	0.113	0.200
VML140	0.174	0.199	0.180	0.233	0.214	0.143	0.196	0.291
VML141	0.136	0.170	0.257	0.220	0.154	0.204	0.242	0.277
VML143	0.139	0.147	0.139	0.235	0.131	0.088	0.169	0.214
VML144	0.143	0.268	0.158	0.304	0.258	0.198	0.282	0.361
VML145	0.225	0.146	0.337	0.160	0.115	0.280	0.116	0.206
VML146	0.104	0.159	0.219	0.163	0.167	0.194	0.178	0.285
VML147	0.129	0.093	0.244	0.126	0.094	0.181	0.140	0.224
VML148	0.111	0.121	0.207	0.153	0.129	0.198	0.109	0.225
VML150	0.162	0.194	0.078	0.282	0.217	0.166	0.217	0.235
VML151	0.215	0.261	0.133	0.351	0.244	0.111	0.295	0.303
VML152	0.166	0.138	0.307	0.082	0.162	0.276	0.191	0.275
VML153	0.133	0.172	0.302	0.118	0.137	0.259	0.197	0.311
VML154	0.091	0.124	0.164	0.195	0.125	0.152	0.164	0.221
VML155		0.182	0.210	0.212	0.141	0.201	0.204	0.293
VML156	0.221		0.239	0.126	0.115	0.195	0.109	0.146
VML157	0.186	0.103		0.326	0.261	0.151	0.259	0.271
VML158	0.320	0.234	0.269		0.162	0.278	0.158	0.254
VML159	0.222	0.154	0.130	0.294		0.195	0.139	0.216
VML160	0.146	0.185	0.097	0.318	0.139		0.219	0.250
VML161	0.090	0.199	0.177	0.267	0.186	0.153		0.165
VML162	0.173	0.176	0.135	0.284	0.104	0.112	0.146	
VML164	0.104	0.194	0.139	0.271	0.159	0.090	0.082	0.101
VML165	0.224	0.102	0.084	0.267	0.079	0.142	0.190	0.126

VML168	0.102	0.171	0.150	0.250	0.149	0.130	0.060	0.111
VML170	0.157	0.124	0.106	0.242	0.155	0.130	0.140	0.162
VML171	0.261	0.080	0.108	0.262	0.117	0.184	0.231	0.179
VML173	0.225	0.306	0.233	0.476	0.239	0.165	0.260	0.237
VML174	0.163	0.124	0.119	0.183	0.145	0.147	0.121	0.123
VML175	0.084	0.215	0.181	0.292	0.189	0.140	0.082	0.113
VML176	0.236	0.234	0.153	0.387	0.140	0.109	0.234	0.172
VML178	0.142	0.260	0.200	0.381	0.181	0.124	0.160	0.172
VML179	0.169	0.081	0.108	0.200	0.155	0.164	0.137	0.143
VML180	0.185	0.117	0.102	0.194	0.118	0.140	0.136	0.114
VML183	0.138	0.126	0.090	0.226	0.166	0.117	0.125	0.119
VML184	0.150	0.143	0.117	0.207	0.171	0.135	0.124	0.113
VML188	0.135	0.264	0.184	0.383	0.203	0.095	0.162	0.169

	VML164	VML165	VML168	VML170	VML171	VML173	VML174	VML175
VML001	0.256	0.258	0.306	0.237	0.271	0.224	0.184	0.308
VML002	0.196	0.226	0.180	0.162	0.213	0.188	0.162	0.205
VML003	0.193	0.223	0.247	0.197	0.241	0.173	0.119	0.307
VML004	0.144	0.168	0.187	0.213	0.212	0.165	0.117	0.317
VML005	0.141	0.215	0.096	0.129	0.179	0.180	0.138	0.151
VML006	0.131	0.223	0.129	0.168	0.142	0.169	0.240	0.195
VML007	0.257	0.215	0.334	0.280	0.228	0.232	0.220	0.312
VML008	0.112	0.134	0.214	0.176	0.126	0.101	0.123	0.260
VML009	0.198	0.121	0.268	0.277	0.174	0.187	0.239	0.331
VML010	0.151	0.212	0.124	0.162	0.132	0.183	0.225	0.126
VML011	0.098	0.139	0.185	0.143	0.115	0.076	0.104	0.234
VML012	0.144	0.202	0.144	0.124	0.132	0.149	0.198	0.117
VML015	0.131	0.225	0.114	0.169	0.168	0.169	0.234	0.222
VML016	0.188	0.187	0.213	0.175	0.183	0.176	0.159	0.193
VML017	0.127	0.174	0.218	0.204	0.109	0.129	0.234	0.266
VML018	0.125	0.164	0.182	0.131	0.122	0.091	0.164	0.203
VML020	0.032	0.159	0.120	0.121	0.094	0.075	0.147	0.213
VML021	0.197	0.224	0.199	0.195	0.151	0.205	0.256	0.138
VML022	0.104	0.117	0.170	0.141	0.110	0.090	0.130	0.205
VML023	0.113	0.134	0.179	0.136	0.119	0.095	0.106	0.204
VML024	0.153	0.150	0.210	0.170	0.136	0.152	0.131	0.189
VML025	0.183	0.258	0.174	0.206	0.179	0.204	0.291	0.222
VML026	0.121	0.147	0.175	0.128	0.126	0.103	0.117	0.190
VML027	0.098	0.147	0.167	0.153	0.145	0.102	0.071	0.252
VML028	0.157	0.260	0.104	0.135	0.198	0.198	0.178	0.165
VML030	0.151	0.119	0.213	0.217	0.182	0.158	0.074	0.299
VML031	0.161	0.203	0.170	0.125	0.177	0.158	0.120	0.167
VML032	0.204	0.247	0.241	0.190	0.248	0.193	0.120	0.285
VML033	0.180	0.224	0.199	0.224	0.237	0.215	0.134	0.302

VML034	0.096	0.156	0.178	0.131	0.134	0.098	0.091	0.221
VML036	0.321	0.315	0.373	0.307	0.337	0.291	0.235	0.379
VML039	0.046	0.138	0.110	0.110	0.066	0.079	0.134	0.171
VML040	0.205	0.219	0.273	0.207	0.208	0.177	0.152	0.277
VML041	0.148	0.119	0.159	0.191	0.141	0.173	0.097	0.208
VML042	0.157	0.183	0.234	0.228	0.197	0.174	0.141	0.317
VML043	0.147	0.220	0.177	0.132	0.134	0.131	0.230	0.168
VML044	0.164	0.189	0.231	0.165	0.175	0.127	0.130	0.254
VML046	0.155	0.129	0.244	0.237	0.190	0.141	0.154	0.342
VML047	0.268	0.272	0.359	0.307	0.266	0.218	0.319	0.406
VML048	0.226	0.264	0.217	0.210	0.199	0.221	0.288	0.190
VML049	0.167	0.159	0.196	0.197	0.108	0.172	0.213	0.177
VML050	0.117	0.196	0.211	0.171	0.116	0.112	0.222	0.246
VML051	0.160	0.230	0.194	0.175	0.129	0.162	0.265	0.188
VML052	0.238	0.320	0.178	0.195	0.288	0.271	0.219	0.212
VML053	0.090	0.158	0.085	0.165	0.151	0.141	0.150	0.238
VML054	0.149	0.184	0.252	0.178	0.136	0.119	0.187	0.247
VML055	0.221	0.264	0.217	0.170	0.230	0.227	0.181	0.151
VML056	0.061	0.174	0.133	0.093	0.127	0.040	0.141	0.219
VML057	0.117	0.146	0.163	0.197	0.135	0.125	0.197	0.277
VML058	0.112	0.208	0.112	0.135	0.131	0.160	0.193	0.147
VML059	0.112	0.113	0.189	0.152	0.120	0.102	0.130	0.212
VML061	0.213	0.275	0.225	0.245	0.200	0.222	0.323	0.274
VML063	0.112	0.086	0.168	0.177	0.069	0.124	0.144	0.204
VML064	0.128	0.140	0.196	0.151	0.113	0.112	0.113	0.206
VML065	0.112	0.176	0.182	0.111	0.104	0.080	0.154	0.187
VML066	0.117	0.139	0.218	0.168	0.108	0.082	0.151	0.249
VML067	0.168	0.214	0.190	0.190	0.115	0.181	0.257	0.166
VML068	0.088	0.101	0.165	0.151	0.114	0.083	0.083	0.235
VML069	0.131	0.194	0.135	0.103	0.149	0.120	0.150	0.173
VML070	0.135	0.204	0.106	0.119	0.167	0.155	0.099	0.174
VML073	0.193	0.197	0.206	0.193	0.144	0.197	0.221	0.140
VML074	0.145	0.247	0.104	0.140	0.210	0.180	0.146	0.225
VML075	0.184	0.210	0.267	0.226	0.197	0.137	0.241	0.334
VML076	0.181	0.157	0.227	0.207	0.209	0.171	0.099	0.281
VML080	0.085	0.127	0.178	0.158	0.103	0.089	0.101	0.242
VML081	0.090	0.168	0.183	0.161	0.095	0.086	0.200	0.243
VML083	0.152	0.211	0.141	0.148	0.125	0.172	0.218	0.115
VML084	0.126	0.158	0.222	0.186	0.085	0.107	0.209	0.244
VML085	0.130	0.190	0.124	0.135	0.135	0.166	0.135	0.130
VML086	0.238	0.301	0.205	0.190	0.225	0.240	0.294	0.140
VML087	0.083	0.089	0.178	0.183	0.121	0.092	0.134	0.273
VML088	0.070	0.145	0.143	0.168	0.113	0.122	0.154	0.237
VML089	0.111	0.215	0.069	0.147	0.171	0.163	0.178	0.213

VML090	0.175	0.240	0.121	0.181	0.163	0.218	0.232	0.122
VML091	0.100	0.142	0.162	0.135	0.120	0.076	0.157	0.220
VML092	0.123	0.156	0.115	0.142	0.128	0.155	0.123	0.145
VML093	0.101	0.127	0.197	0.173	0.078	0.082	0.182	0.242
VML094	0.172	0.243	0.151	0.151	0.153	0.189	0.244	0.102
VML095	0.073	0.183	0.124	0.081	0.089	0.081	0.165	0.156
VML096	0.174	0.184	0.219	0.203	0.145	0.153	0.242	0.242
VML097	0.065	0.117	0.158	0.133	0.067	0.079	0.124	0.195
VML099	0.105	0.102	0.152	0.189	0.075	0.148	0.143	0.210
VML100	0.111	0.158	0.114	0.157	0.121	0.161	0.127	0.169
VML102	0.056	0.154	0.106	0.093	0.106	0.073	0.103	0.186
VML104	0.152	0.222	0.124	0.155	0.157	0.171	0.234	0.167
VML106	0.178	0.176	0.260	0.202	0.202	0.133	0.169	0.303
VML107	0.140	0.169	0.194	0.136	0.139	0.108	0.161	0.198
VML110	0.131	0.190	0.173	0.138	0.139	0.107	0.207	0.210
VML111	0.212	0.227	0.261	0.234	0.177	0.187	0.288	0.267
VML112	0.122	0.056	0.201	0.208	0.083	0.133	0.151	0.247
VML113	0.142	0.202	0.151	0.164	0.206	0.161	0.085	0.262
VML114	0.235	0.224	0.332	0.313	0.255	0.225	0.233	0.413
VML116	0.190	0.232	0.224	0.208	0.166	0.176	0.283	0.241
VML117	0.157	0.235	0.096	0.124	0.174	0.187	0.174	0.106
VML118	0.197	0.139	0.290	0.272	0.205	0.189	0.165	0.350
VML119	0.235	0.220	0.311	0.267	0.257	0.189	0.218	0.377
VML120	0.141	0.242	0.062	0.130	0.179	0.184	0.185	0.157
VML121	0.168	0.146	0.224	0.227	0.196	0.182	0.081	0.297
VML122	0.125	0.221	0.141	0.062	0.146	0.112	0.162	0.148
VML123	0.157	0.204	0.130	0.155	0.202	0.187	0.109	0.199
VML124	0.248	0.278	0.252	0.245	0.239	0.232	0.315	0.271
VML125	0.319	0.307	0.348	0.359	0.265	0.316	0.403	0.347
VML126	0.166	0.200	0.125	0.167	0.184	0.203	0.156	0.150
VML127	0.109	0.221	0.114	0.079	0.121	0.125	0.188	0.122
VML128	0.151	0.189	0.165	0.124	0.171	0.141	0.138	0.178
VML129	0.176	0.241	0.181	0.162	0.158	0.173	0.260	0.167
VML130	0.194	0.237	0.247	0.183	0.224	0.157	0.147	0.297
VML131	0.175	0.185	0.271	0.231	0.198	0.131	0.206	0.346
VML132	0.149	0.232	0.158	0.112	0.130	0.157	0.214	0.082
VML133	0.220	0.187	0.307	0.289	0.224	0.187	0.254	0.385
VML134	0.136	0.173	0.155	0.130	0.148	0.141	0.099	0.170
VML136	0.176	0.156	0.270	0.278	0.189	0.180	0.244	0.362
VML137	0.225	0.189	0.276	0.244	0.195	0.215	0.162	0.259
VML138	0.171	0.200	0.225	0.160	0.128	0.150	0.206	0.163
VML139	0.148	0.177	0.113	0.171	0.151	0.192	0.154	0.150
VML140	0.120	0.198	0.174	0.163	0.187	0.136	0.109	0.273
VML141	0.210	0.263	0.215	0.157	0.211	0.214	0.187	0.130

VML143	0.095	0.098	0.158	0.157	0.087	0.113	0.089	0.204
VML144	0.203	0.222	0.258	0.210	0.249	0.182	0.126	0.317
VML145	0.214	0.250	0.132	0.224	0.223	0.260	0.240	0.181
VML146	0.148	0.232	0.142	0.120	0.209	0.162	0.108	0.216
VML147	0.138	0.220	0.102	0.119	0.165	0.171	0.136	0.138
VML148	0.113	0.184	0.083	0.119	0.171	0.139	0.111	0.201
VML150	0.126	0.155	0.199	0.150	0.145	0.082	0.140	0.248
VML151	0.198	0.159	0.284	0.243	0.169	0.182	0.173	0.286
VML152	0.218	0.304	0.147	0.154	0.250	0.238	0.201	0.168
VML153	0.223	0.274	0.159	0.197	0.255	0.251	0.162	0.212
VML154	0.111	0.169	0.131	0.108	0.133	0.116	0.095	0.173
VML155	0.177	0.203	0.177	0.162	0.210	0.183	0.106	0.212
VML156	0.124	0.215	0.085	0.107	0.131	0.155	0.174	0.115
VML157	0.153	0.152	0.246	0.201	0.160	0.111	0.156	0.300
VML158	0.212	0.300	0.121	0.179	0.244	0.248	0.215	0.187
VML159	0.166	0.188	0.126	0.179	0.162	0.205	0.153	0.147
VML160	0.124	0.139	0.214	0.189	0.115	0.136	0.136	0.241
VML161	0.120	0.196	0.062	0.158	0.162	0.170	0.186	0.196
VML162	0.177	0.236	0.179	0.199	0.144	0.194	0.276	0.188
VML164		0.140	0.117	0.114	0.092	0.062	0.128	0.207
VML165	0.169		0.206	0.233	0.127	0.157	0.146	0.279
VML168	0.064	0.162		0.134	0.159	0.161	0.161	0.183
VML170	0.122	0.146	0.110		0.162	0.109	0.168	0.154
VML171	0.212	0.076	0.198	0.142		0.111	0.161	0.194
VML173	0.225	0.260	0.254	0.256	0.295		0.143	0.224
VML174	0.107	0.131	0.083	0.095	0.152	0.299		0.244
VML175	0.074	0.201	0.073	0.167	0.247	0.247	0.134	
VML176	0.184	0.163	0.212	0.195	0.196	0.137	0.225	0.230
VML178	0.132	0.220	0.146	0.178	0.256	0.142	0.208	0.154
VML179	0.142	0.115	0.115	0.114	0.136	0.303	0.067	0.154
VML180	0.118	0.094	0.106	0.111	0.128	0.291	0.049	0.154
VML183	0.096	0.135	0.103	0.108	0.167	0.268	0.073	0.121
VML184	0.095	0.148	0.100	0.125	0.181	0.290	0.064	0.115
VML188	0.121	0.219	0.163	0.193	0.267	0.125	0.213	0.149

	VML176	VML178	VML179	VML180	VML183	VML184	VML188
VML001	0.210	0.266	0.193	0.366	0.175	0.236	0.334
VML002	0.206	0.148	0.121	0.252	0.134	0.202	0.262
VML003	0.150	0.243	0.167	0.342	0.110	0.199	0.282
VML004	0.151	0.212	0.163	0.283	0.133	0.190	0.208
VML005	0.124	0.103	0.180	0.194	0.140	0.178	0.168
VML006	0.217	0.149	0.229	0.112	0.220	0.155	0.135
VML007	0.214	0.273	0.220	0.334	0.241	0.222	0.297
VML008	0.099	0.192	0.152	0.249	0.134	0.110	0.175

VML009	0.264	0.224	0.166	0.228	0.239	0.185	0.228
VML010	0.209	0.090	0.202	0.067	0.214	0.167	0.138
VML011	0.104	0.166	0.115	0.236	0.100	0.098	0.179
VML012	0.193	0.078	0.157	0.136	0.169	0.147	0.178
VML015	0.225	0.153	0.218	0.139	0.207	0.164	0.160
VML016	0.176	0.138	0.124	0.236	0.150	0.177	0.240
VML017	0.210	0.205	0.214	0.185	0.220	0.121	0.152
VML018	0.177	0.136	0.103	0.202	0.125	0.108	0.203
VML020	0.131	0.140	0.155	0.174	0.129	0.088	0.118
VML021	0.246	0.126	0.207	0.117	0.240	0.189	0.194
VML022	0.142	0.110	0.080	0.187	0.111	0.100	0.173
VML023	0.114	0.131	0.091	0.216	0.098	0.104	0.185
VML024	0.108	0.139	0.144	0.217	0.150	0.140	0.185
VML025	0.280	0.183	0.252	0.125	0.261	0.196	0.190
VML026	0.129	0.120	0.089	0.210	0.098	0.113	0.191
VML027	0.085	0.173	0.124	0.251	0.085	0.124	0.180
VML028	0.154	0.155	0.227	0.213	0.172	0.197	0.179
VML030	0.126	0.199	0.120	0.274	0.125	0.168	0.207
VML031	0.134	0.126	0.128	0.236	0.105	0.162	0.223
VML032	0.141	0.240	0.187	0.342	0.119	0.212	0.284
VML033	0.129	0.232	0.226	0.301	0.172	0.223	0.212
VML034	0.050	0.160	0.144	0.238	0.093	0.111	0.169
VML036	0.259	0.341	0.262	0.440	0.240	0.302	0.395
VML039	0.120	0.088	0.125	0.142	0.121	0.080	0.111
VML040	0.152	0.243	0.182	0.325	0.159	0.185	0.273
VML041	0.133	0.110	0.103	0.190	0.145	0.161	0.166
VML042	0.117	0.245	0.213	0.300	0.174	0.183	0.197
VML043	0.222	0.142	0.179	0.165	0.186	0.139	0.199
VML044	0.149	0.203	0.129	0.287	0.112	0.145	0.249
VML046	0.187	0.237	0.145	0.291	0.159	0.166	0.228
VML047	0.333	0.340	0.264	0.361	0.284	0.244	0.340
VML048	0.295	0.171	0.218	0.172	0.254	0.221	0.250
VML049	0.213	0.118	0.158	0.123	0.211	0.153	0.170
VML050	0.188	0.202	0.217	0.201	0.199	0.108	0.160
VML051	0.243	0.168	0.227	0.133	0.235	0.152	0.176
VML052	0.202	0.207	0.274	0.292	0.212	0.275	0.267
VML053	0.163	0.129	0.154	0.169	0.146	0.147	0.135
VML054	0.147	0.212	0.194	0.258	0.176	0.124	0.203
VML055	0.159	0.168	0.214	0.269	0.181	0.222	0.256
VML056	0.145	0.145	0.126	0.209	0.086	0.094	0.174
VML057	0.222	0.173	0.142	0.180	0.177	0.137	0.173
VML058	0.150	0.113	0.213	0.129	0.185	0.145	0.106
VML059	0.126	0.122	0.106	0.198	0.120	0.105	0.172
VML061	0.316	0.232	0.277	0.170	0.293	0.210	0.229

VML063	0.150	0.108	0.108	0.146	0.158	0.105	0.135
VML064	0.119	0.146	0.107	0.220	0.117	0.110	0.186
VML065	0.148	0.147	0.132	0.207	0.122	0.090	0.188
VML066	0.142	0.185	0.138	0.235	0.141	0.093	0.187
VML067	0.236	0.147	0.220	0.096	0.242	0.158	0.159
VML068	0.111	0.138	0.072	0.214	0.086	0.098	0.168
VML069	0.176	0.110	0.103	0.198	0.106	0.137	0.207
VML070	0.123	0.123	0.136	0.221	0.099	0.164	0.190
VML073	0.213	0.116	0.179	0.148	0.218	0.179	0.196
VML074	0.151	0.176	0.201	0.249	0.134	0.196	0.198
VML075	0.263	0.257	0.184	0.287	0.200	0.170	0.268
VML076	0.150	0.190	0.107	0.289	0.117	0.186	0.249
VML080	0.089	0.170	0.134	0.226	0.118	0.099	0.150
VML081	0.183	0.179	0.181	0.183	0.176	0.093	0.147
VML083	0.206	0.092	0.189	0.094	0.200	0.156	0.156
VML084	0.188	0.190	0.182	0.195	0.195	0.101	0.165
VML085	0.098	0.099	0.175	0.174	0.153	0.154	0.137
VML086	0.291	0.166	0.247	0.184	0.257	0.241	0.259
VML087	0.145	0.165	0.122	0.208	0.133	0.105	0.152
VML088	0.126	0.154	0.181	0.175	0.162	0.117	0.093
VML089	0.169	0.143	0.201	0.176	0.165	0.165	0.145
VML090	0.208	0.115	0.234	0.102	0.233	0.199	0.142
VML091	0.176	0.126	0.090	0.187	0.116	0.103	0.184
VML092	0.119	0.056	0.132	0.155	0.138	0.146	0.140
VML093	0.176	0.166	0.144	0.184	0.167	0.085	0.158
VML094	0.227	0.111	0.214	0.114	0.219	0.181	0.178
VML095	0.145	0.112	0.154	0.157	0.132	0.092	0.138
VML096	0.255	0.172	0.161	0.179	0.212	0.157	0.218
VML097	0.095	0.119	0.131	0.173	0.125	0.071	0.120
VML099	0.132	0.121	0.154	0.136	0.176	0.126	0.074
VML100	0.099	0.099	0.168	0.155	0.156	0.145	0.101
VML102	0.105	0.109	0.109	0.188	0.079	0.095	0.147
VML104	0.235	0.108	0.188	0.111	0.202	0.170	0.178
VML106	0.194	0.223	0.132	0.301	0.138	0.162	0.269
VML107	0.171	0.137	0.106	0.215	0.125	0.123	0.215
VML110	0.217	0.142	0.140	0.181	0.158	0.125	0.206
VML111	0.294	0.217	0.210	0.208	0.255	0.189	0.252
VML112	0.152	0.153	0.131	0.177	0.176	0.118	0.134
VML113	0.118	0.186	0.155	0.278	0.095	0.182	0.216
VML114	0.229	0.336	0.267	0.374	0.254	0.242	0.277
VML116	0.284	0.193	0.214	0.173	0.245	0.177	0.226
VML117	0.163	0.095	0.187	0.175	0.165	0.186	0.178
VML118	0.173	0.262	0.188	0.316	0.200	0.194	0.239
VML119	0.262	0.292	0.174	0.360	0.193	0.220	0.326

VML120	0.176	0.123	0.207	0.169	0.171	0.183	0.162
VML121	0.108	0.211	0.156	0.289	0.145	0.187	0.211
VML122	0.156	0.131	0.151	0.211	0.114	0.130	0.203
VML123	0.122	0.127	0.159	0.233	0.122	0.192	0.199
VML124	0.336	0.220	0.227	0.223	0.270	0.244	0.292
VML125	0.404	0.305	0.330	0.231	0.388	0.302	0.305
VML126	0.153	0.078	0.174	0.173	0.168	0.195	0.172
VML127	0.160	0.116	0.187	0.157	0.157	0.124	0.155
VML128	0.158	0.112	0.105	0.221	0.104	0.155	0.221
VML129	0.254	0.145	0.206	0.142	0.221	0.172	0.207
VML130	0.186	0.246	0.158	0.333	0.116	0.185	0.290
VML131	0.226	0.263	0.174	0.308	0.180	0.164	0.263
VML132	0.182	0.116	0.202	0.150	0.188	0.148	0.171
VML133	0.285	0.288	0.194	0.312	0.237	0.206	0.287
VML134	0.106	0.115	0.116	0.218	0.104	0.142	0.192
VML136	0.244	0.262	0.227	0.258	0.245	0.185	0.203
VML137	0.169	0.220	0.177	0.291	0.201	0.204	0.255
VML138	0.184	0.155	0.176	0.201	0.189	0.139	0.208
VML139	0.147	0.069	0.170	0.138	0.176	0.175	0.135
VML140	0.099	0.206	0.180	0.280	0.112	0.163	0.194
VML141	0.153	0.167	0.218	0.258	0.184	0.207	0.244
VML143	0.090	0.118	0.104	0.185	0.120	0.103	0.134
VML144	0.160	0.248	0.168	0.348	0.119	0.209	0.290
VML145	0.241	0.125	0.237	0.142	0.246	0.247	0.188
VML146	0.117	0.168	0.169	0.270	0.085	0.183	0.222
VML147	0.118	0.115	0.179	0.200	0.139	0.171	0.168
VML148	0.143	0.105	0.118	0.203	0.085	0.157	0.182
VML150	0.165	0.171	0.092	0.245	0.104	0.115	0.220
VML151	0.160	0.234	0.186	0.287	0.203	0.173	0.231
VML152	0.209	0.178	0.230	0.262	0.182	0.245	0.258
VML153	0.189	0.180	0.212	0.273	0.177	0.255	0.251
VML154	0.107	0.110	0.106	0.209	0.085	0.125	0.180
VML155	0.137	0.147	0.133	0.263	0.109	0.193	0.238
VML156	0.164	0.090	0.177	0.138	0.159	0.149	0.145
VML157	0.180	0.222	0.130	0.280	0.143	0.133	0.235
VML158	0.222	0.180	0.246	0.234	0.207	0.249	0.230
VML159	0.154	0.083	0.169	0.158	0.179	0.189	0.157
VML160	0.088	0.185	0.179	0.228	0.166	0.124	0.145
VML161	0.186	0.104	0.183	0.133	0.175	0.166	0.140
VML162	0.261	0.160	0.235	0.096	0.254	0.180	0.170
VML164	0.115	0.122	0.133	0.173	0.108	0.081	0.120
VML165	0.169	0.164	0.121	0.196	0.179	0.147	0.155
VML168	0.172	0.108	0.166	0.162	0.148	0.164	0.154
VML170	0.145	0.135	0.175	0.215	0.110	0.132	0.197

VML171	0.147	0.128	0.147	0.139	0.168	0.092	0.105
VML173	0.136	0.152	0.128	0.210	0.100	0.074	0.170
VML174	0.096	0.159	0.104	0.247	0.085	0.151	0.188
VML175	0.208	0.137	0.247	0.179	0.228	0.212	0.209
VML176		0.156	0.168	0.234	0.120	0.136	0.160
VML178	0.148		0.139	0.119	0.151	0.143	0.142
VML179	0.238	0.233		0.218	0.104	0.142	0.202
VML180	0.203	0.212	0.072		0.248	0.183	0.133
VML183	0.212	0.205	0.074	0.085		0.133	0.207
VML184	0.230	0.216	0.082	0.082	0.036		0.167
VML188	0.142	0.084	0.231	0.211	0.185	0.200	
